# Supplementary material for: Carbon dioxide electroreduction to C2 products over copper-cuprous oxide derived from electrosynthesized copper complex
Source: Nat Commun. 2019 Aug 26;10:3851. doi: 10.1038/s41467-019-11599-7 (PMC6710288; doi:10.1038/s41467-019-11599-7)
Supplement: Supplementary file 1 — Supplementary information [file 41467_2019_11599_MOESM1_ESM.pdf]

Supplementary Information

Carbon dioxide electroreduction to C_2 products over copper-cuprous oxide derived from electrosynthesized copper complex

Zhu *et al*.

Supplementary note 1. In situ electrodeposition and characterization of Cu-complexes

1.1 SEM images of Cu-Complexes and neat Cu foil (Fig. 1-6)……………………………..………4

1.2 Characterization of the Cu-Complexes (FT-IR Fig. 7; PXRD Fig. 8; XPS Fig. 9, nitrogen adsorption/desorption isotherms Fig. 10, Table 1; ICP Table 2) ………………….………………7

1.3 Determination of the Cu-Complex loading on Cu substrate (Table 3) ……………...…………14

1.4 Characterization of the Cu-Complexes by SAXS technique (Fig. 11) ……………....………….15

1.5 Electrochemical capacitance analysis (Fig. 12, Table 4) ………………………..……………16

1.6 Crystal characterization of the Cu-Complexes (Fig. 13-18; Table 15-21)…………………18

Supplementary note 2. In situ formation and characterization of Cu-Cu_2O catalysts via electroreduction of the Cu-Complexes on the Cu substrate

2.1 SEM images of various Cu-Cu_2O electrodes (Fig. 19) ……………………………………….21

2.2 Characterizations of various Cu-Cu_2O electrodes (PXRD Fig. 20; XPS Fig. 21, Table 5) ……………………………………………………………………………………….….23

2.3 XAFS analysis (Fig. 22-23; Table 6) ………………………..……………...………………26

Supplementary note 3. Electrochemical reduction of CO_2 over Cu-Cu_2O-1 to Cu-Cu_2O-6 electrodes

3.1 LSV profiles over various Cu-Cu_2O catalysts and neat Cu substrate (Fig. 24)…………………30

3.2 Effect of electrolyte concentration (Fig. 25) ………………...…………………..…...……………31

3.3 GC chromatographs of the gaseous products (Fig. 26) ……………..……………………………32

3.4 Comparison study of the other Cu based electrodes (SEM and XPS Fig. 27) ……………...……33
3.5 Product distribution over $Cu-Cu_2O$ electrodes (Table 7) ……………………….……..…..……34
3.6 Overview of Cu based and other catalysts for reduction of CO_2 (Table 8) …………..…..……35
3.7 Long-term stability of the $Cu-Cu_2O$ electrodes in the electrolysis (Fig. 28)…….….…………...39
3.8 Production rate and TOF of C_2 products over $Cu-Cu_2O$ electrodes (Fig. 29; Table 9, 10) …40
3.9 Effect of different electrolytes (Fig. 30-32, Table 11) ……………………...………………43
3.10 $^{12}CO_2$ and $^{13}CO_2$ NMR characterization after electrolysis (Fig. 33-35) ………………………47

Supplementary note 4. Performance of $Cu-Cu_2O$ electrodes prepared by different methods

4.1 Characterization of Cu-Complexes prepared by solvothermal method (Fig. 36, 37)…..……..……50
4.2 Characterization of the catalysts before and after electrolysis (Fig. 38) ……..……………………52
4.3 Reduction of CO_2 over $Cu-Cu_2O$ via Cu-Complexes prepared by solvothermal method (Fig. 39, 40; Table 12) ………..……………………...…………..………………………………………53
4.4 EIS study of the two methods (Fig. 41; Table 13) ……………………………………...……56
4.5 Reduction of CO_2 over $Cu-Cu_2O$ derived from Cu-Complex with different electrodeposition time (Fig. 42) ……………………………..………………………………………………………59

Supplementary note 5. Electrochemical reduction of CO_2 over other $Cu-Cu_2O$ electrodes

5.1 Current densities and selectivities of C_2 products over various $Cu-Cu_2O$ electrodes (Fig. 43, 44)…………………………………………..…………………………………………..……60
5.2 Production rate of C_2 products over various $Cu-Cu_2O$ electrodes (Fig. 45)…………...….……62

Supplementary note 6. Proposed reaction mechanism for the C-C bond formation

6.1 Possible reaction intermediates (Fig. 46, Table 14) ………………….……..………………65
6.2 Possible reaction mechanism (Scheme 2) …………………………..….…………………...67

Supplementary note 7. Crystallogrphic data (Table 15-21) …………………………68

Supplementary references……………………………………...………………………………78

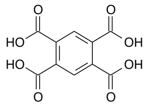

1,2,4,5-H_4BTC
(Ligand-1)

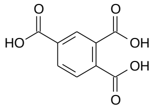

1,2,4-H_3BTC
(Ligand-2)

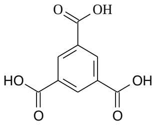

1,3,5-H_3BTC
(Ligand-3)

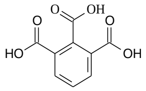

1,2,3-H_3BTC
(Ligand-4)

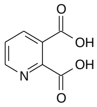

2,6-H_2PyDC
(Ligand-5)

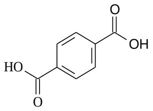

1,4-H_2BDC
(Ligand-6)

Supplementary Scheme 1. The structures of the ligands used.

Supplementary note 1. In situ electrodeposition and characterization of Cu-Complexes

1.1 SEM images of Cu-Complexes and neat Cu foil

The SEM images of Complex-1 are shown in Fig. 1. Complex-2 to Complex-6 electrodes and neat Cu foil were also characterized by SEM technique, and the images are given in Supplementary Figs. 1-5. The morphologies of the complexes on Cu substrate were similar to that of Complex-1, which had 3D hierarchical structures with thickness of 20~80 µm. The neat Cu substrate had no 3D hierarchical structure.

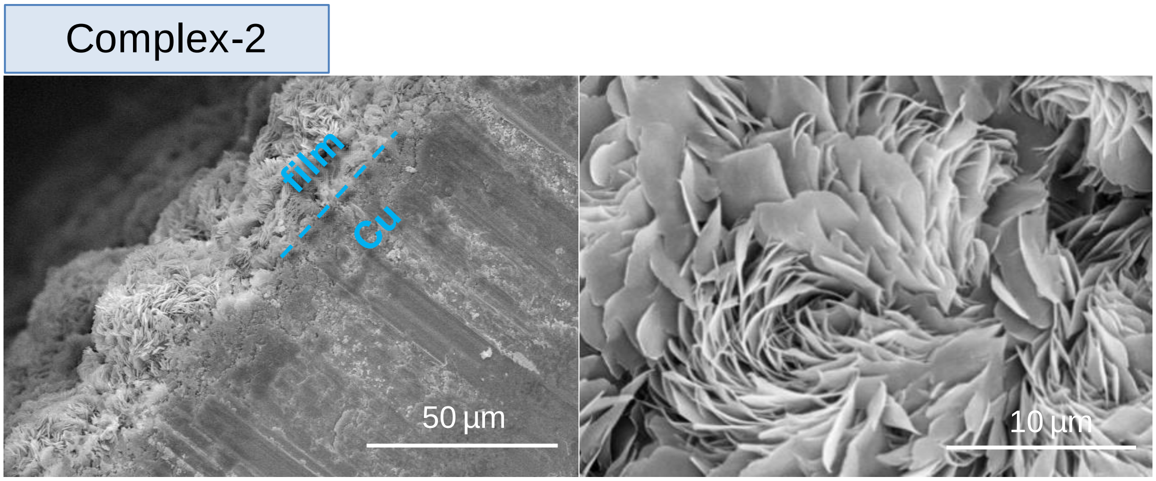

Supplementary Figure 1. SEM images of Complex-2 formed on Cu substrate by electrosynthesis at 9 V for 1 h. (A) side-view and **(B)** top-view.

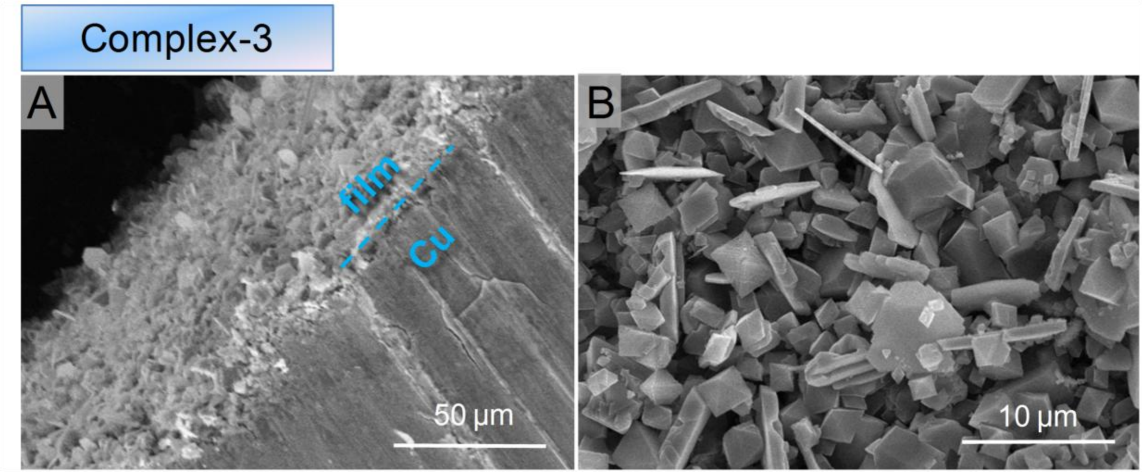

Supplementary Figure 2. SEM images of Complex-3 formed on Cu substrate by electrosynthesis at 9 V for 1 h. (A) side-view and **(B)** top-view.

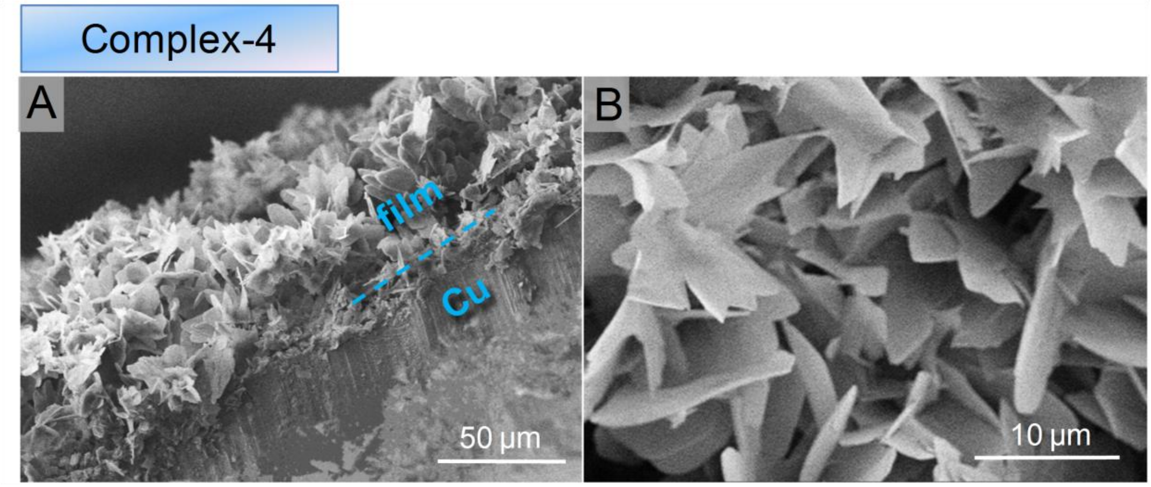

Supplementary Figure 3. SEM images of Complex-4 formed on Cu substrate by electrosynthesis at 9 V for 1 h. (A) side-view and **(B)** top-view.

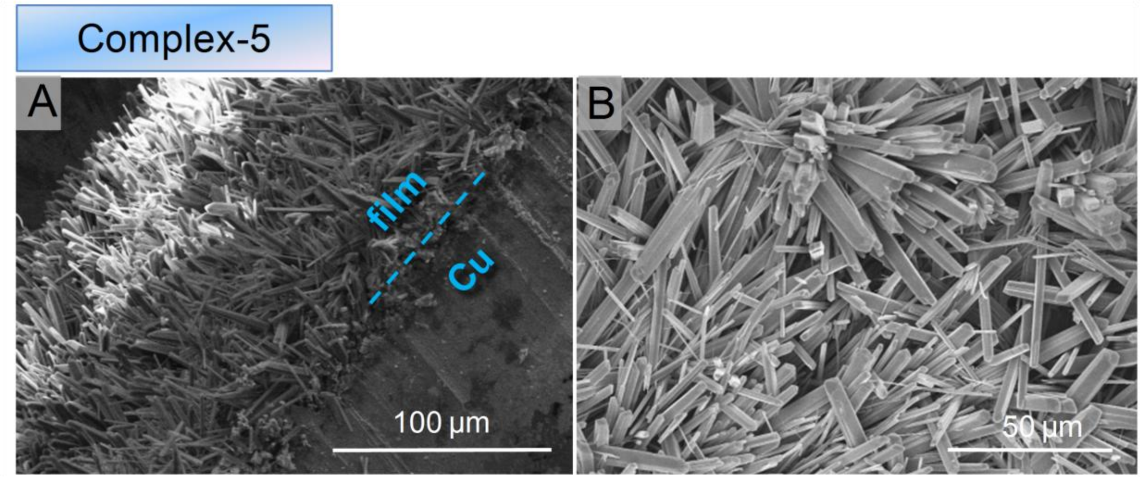

Supplementary Figure 4. SEM images of Complex-5 formed on Cu substrate by electrosynthesis at 9 V for 1 h. (A) side-view and **(B)** top-view.

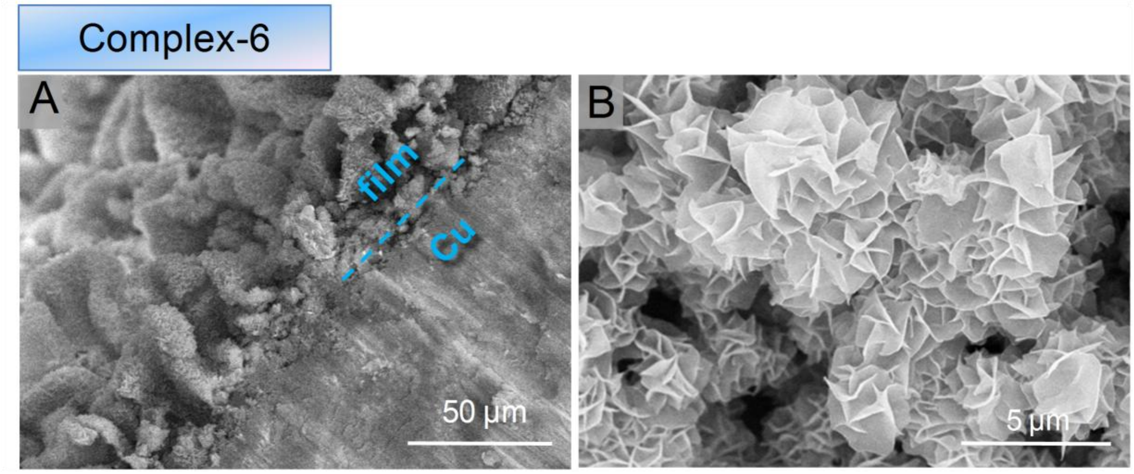

Supplementary Figure 5. SEM images of Complex-6 formed on Cu substrate by electrosynthesis at 9 V for 1 h. (A) side-view and **(B)** top-view.

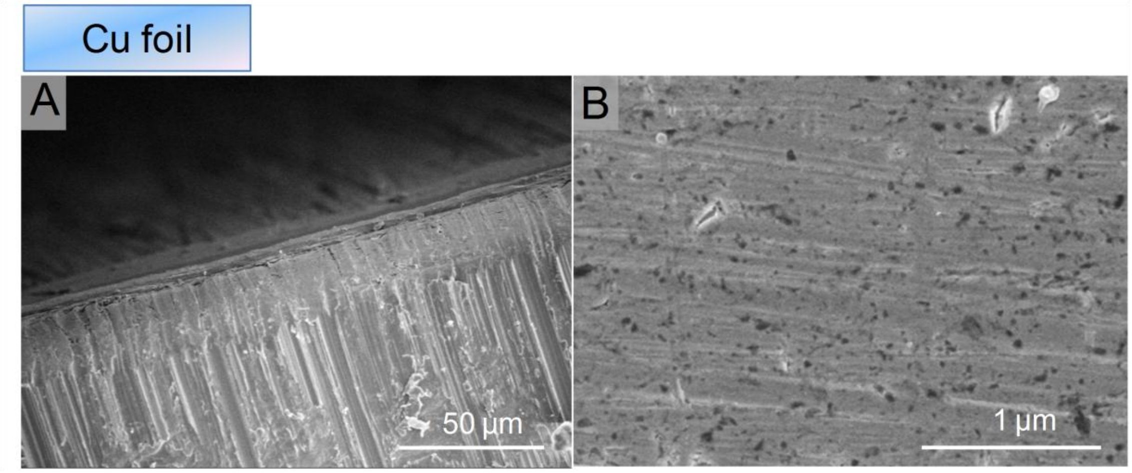

Supplementary Figure 6. SEM images of neat Cu substrate. (A) side-view and **(B)** top-view.

1.2 Characterization of the Cu-Complexes by FT-IR, PXRD, XPS, nitrogen adsorption/desorption, and ICP techniques

The complexes formed on Cu substrate by electrosynthesis at 9 V for 1 h were characterized by Fourier transform infrared (FT-IR), X-ray photoelectron spectroscopy (XPS), XRD and nitrogen adsorption/desorption techniques. Their FT-IR spectra of the complexes had similar features (Supplementary Fig. 7). The strong absorption peaks appeared in the wavelength range of 1667–1634 and 1388–1378 cm^{-1} are originated from the asymmetric and symmetric stretching vibrations of carboxylate group in Cu-Complexes, respectively. Other bonds located at the ranges of 1723–1607 and 1455–1404 cm^{-1} are from free COO^{-} functional groups. The bathochromic effect evidences the coordination of organic ligands with Cu centers. The XRD patterns of complexes and the corresponding ligands are presented in Supplementary Fig. 8. The XRD patterns indicate that the complexes had crystal structures[1-7]. The Cu XPS spectra of the complexes are presented in Supplementary Fig. 9. The binding energy (BE) peak at ~935 eV is assigned to Cu^{II} in the spinel, accompanied by the characteristic Cu^{II} shakeup satellite peaks (938-945 eV)[8]. The result indicates the divalent nature of Cu atoms in the electrosynthesized Cu-Complexes. The BET surface areas and pore volume data obtained from the nitrogen adsorption/desorption isotherms (Supplementary Figs. 10) are given in Supplementary Table 1, which indicate that the complexes are porous with large surface areas.

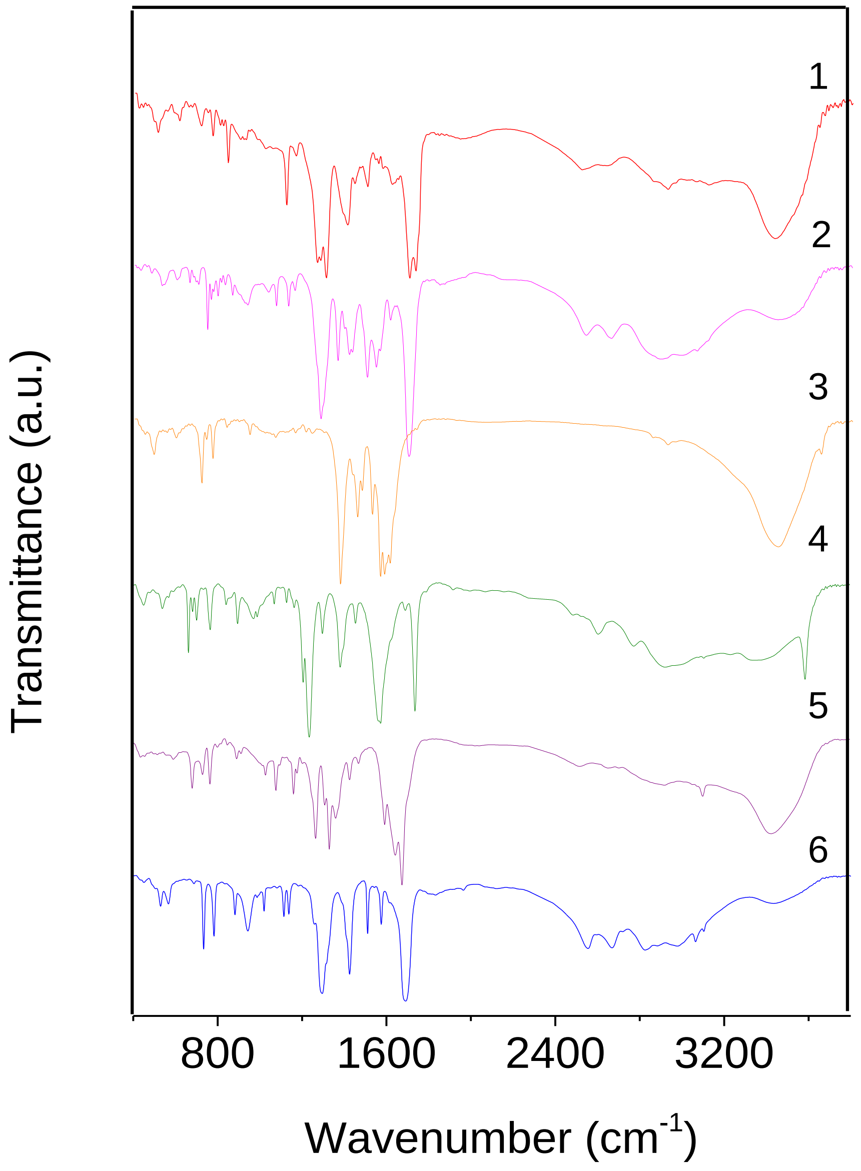

Supplementary Figure 7. FT-IR spectrum of Cu-Complexes. The FT-IR spectra indicate the formation of complexes.

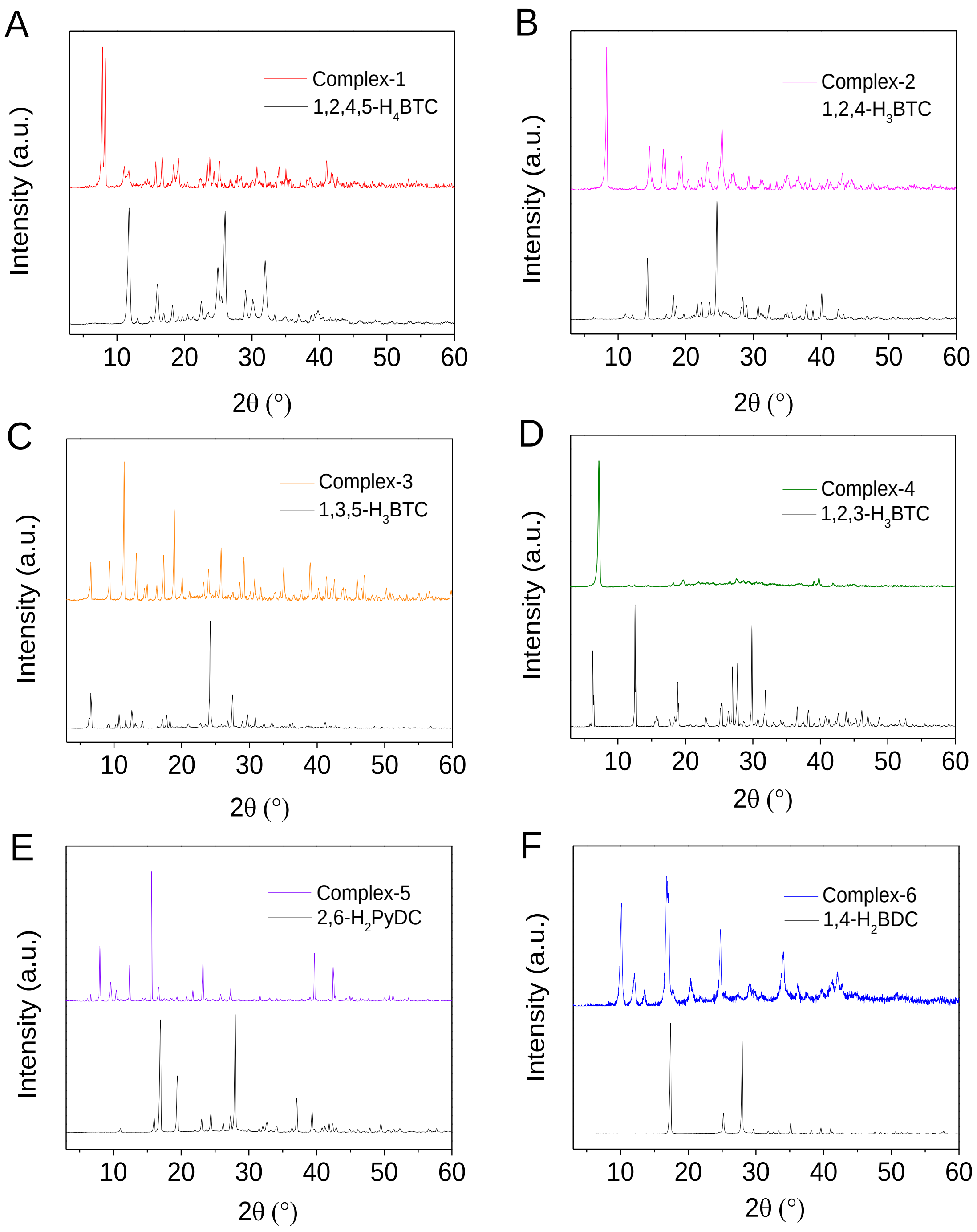

Supplementary Figure 8. XRD patterns of the Cu-Complexes and the corresponding ligands.

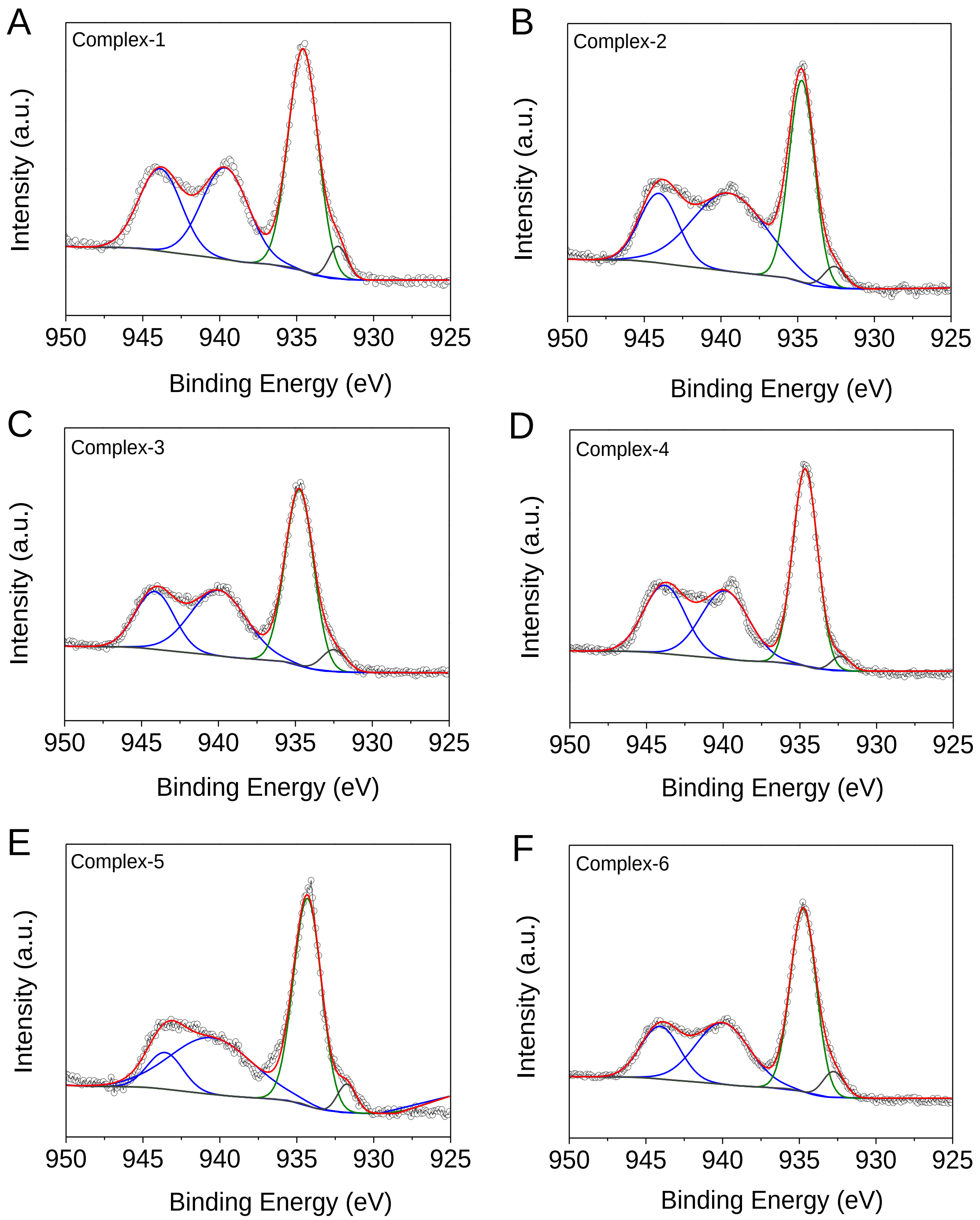

Supplementary Figure 9. Cu $2p_{3/2}$ XPS spectra of the Cu-Complexes.

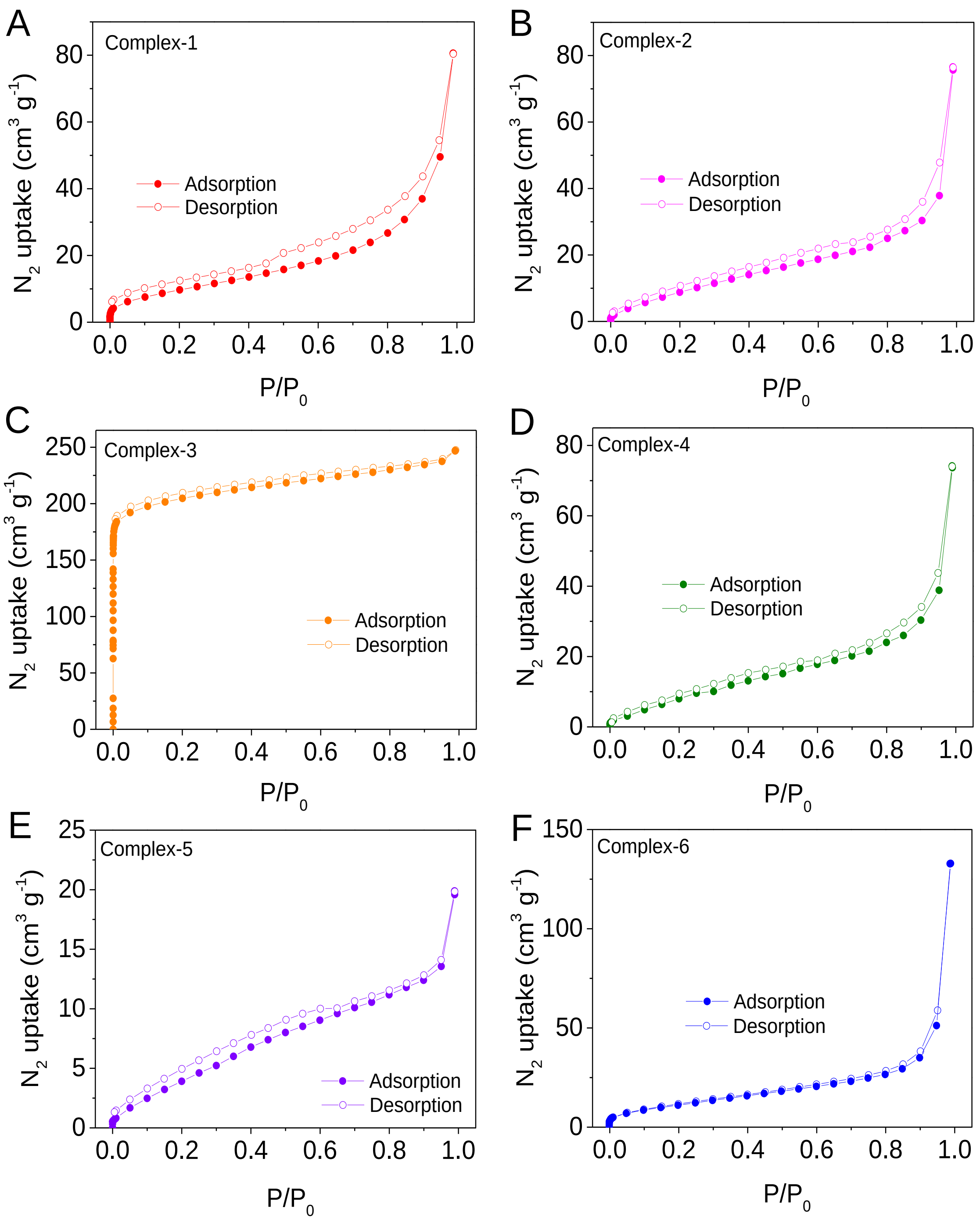

Supplementary Figure 10. Nitrogen adsorption/desorption isotherm of the Cu-Complexes.

Supplementary Table 1. The BET surface areas and total pore volume (V) of the synthesized Complexes.

Entry	Sample	S (m^2/g)	V (cm^3/g)
1	Complex-1	98.2	0.13
2	Complex-2	73.3	0.13
3	Complex-3	596.7	0.06
4	Complex-4	70.5	0.14
5	Complex-5	34.6	0.23
6	Complex-6	120.9	0.20
7	Complex-1<br>(Solvothermal method)	620.8	0.05
8	Complex-3<br>(Solvothermal method)	75.5	0.23

Supplementary Table 2. Cu content in the complexes and Cu: ligand molar ratios in the complexes.

Entry	Sample	Cu content (%)	C content (%)	Cu: ligand ratios
1	Complex-1	27.19	19.10	2.69
2	Complex-2	26.40	32.16	1.40
3	Complex-3	21.39	28.66	1.27
4	Complex-4	19.54	33.51	0.99
5	Complex-5	18.11	33.06	0.83
6	Complex-6	20.36	39.84	0.78
7	Complex-1 (Solvothermal method)	27.79	27.16	1.93
8	Complex-3 (Solvothermal method)	22.31	27.86	1.36

The Cu and C contents in the complexes were determined by ICP method.

1.3 Determination of the Cu-Complex loading on Cu substrate

The loading of the complexes on the Cu substrate was determined by gravimetric method, which was obtained with an electrodeposition time of 1 h. The results are given in Supplementary Table 3.

Supplementary Table 3. Cu-Complex loading over different electrodes.

Entry	Sample	Loading (mg/cm^2)
1	Complex-1	0.67
2	Complex-2	1.44
3	Complex-3	1.57
4	Complex-4	1.75
5	Complex-5	2.20
6	Complex-6	0.80
7	Complex-1 (Solvothermal method)	1.00
8	Complex-3 (Solvothermal method)	1.00

1.4 Characterization of the Cu-Complexes by SAXS technique

Supplementary Figure 11 shows the lnI(h) vs. ln(h) plots of the complexes obtained from the SAXS data. Surface fractal (D_s) existed in the complexes, indicating that the surface of the complexes was coarse.

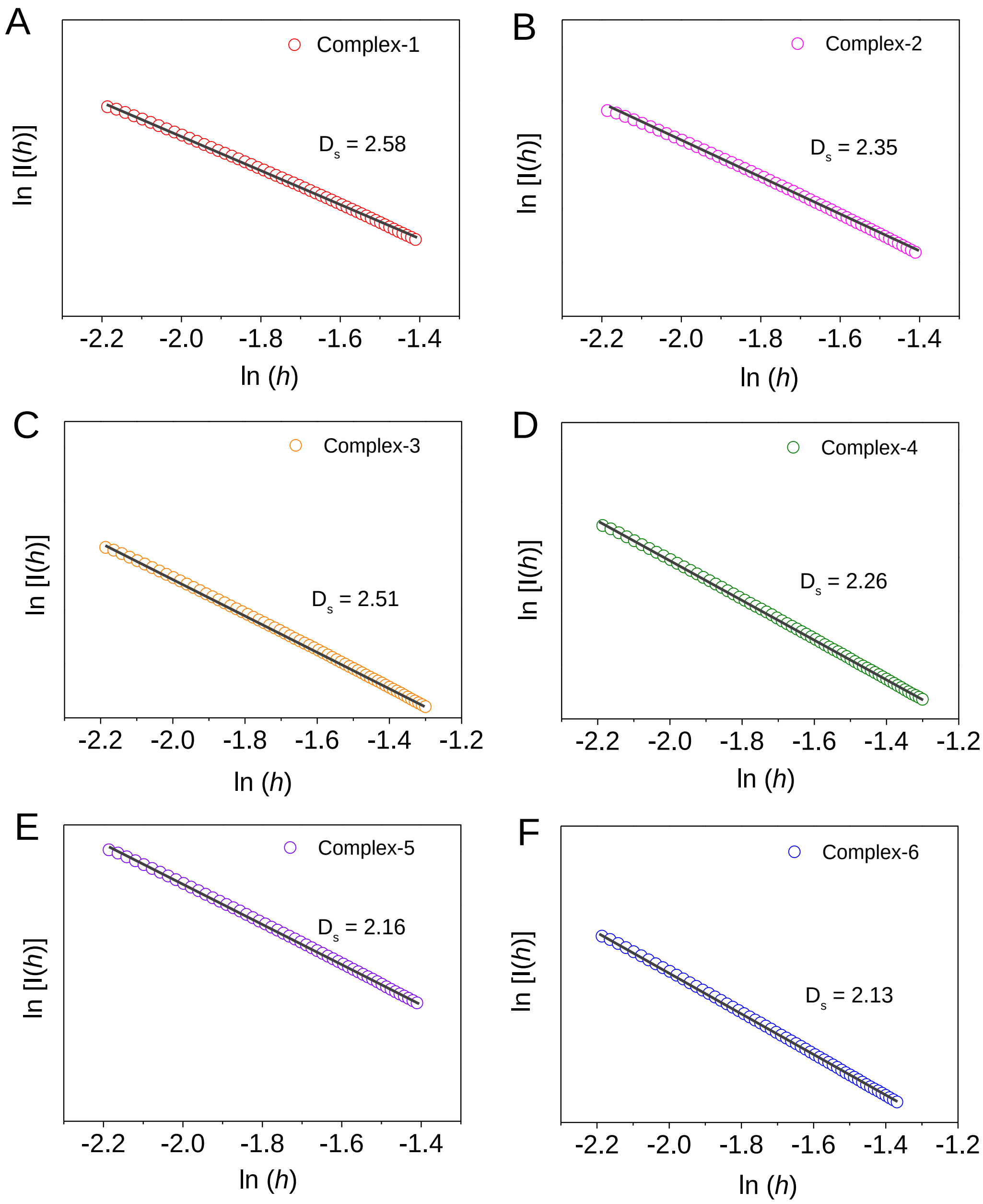

Supplementary Figure 11. lnI(*h*) vs. ln(*h*) plots of the complexes obtained from the SAXS data.

1.5 Electrochemical capacitance analysis

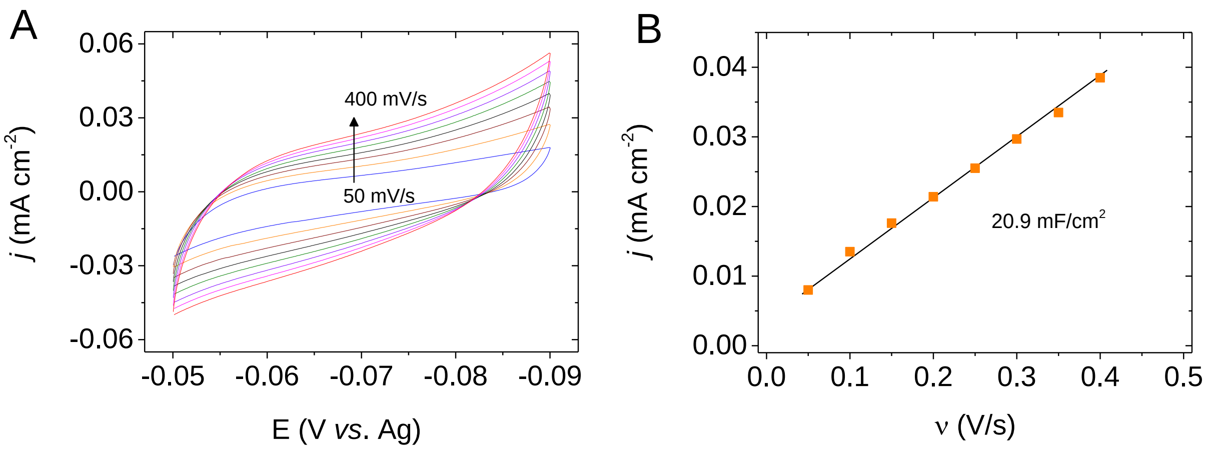

Supplementary Figure 12. (A) CV curves over Complex-1 at scan rate from 50 to 400 mV/s. Data obtained over a potential range of 40 mV around the open circuit potential in 0.1M $TBAPF_6$/AcN electrode. **(B) Plot of the current density due to double-layer charge/discharge against CV scan rate.**

Supplementary Table 4. Capacitance values and surface roughness factors of different electrodes. The surface roughness factor of the Cu foil is defined to be 1. The complex electrodes were prepared by electrodeposition at 9 V for 1 h.

Entry	Electrode	Capacitance	Surface Roughness Factor
1	Complex-1	20.9 mF	597
2	Complex-2	15.0 mF	429
3	Complex-3	15.9 mF	455
4	Complex-4	12.6 mF	360
5	Complex-5	12.4 mF	354
6	Complex-6	10.6 mF	302
7	Cu foil	35.0 μF	1

1.6 Crystal characterization of the Cu-Complexes

Analysis of the single crystals of the resulting complex powers generated by the electrosynthesis method yield a framework which is depicted in Supplementary Fig. 13-18. The empirical formula and relative molecular mass (M_r) of each complex is: Complex-1 ($C_{20}H_{44}Cu_4O_{36}$ (1114.71)), Complex-2 ($C_{18}H_{18}Cu_2O_{17}$ (633.40))[3], Complex-3 ($C_8H_{12}Cu_3O_{15}$ (658.9))[1,7], Complex-4 ($C_{18}H_{22}CuO_{18}$ (589.89)), Complex-5 ($C_{14}H_{14}CuN_2O_{11}$ (449.81)), and Complex-6 ($C_8H_4CuO_4$ (227.66))[6].

Crystal data for Complex-1, Complex-4 and Complex-5 are illustrated in Supplementary Table 15-21. Analysis of the single crystal data indicates that uncoordinated oxygen atom existing in the complex, suggesting that it is a protonated carbonxyl oxygen atom. This result is consistent with the observation of IR spectrum in which the presence of characteristic band at around 1699 cm^{-1} can be attributed to the protonized carbonxylic group (Supplementary Fig. 7). These ligands grow in pairs in the cavity and perpendicular to each other. They not only act as the connection units to construct two-dimensional layer structure, but also serve as arms protruding both sides of the sheet. The multipoint hydrogen bonding links, existed among lattice water molecules, coordinated aqua ligands and carboxyl groups, further extent the two-dimensional layer into a 3D supramolecular network. Obviously, the π-π stacking interactions between the aromatic rings also play an important role in stabilizing the whole crystal structure[2-7]. The most intriguing feature of the complexes is that the ligands can be assembled around metal centers in diverse arrangement. Such a unique arrangement manner generates a driving force that prompt the edge of a pore to deviate from one plane, thus leading to the formation of the helical shaped channel.

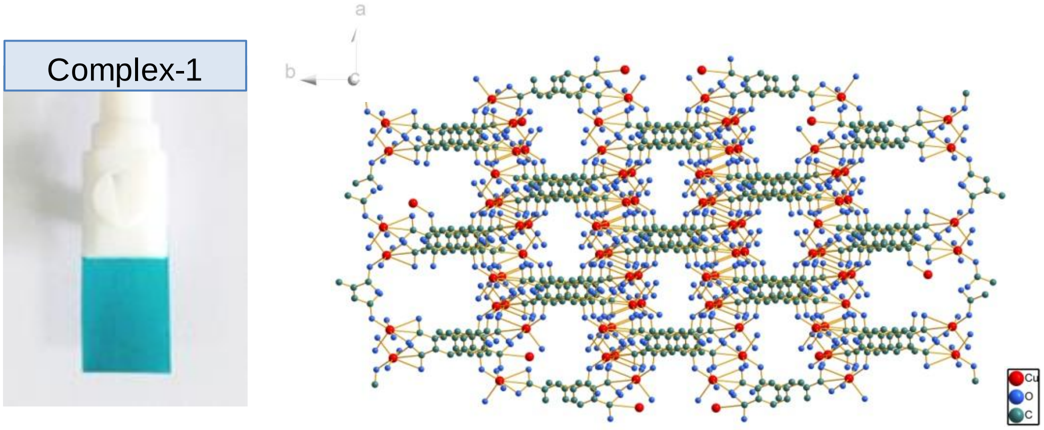

Supplementary Figure 13. Photograph and crystal structure of the Complex-1.

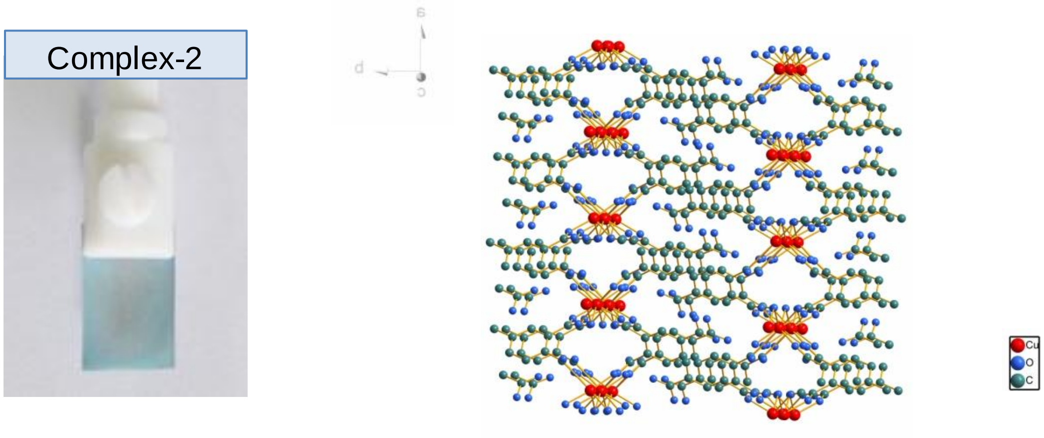

Supplementary Figure 14. Photograph and crystal structure of the Complex-2.
CCDC No: 230299 [DOI: 10.5517/cc7qn0l][3]

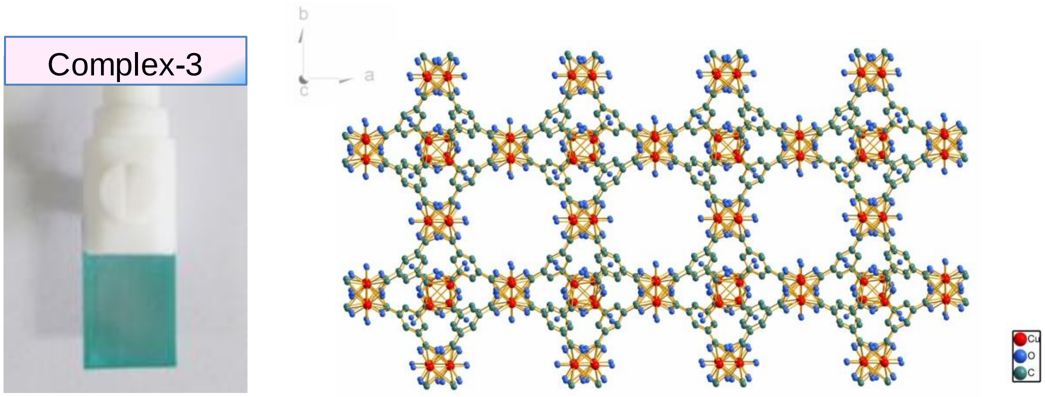

Supplementary Figure 15. Photograph and crystal structure of the Complex-3.
CCDC No: 112954 [DOI: 10.5517/cc3sjp2][7]

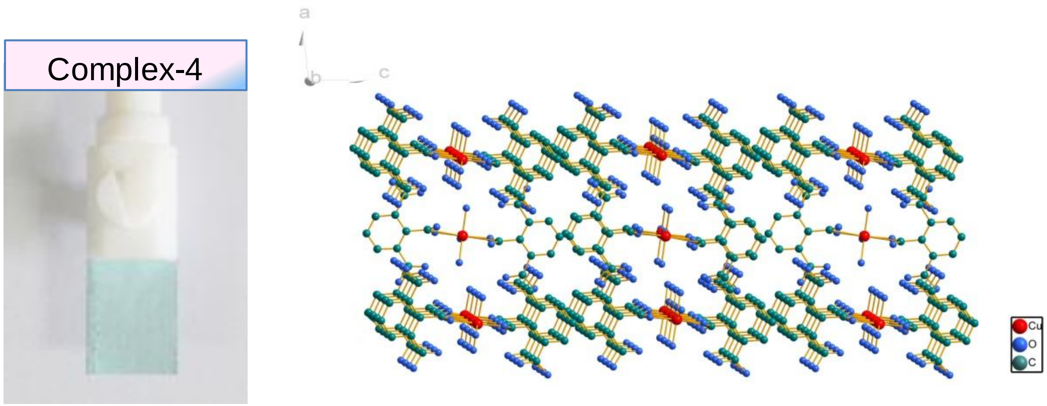

Supplementary Figure 16. Photograph and crystal structure of the Complex-4.

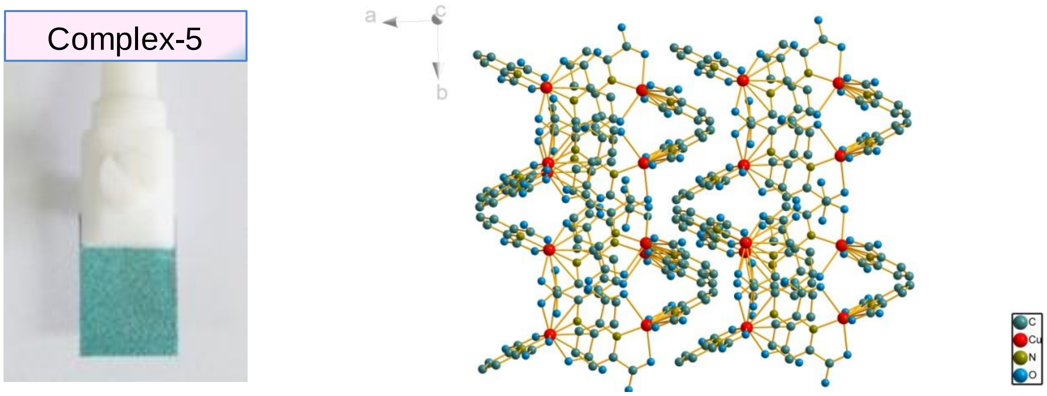

Supplementary Figure 17. Photograph and crystal structure of the Complex-5.

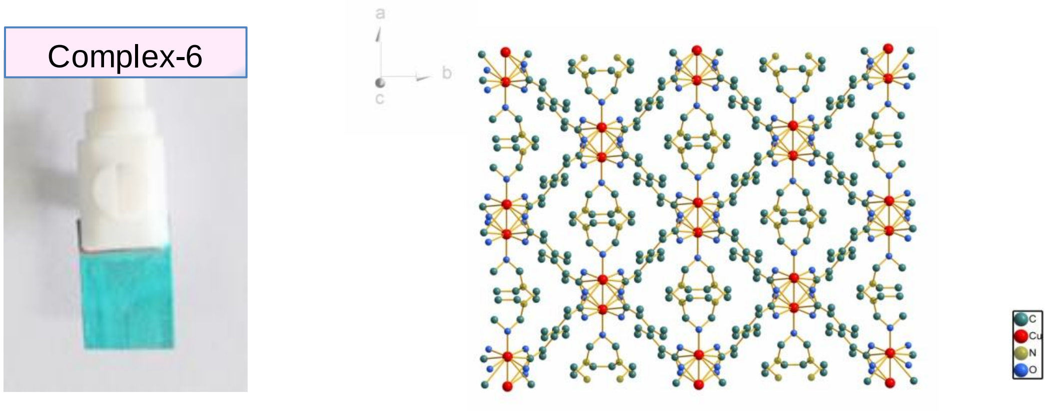

Supplementary Figure 18. Photograph and crystal structure of the Complex-6.
CCDC No: 687690 [DOI:10.5517/ccr2lky][6]

Supplementary note 2. In situ formation and characterization of Cu-Cu_2O catalysts via electroreduction of the Cu-Complexes on the Cu substrate

We found the Cu-Complexes can undergo in situ reduction during the CO_2 reduction reaction. Thus, the original Cu-complex species change to diffraction peaks of Cu_2O and Cu, respectively. The ligand is versatile for the fabrication of Cu complexes, which have different morphologies, lattice parameters, and spatial structures. Therefore, the growing interconnected Cu and Cu_2O grains from the constrained environment of the precursor, the obtained dendritic Cu_2O/Cu catalytic electrode surfaces were different apparently in morphologies, electrochemical active areas and the ratio of Cu_2O and Cu on the surface of the catalysts. All of them resulted in the dendritic structure with quantity of the active sites. The structural changes are known from XRD and SEM characterizations (Supplementary Figs. 19 and 20). Concomitantly, The SEM images show dendritic structures with a layer thickness of 20~50 μm which mainly consist of cuprite and copper.

In order to get more information about the morphology of the catalysts and to characterize the phases, high-resolution TEM measurements were performed on Cu-Cu_2O-1 catalyst after electroreduction of Complex-1 for 5h. The HR-TEM image in Fig. 2g shows a highly crystalline core with a disordered layer, the lattice spacings of the nanoparticle corresponding to the (111) spacing of Cu_2O (2.47 Å) and the (111) spacing of Cu (2.09 Å). The oxygen atoms were seen to be aligned between the layers in the interstitial tetrahedral sites. The lattice disorder on the surface suggests the existence of oxygen vacancies in the crystal structure. Moreover, the two spacings are seen to be directly related to each other, which indicate high density grain boundary between Cu and Cu_2O. Therefore, ionic transport properties could be improved by creating a high density of grain boundaries that act as channels to allow ions to enter the particles[9-11]. The relationship between the Cu_2O/Cu has a {111}//{111} crystallographic relationship across all particles. The Cu_2O {111}// Cu {111} relationship between the two phases would further reduce the strain between the mismatched phases causing the stable Cu_2O {111}//Cu {111} relationship to exist.

2.1 SEM images of various Cu-Cu_2O electrodes

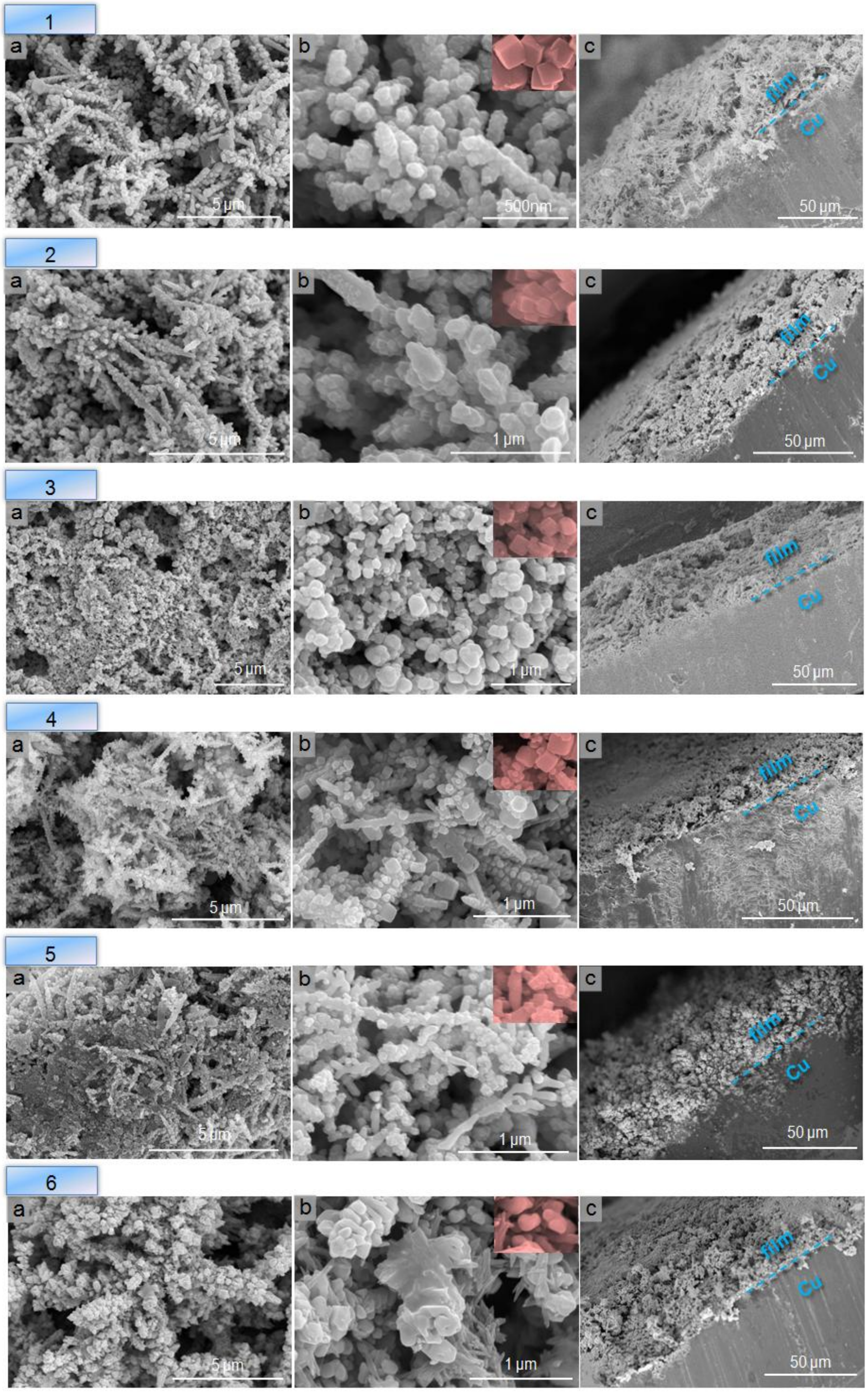

Supplementary Figure 19. SEM images of various Cu-Cu_2O/Cu electrodes after CO_2 electroreduction for 5 h. (a, b) top-view and (c) side-view.

2.2 Characterizations of various Cu-Cu_2O electrodes

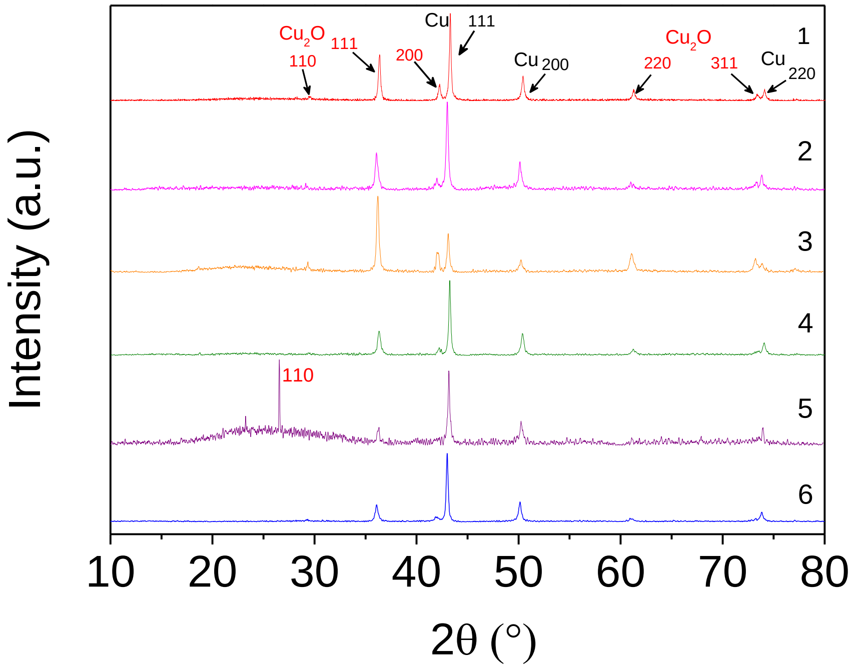

Supplementary Figure 20. XRD patterns of various Cu-Cu_2O electrocatalysts after CO_2 reduction.

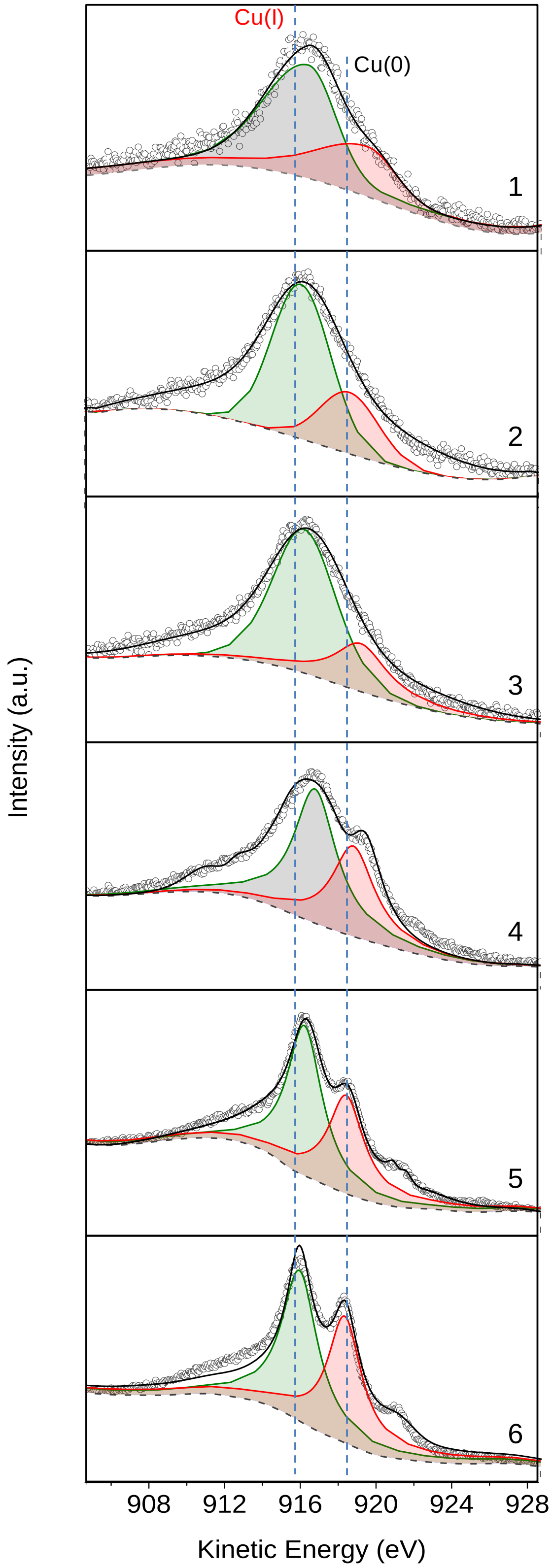

Supplementary Figure 21. Comparison of Cu-oxidation state from semi in-situ Cu Auger spectra after electroreduction for 5 h. The result shows that both Cu^{I} and Cu^{0} are present in the reduced and used catalysts after CO_2 reduction, as indicated by the

lower binding energy of Cu $2P^{3/2}$ than that of CuO, the disappearance of Cu 2P satellite peak at 942-944 eV, and the presence of two peaks at kinetic energies of about 916.5 and 914.4 eV in the Cu LMM spectra. The Cu LMM XAES characterization results of 914.4 eV with respect to Cu^I in the Cu_2O of Cu-O-Cu structure, while those reduced at 916.5 are donated by Cu^0 sites[12]. The XPS result reveal that Cu^I/Cu^0 species dominate the surface of the catalysts after electrolysis. Supplementary Table 5 show that the Cu^I to Cu^0 molar ratios of Cu-Cu_2O-1 to Cu-Cu_2O-6 electrode after reaction of 5 h. It indicates the proportion ratio of Cu^I/Cu^0 varies with the ligand of the respected Cu-Complexes.

Supplementary Table 5. Comparison of relative ratios of Cu(I) to Cu(0) in various electrocatalysts after CO_2 reduction.

Entry	Sample	Relative ratio of Cu(I) to Cu(0) during electrolysis
1	Cu-Cu_2O-1	1.17
2	Cu-Cu_2O-2	1.25
3	Cu-Cu_2O-3	1.31
4	Cu-Cu_2O-4	0.74
5	Cu-Cu_2O-5	0.66
6	Cu-Cu_2O-6	0.56

2.3 XAFS analysis

XAFS technique was used to investigate the change in the chemical states of copper species in the catalysts prepared using Complex-1 as the precursor during electrolysis. In this experiment, XAFS was applied to monitor the dynamic evolution of Cu species in the induction period of CO_2 reduction. Supplementary Fig. 22 shows the normalized O L-edge and Cu K-edge XAFS spectra and its first derivative profiles of the selected complex and reference catalysts. In situ Cu K-edge XAFS spectroscopy results show that the energy of adsorption edge (E^0) of Cu species increases with increasing oxidation degree. The E^0 at 8979.1, 8980.9 and 8984.3 eV are characteristic of Cu foil, Cu_2O and CuO reference electrodes respectively. A drastic shrink occurred to the Cu^I at 8981.0 eV and Cu^0 peak at 8979.1eV in the first derivative curve after the reduction. Supplementary Fig. 23 shows that the near-edge oscillation of the complex quickly changed to the pattern similar to that of Cu_2O after electrolysis.

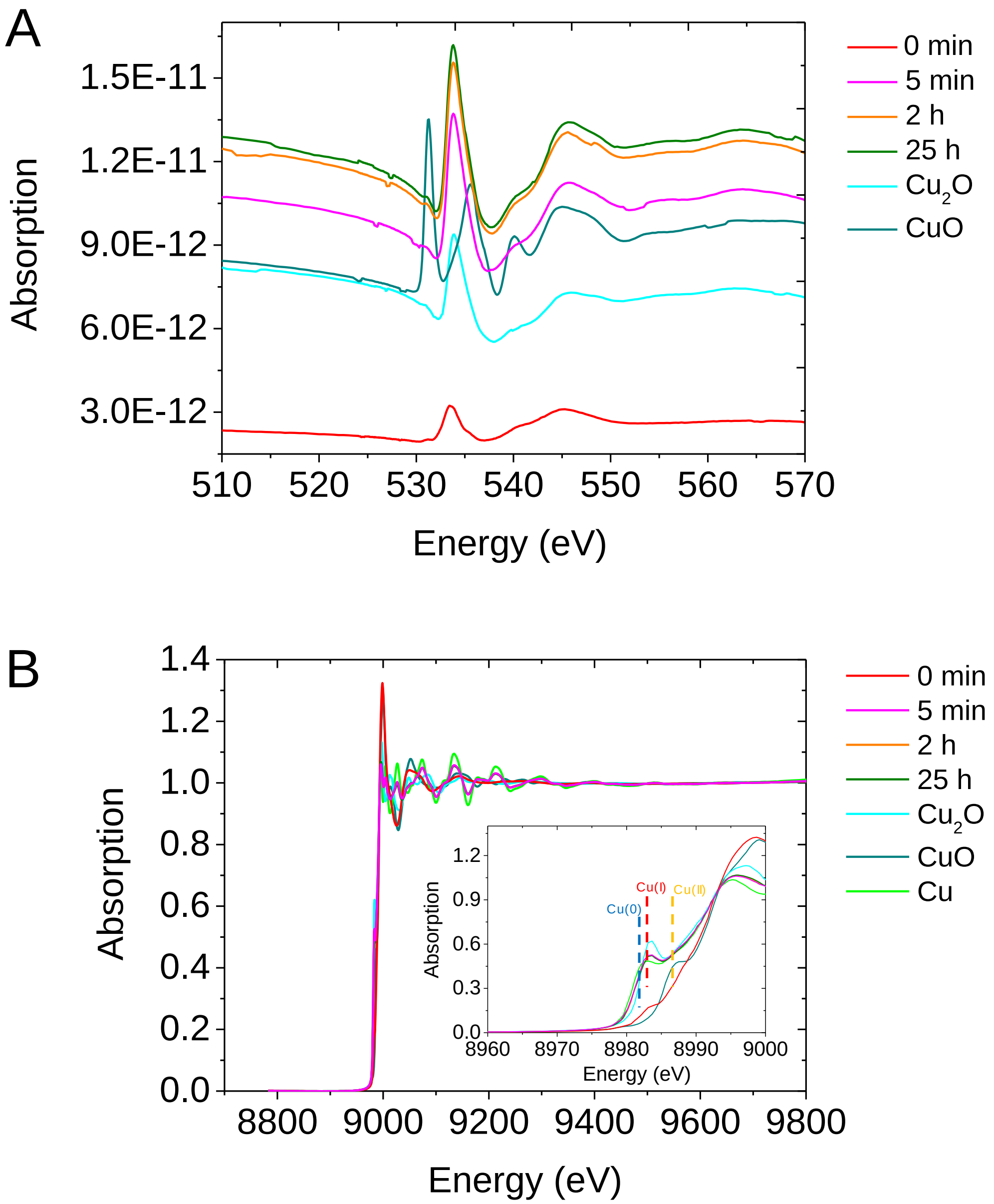

Supplementary Figure 22. The O L-edge **(A)** and Cu K-edge **(B)** EXAFS curve for Cu-Cu_2O-1 electrocatalysts during the CO_2 reduction.

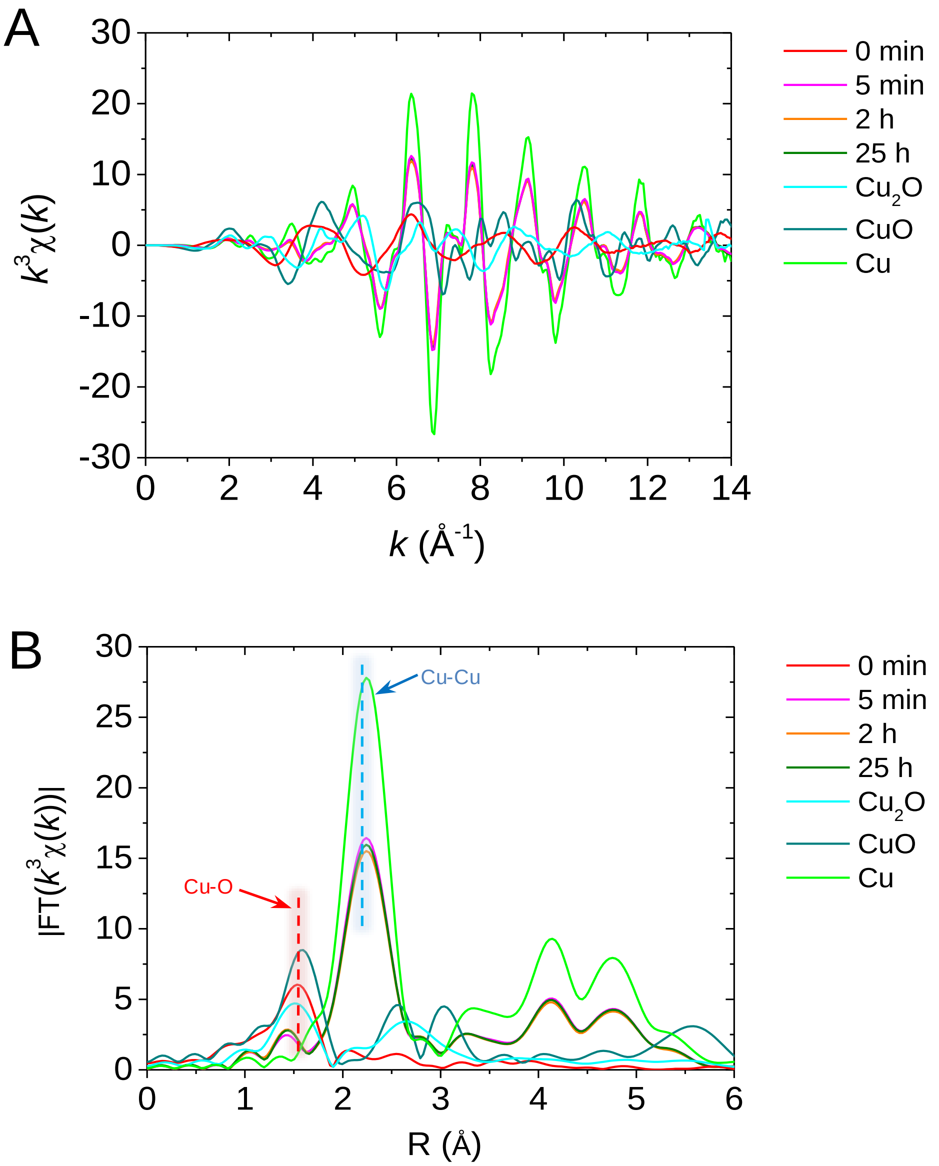

Supplementary Figure 23. Synchrotron radiation XAFS measurements. (A) Cu K-edge extended XAFS oscillation function $k^3\chi(k)$; **(B)** the corresponding Fourier transforms FT($k^3\chi(k)$) for Cu-Cu_2O-1 electrocatalysts during the CO_2 reduction.

Supplementary Table 6. The Cu K-edge EXAFS curve-fitting results for Cu-Cu_2O-1 electrocatalyst during the CO_2 reduction.

Sample	Path	R(Å)[a]	σ^2($Å^2$)[b]	Cu(I)/Cu(0) fraction
Cu-Cu_2O-1 (0 min)	Cu-O	1.534 $R_{Cu\text{-}O}$	0.005	-
Cu-Cu_2O-1 (5 min)	Cu-Cu<br>Cu-O	2.240 $R_{Cu\text{-}Cu}$<br>1.411 $R_{Cu\text{-}O}$	0.006	Cu(I)-56.6%<br>Cu(0)-43.4%
Cu-Cu_2O-1 (2 h)	Cu-Cu<br>Cu-O	2.240 $R_{Cu\text{-}Cu}$<br>1.441 $R_{Cu\text{-}O}$	0.006	Cu(I)-56.4%<br>Cu(0)-43.6%
Cu-Cu_2O-1 (25h)	Cu-Cu<br>Cu-O	2.240 $R_{Cu\text{-}Cu}$<br>1.442 $R_{Cu\text{-}O}$	0.006	Cu(I)-55.5%<br>Cu(0)-44.5%
Cu_2O	Cu-O	2.148 $R_{Cu\text{-}Cu}$	0.007	-
CuO	Cu-O	1.565 $R_{Cu\text{-}O}$	0.008	-
Cu	Cu-Cu	2.240 $R_{Cu\text{-}Cu}$	0.006	-

[a] R: radial distance between two adjacent atoms; [b]$\sigma^{2:}$ Debye-Waller factors.

Supplementary note 3. Electrochemical reduction of CO_2 over Cu-Cu_2O-1/Cu to Cu-Cu_2O-6 electrodes

3.1 LSV profiles over various Cu-Cu_2O catalysts and neat Cu substrate

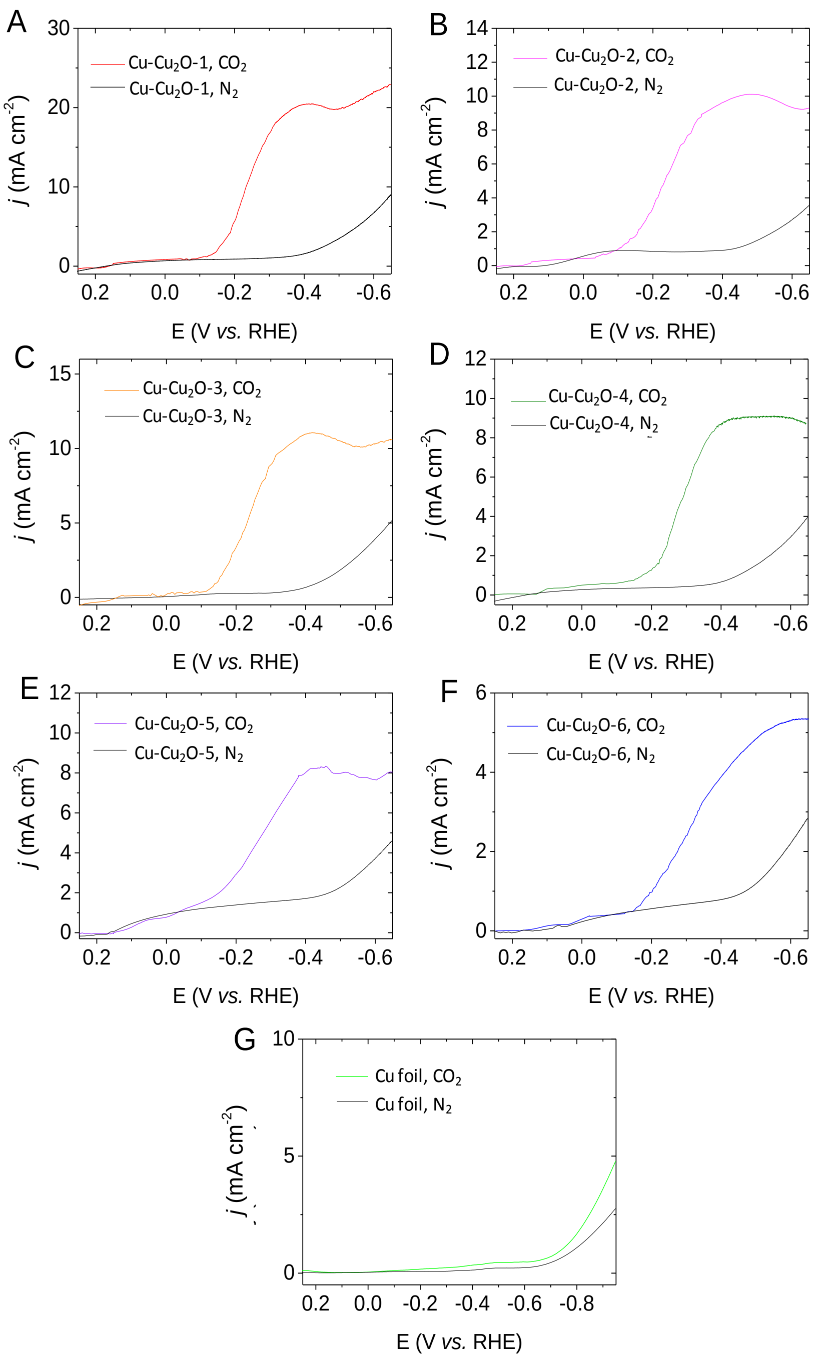

Supplementary Figure 24. LSV profiles over the Cu-Cu_2O electrode and neat Cu substrate in CO_2 and N_2 saturated 0.1M KCl aqueous solution. Scan rate: 20 mV/s.

3.2 Effect of electrolyte concentration

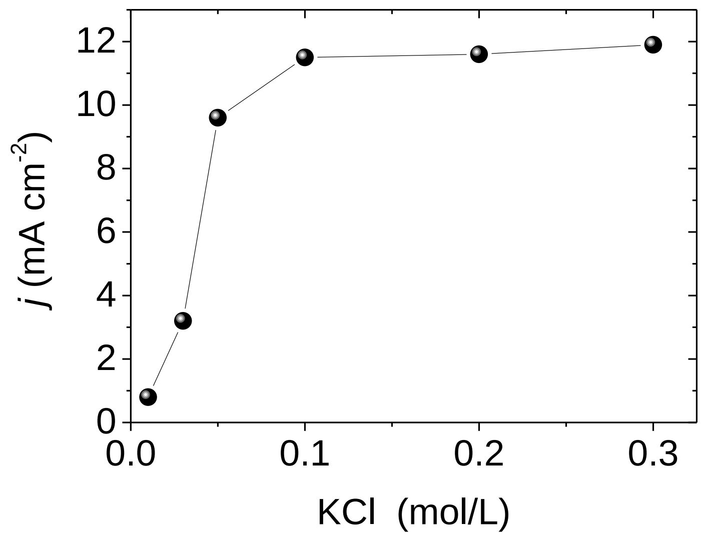

Supplementary Figure 25. Effect of concentration of KCl aqueous solution (electrolyte) on the current density of Cu-Cu_2O-1 electrode at -0.4 V vs RHE.

3.3 GC chromatographs of the gaseous products

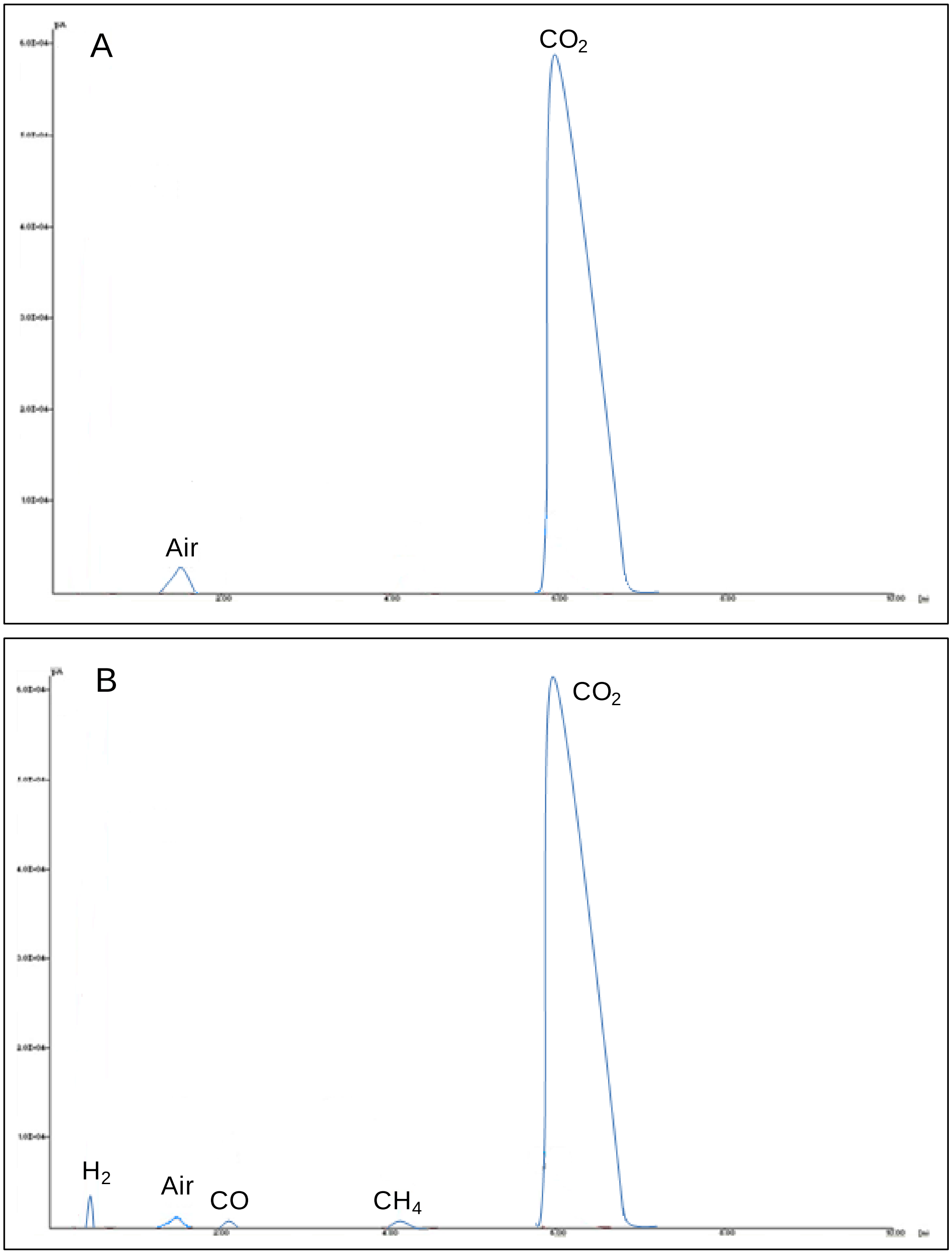

Supplementary Figure 26. Representative GC trace of the gaseous product using CO_2 as the feedstock (A) before electrolysis and (B) after electrolysis for 5 h at -0.4 V vs RHE over Cu-Cu_2O-1 electrode in 0.1 M KCl solution. The correction factors (*f*) for H_2: CO: CH_4 are 3.59: 3.63: 1.0.

3.4 Comparison study of the other Cu based electrodes

In this work, metallic Cu foam, Cu_2O and CuO were also used for CO_2 electrolysis. Therefore, purified metallic Cu was used as model Cu^0, Cu_2O was used as model Cu^I and Fig. 3 shows the catalytic results for CO_2 reduction over various reference electrodes at the reaction time of 5h. It is interesting that all the reference electrodes does not have significant effect on the catalytic activity for C_2 products, the applied potential is also far negative than the in-situ synthesized Cu-Cu_2O electrode. This strongly supports that synergetic effect occurs between Cu^I and Cu^0 species during the CO_2 reduction.

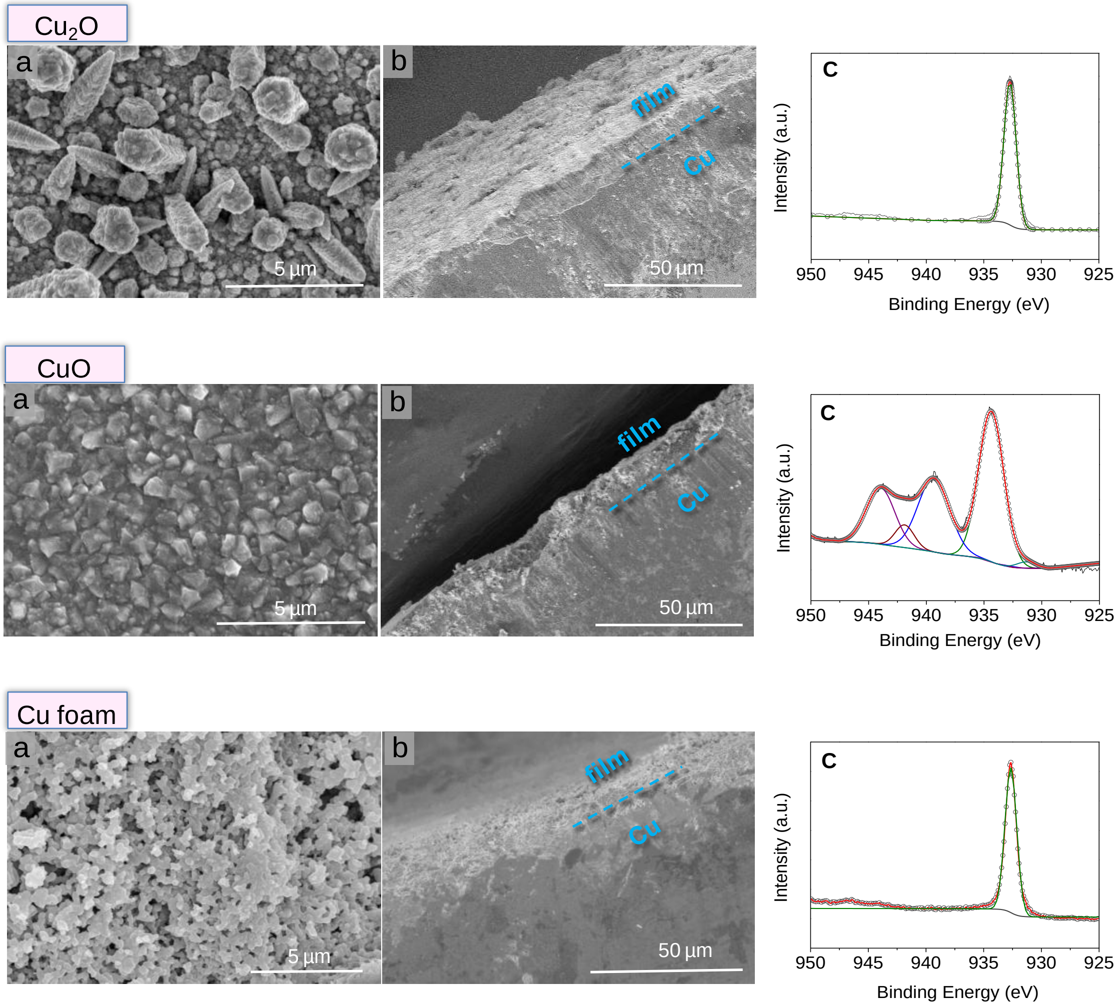

Supplementary Figure 27. SEM images and XPS spectra of various Cu-based catalysts. (a) top-view and (b) side-view SEM images of electrodeposited Cu_2O, CuO and Cu foam (directly electrodeposited without using the ligand) on Cu foil; **(c)** Cu 2p XPS spectra of each catalysts.

3.5 Product distribution over $Cu-Cu_2O$ electrodes

Supplementary Table 7. Faradaic efficiencies and current densities for electrocatalytic reduction of CO_2 over various electrodes in 0.1 M KCl aqueous electrolyte for 5 hrs at -0.4 V vs RHE.

Entry	Electrode	j_{tot} (mA cm^{-2})	$FE_{Acetic\ acid}$ (%)	$FE_{Ethanol}$ (%)	FE_{CO} (%)	FE_{CH4} (%)	FE_{H2} (%)	FE_{C1}[a] (%)	FE_{C2}[b] (%)
1	$Cu-Cu_2O$-1	11.5±0.3	48.5±2.3	32.2±1.5	1.5±0.2	9.6±1.5	6.8±0.3	11.1±1.7	80.7±3.8
2	$Cu-Cu_2O$-2	8.1±0.2	39.6±2.5	28.0±1.3	9.7±0.6	13.1±2.0	8.0±0.5	22.8±2.6	67.6±3.8
3	$Cu-Cu_2O$-3	7.3±0.2	17.5±1.3	39.2±2.0	13.8±0.8	14.5±2.3	11.8±1.3	28.3±3.1	56.7±3.3
4	$Cu-Cu_2O$-4	7.4±0.3	31.9±2.0	26.5±2.1	11.7±1.0	13.0±2.0	15.2±1.5	24.7±2.1	54.4±4.1
5	$Cu-Cu_2O$-5	5.1±0.3	27.2±2.1	23.0±1.5	13.2±1.3	3.1±0.5	23.0±2.0	16.3±1.8	50.2±3.6
6	$Cu-Cu_2O$-6	2.8±0.1	25.5±1.8	16.3±1.0	9.9±1.0	7.6±0.8	32.5±2.5	17.5±1.8	41.8±2.8

[a] summa of C_1 products;
[b] summa of C_2 products.

3.6 Overview of Cu based and other catalysts for reduction of CO_2

Supplementary Table 8. Overview of Cu based and other catalysts for reduction of CO_2 to C_1 and C_2 products.

Electrocatalyst	Potential (V vs RHE)	η (V)	Electrolyte	Major product	FE[§] (%)	*j** (mA cm^{-2})	Reference
**Overview of Cu and oxide derived Cu based electrocatalyst to C_1 products**							
Polycrystalline Cu	-1.17	N.A.[#]	0.1M $KHCO_3$	CH_4	40	17	13
Cu nanowire	−0.4	0.3	0.1M $KHCO_3$	CO	61.8	1.0	14
Cu foam	-0.45	~0.34	0.1M $KHCO_3$	CO<br>HCOOH	39<br>23	9.4	15
Cu nanofoams	-1.5V vs Ag/AgCl	N.A.	0.1 M $KHCO_3$	HCOOH	37	10	16
Porous hollow fibre Cu	-0.4	0.2-0.4	0.3 M $KHCO_3$	CO	85	~10	17
n-Cu/CP[a]	-1.8V vs Ag/AgCl	N.A.	0.1 M $KHCO_3$	CH_4	12.1	0.7	18
n-Cu/C[b]	-1.25	N.A.	0.1M $NaHCO_3$	CH_4	80	~9	19
CNT/Cu[c]	−2.8 V vs Ag/AgCl	N.A.	0.1M $NaHCO_3$	CO, CH_4	N.A. [#]	56~60	20
Cu-rGO[d]	−0.4<br>(−1.0 V<br>vs. Ag/AgCl)	N.A.	0.1M $NaHCO_3$	CO<br>CH_4<br>Liquid products	21.7<br>8.6<br>46.2	~1	21
$Cu_{(X)}GO/GC$[e]	-1.3	N.A.	0.1M $NaHCO_3$	CH_4	43.6	~4.6	22
Sulfur-modified Cu	-0.8	0.76	0.1M $KHCO_3$	HCOOH	80	2.5	23
Cu_2O layer	-0.3 to -0.65	<0.4V	0.1M $NaHCO_3$	CO<br>HCOOH	45<br>33	0.4~5	24
Cu_xO/Cu	-1.1V vs SHE	N.A.	0.5 M $KHCO_3$	HCOOH	62.2	~4	25
Cu_xO180-2[f]	-1.25V vs SHE	N.A.	0.5 M $KHCO_3$	HCOOH	59.36	20.5	26
Cu/CuO core/shell	-1.78V vs Ag/AgCl	N.A.	1 M $KHCO_3$	CO<br>HCOOH	~22<br>~20	58	27
Cu/Cu_2O+cyclam	-2.0 V vs F_c^+/F_c		DMF/H_2O<br>(99.1, v/v)	HCOOH	88	~1.2	28
$Cu_{32}H_{20}L_{12}$[g]	-0.55	0.3	0.1M $KHCO_3$<br>and 0.4M KCl<br>(pH6.8)	HCOOH	>80	~25	29
**Overview of Cu based electrocatalyst to C_{2+} products**							
Polycrystalline Cu	-1.09	N.A.	0.1M $KHCO_3$<br>with 10 mM N-tolylpyridinium chloride	C_2H_4<br>Ethanol<br>1-propanol	40.7<br>30.5<br>10.0	1.04	30

Polycrystalline Cu	-1.41 V vs NHE<br>-1.44 V vs NHE	N.A.	0.1M $KHCO_3$<br>0.1M KCl	CH_4<br>C_2H_4<br>ethanol<br>C_2H_4	29.4<br>30.1<br>21.9<br>47.8	5	31
Polycrystalline Cu	-1.05	N.A.	0.1M $KHCO_3$	ethanol<br>C_2H_4	9.75<br>25.98	~7	32
Cu (100)<br>Cu (S)-[4(100)X(111)]	-	-	0.1M $KHCO_3$	CH_4<br>C_2H_4<br>C_2H_4	30.4<br>40.4<br>50	5	33
Cu (111)<br>Cu (100)	-1.1<br>-0.97	N.A.	0.1M $KHCO_3$	CH_4<br>C_2H_4<br>CH_4<br>C_2H_4	42<br>16<br>39<br>8	N.A.	34
Plasma-activated Cu foils	-0.91	N.A.	0.1M $KHCO_3$	C_2H_4	60	~3 (par*)	35
Electrochemically cycled Cu	-1.0	N.A.	0.1M $CsHCO_3$	C_2H_4, ethanol, n-propanol	70.6 (tot)[§]	13	36
Electrodeposited Cu	-1.9V vs Ag/AgCl	N.A.	0.1M $KHCO_3$	C_2H_4<br>C_2H_6	~11<br>~2	N.A.	37
Prim-shaped Cu	-1.1	N.A.	0.1M $KHCO_3$	C_2H_4	27.8	28.6	38
Cu nanoparticles (<15nm)	-1.1	N.A.	0.1 M $KHCO_3$	CO<br>CH_4<br>C_2H_4	~30<br>~15<br>~5	N.A	39
Cu NPs/GDE[h]	-0.8	<0.7V	1M KOH	ethanol, C_2H_4	46 (tot)	~200	40
Cu nanoparticles	-1.1	N.A.	0.1 M $KClO_4$	CO<br>C_2H_4	34<br>36	N.A.	41
Cu-NC10[i]<br>Cu-NC20[i]	-0.95<br>-0.85	N.A.	0.1M $KHCO_3$	C_2H_4<br>n-propanol<br>ethanol<br>HCOOH<br>n-propanol<br>C_2H_4	35.82<br>8.75<br>12.75<br>12.4<br>10.58<br>17.69	7.128 (par)<br>1.74(par)<br>2.533 (par)<br>1.754 (par)<br>1.497 (par)<br>2.504 (par)	42
Cu nanoflower	-1.6	N.A.	0.1M $KHCO_3$	CH_4<br>C_2H_4	37.6<br>2.7	N.A.	43
Cu Cube	-0.8to -1.0	N.A.	0.1 M $KHCO_3$	C_2H_4	N.A.	N.A.	44
Cu nanocubes	-1.1	N.A.	0.1 M $KHCO_3$	C_2H_4	41.1	~5.6	45
Plasma-activated Cu nanocube	-1.0	N.A.	0.1M $KHCO_3$	C_2H_4<br>ethanol<br>n-propanol	~45<br>~22<br>~9	N.A.	46
Nano dendritic Cu	-2.0V vs Ag/AgCl	N.A.	0.1M KBr	C_2H_4	57	170	47
Mesopore Cu	-1.7V vs NHE	N.A.	0.1M $KHCO_3$	C_2H_4<br>C_3H_6	38<br>46	5.7<br>6.0	48
Cu mesocrystals	−0.99	N.A.	0.1M $KHCO_3$	C_2H_4	27.2	~25	49
CuX (X=Cl, Br or I)	-2.4V vs Ag/AgCl	N.A.	3M KX (X=Cl, Br or I)	C_2H_4	60.5-79.5	N.A.	50
KF cycled Cu foil	-1.0	N.A.	4mM KX in 0.1 M $KHCO_3$	C_2H_4<br>ethanol	16.3<br>7.85	6.51	51
Cu-10[j]	-1.2	N.A.	0.1 M $KHCO_3$	CH_4	62.25	30.43	52

CuO-1[k]	-1.1			CH_4<br>C_2H_4	33.97<br>27.14	24.27	
CuO-10[k]	-0.95			C_2H_4<br>ethanol	33.79<br>14.22	31.50	
CuO-60[k]	-0.9			C_2H_4<br>ethanol	26.68<br>12.87	36.99	
ERD Cu	-1.0V vs RHE	-	0.1M $KHCO_3$	C_2H_4	36	161	53
Cu(B)	-1.1V vs RHE	-	0.1M KCl	C_2H_4<br>Ethanol	79	70	54
Cu_2O or Cu nanocubes	-0.75V vs RHE	-	0.25M $KHCO_3$	C_2H_4<br>Ethanol<br>n-propanol	60.5	68	55
Oxide derived Cu foam	−0.8	N. A.	0.5M $NaHCO_3$	C_2H_4<br>C_2H_6	20.0<br>35.0	~12	56
Electrodeposited Cu_2O	-1.1	N.A.	0.1M $KHCO_3$	C_2H_4	~33	~12 (par)	57
Cu_2O on Cu	-0.99	N.A.	0.1 M $KHCO_3$	ethanol<br>C_2H_4	16.37<br>34.26	35	58
Cu_2O derived Cu	-31.2 mA cm^{-2}	N.A.	0.1 M $KHCO_3$	ethanol<br>C_2H_4	11.8<br>42.6	3.7 (par)<br>13.3 (par)	59
CuO nanoparticles	-1.7 V vs SCE	N.A.	0.2 M KI	ethanol<br>n-propanol	36.1	N.A.	60
Cu_2O/C[l]	-1.82V vs Ag/AgCl	N.A.	0.5 M $KHCO_3$	C_2H_4	26	~8	61
Cu_2O-derived Cu	-0.98	N.A.	0.1M $KHCO_3$	C_2H_4	32.4	~1.3	62
Cl-Cu_2O-Cu[m]	-1.6	N. A.	0.1M KCl	C_2H_4<br>ethanol<br>n-propanol	22<br>24<br>8.7	~6	12
Electrodeposited dendritic Cu	-0.7V vs RHE	N. A.	0.5M $KHCO_3$	HCOOH<br>C_2H_4	49.2<br>34.3	0.49<br>3.87	63
Annealing dendritic Cu	-1.0V vs RHE			ethanol<br>n-propanil	24.8<br>(tot)	2.81	
Cu/CNS[n]	-1.2	1.284	0.1M $KHCO_3$	ethanol	63	~2	64
Carbons-supported Cu nanoparticles[o]	-2.0V vs Ag/AgCl	N.A.	0.1M $KHCO_3$	CH_4<br>C_2H_4	~30<br>~48	N.A.	65
Cu-C_3N_4	-1.6V vs Ag/AgCl	N. A.	0.1M $KHCO_3$	ethanol, C_2H_4,<br>C_2H_6	~10<br>(tot)	~7.5	66
Cu10-CNT[p]	-0.5V vs Ag/AgCl	N.A.	0.5M $KHCO_3$	acetic acid	56	N.A.	67
Pd-decorated Cu	-0.96	N.A.	0.5M $KHCO_3$	CH_4, C_2H_4	>50<br>(tot)	57	68
Cu_4Zn	-1.05	N.A.	0.1M $KHCO_3$	ethanol<br>C_2H_4	29.1<br>4.1	8.2	69
Cu-Au alloy	-1.1 V vs SCE	N.A.	0.5M $KHCO_3$	methanol<br>ethanol	28 (tot)	N.A.	70
CuAg Alloys	-1.05	N.A.	0.05M Cs_2CO_3	acetate<br>acetaldehyde	~15<br>(tot)	~0.6 (Par)	71
Cu_2O derived Cu with $PdCl_2$ added	-1.0	N.A.	0.1M $KHCO_3$	C_2H_6	30.1	~20	72
Cu_2O derived Cu				C_2H_4<br>ethanol	32.1<br>16.4	N.A.	
PYD@Cu-Pt[q]	-1.2V vs SCE	N.A.	0.5 M KCl	ethanol<br>n-propanol	24<br>1	25	73

Cu-DAT wire[r]	-0.5	N.A.	1M KOH	C_2H_4, ethanol	40<br>20	~60 (par)<br>~30 (par)	74
Cu-porphyrin complex[s]	-0.976	N.A.	0.5M $KHCO_3$	CH_4<br>C_2H_4	27<br>17	49	75
HKUST-1[t]	-0.9V vs Ag/AgCl	N. A.	0.5 M $KHCO_3$	ethanol	10.3	20	76
CuZnDTA GDEs[u]	-1.25V vs Ag/AgCl			ethanol	6.5	10	
Cu/THH Pd NCs[v]	-0.46	N. A.	0.1M $NaHCO_3$	ethanol	20.4	N.A	77
Cu NW+Gly[w]	-1.9V vs Ag/AgCl	N.A.	0.1M $KHCO_3$	C_2H_4<br>C_2H_6	34.1<br>(tot)	~3	78
Cu_1/BN-C_{30}[x]	-2.2V vs Ag/Ag^+	0.52	[Emim]BF_4-LI-H_2O[y]	acetic acid	80.3	13.9	79
Graphite/carbon NPs/Cu/PTFE GDE	-0.55V vs RHE	-	1M KOH	C_2H_4	70	110	80
Cu_2S-Cu-V	-0.95V vs RHE	-	0.1M $KHCO_3$	C_2H_4<br>Ethanol<br>n-propanol	32	126	81
**Overview of other electrocatalyst to C_{2+} products**							
Nitrogen-doped mesoporous carbon	-0.56	N.A.	0.1M $KHCO_3$	ethanol	77	~0.25	82
Pd-Au	-1.4	N. A.	0.1 M KH_2PO_4/0.1M K_2HPO_4	CH_4<br>C_2H_4	0.7<br>(tot)	N.A.	83
Nitrogen-doped nanodiamond	-0.8 to -1.0	~0.63	0.5M $NaHCO_3$	acetate	77.3~7<br>7.6	~0.75	84
Ni_5Ga_3	-1.18	N.A.	0.1M $KHCO_3$	C_2H_4<br>C_2H_6	~40<br>~8.5	~5 (par)<br>~1 (par)	85

[a]Cu nanoparticles on porous carbon paper; [b]Cu nanoparticles supported on glassy carbon carbon; [c]Carbon nanotube/Cu sheets; [d]Cu-reduced graphene oxide nanocomposite; [e]Cu nanoparticles on GO dispersed GC; [f]Cu_xO nanocatalysts synthesized at 180℃ for 2hs; [g]Precise ligand-protented Cu-hydride nanoclusters (L is a dithiophosphate ligand); [h]Cu nanoparticles on gas diffusion electrode; [i]Cu nanocrustals-10 and Cu nanocrustals-20; [j]metallic Cu films deposited for 10 min; [k]CuO films deposited for 1 min, 10 min and 60 min; [l]Electrodeposited Cu_2O on carbon; [m]Chloride-induced bi-phasic cuprous oxide and metallic Cu; [n]Carbon nanospike electrode with electronucleated Cu nanoparticles; [o]Carbons include, Carbon black (VC), single-wall carbon nanotubes (SWNTs), Ketjen black (KB); [p]copper nanoparticles on carbon nanotubes; [q]Pyridine derivative and Cu-Pt alloy composite; [r]DAT: 3,5-DIAMINO-1,2,4-triazole; [s]Cu (II)-5,10,15,20-tetrakis-(2,6-dihydroxyphenyl) porphyrin; [t]Cu-based metal organic porous materials; [u]bis-bidentate dithiooxamidate gas diffusion electrode; [v]Cu/ tetrahexahedral Pd nanocrystals; [w]glycine modified Cu nanowrie; [x]N-based Cu(I)/C-doped boron nitride; [y][Emim]BF_4: 1-ethyl-3-methyl-Imidazolium-Tetrafluoroborate; [#]not available; *partial current density; [§]total Faradaic efficiency.

3.7 Long-term stability of the Cu-Cu_2O electrodes in the electrolysis

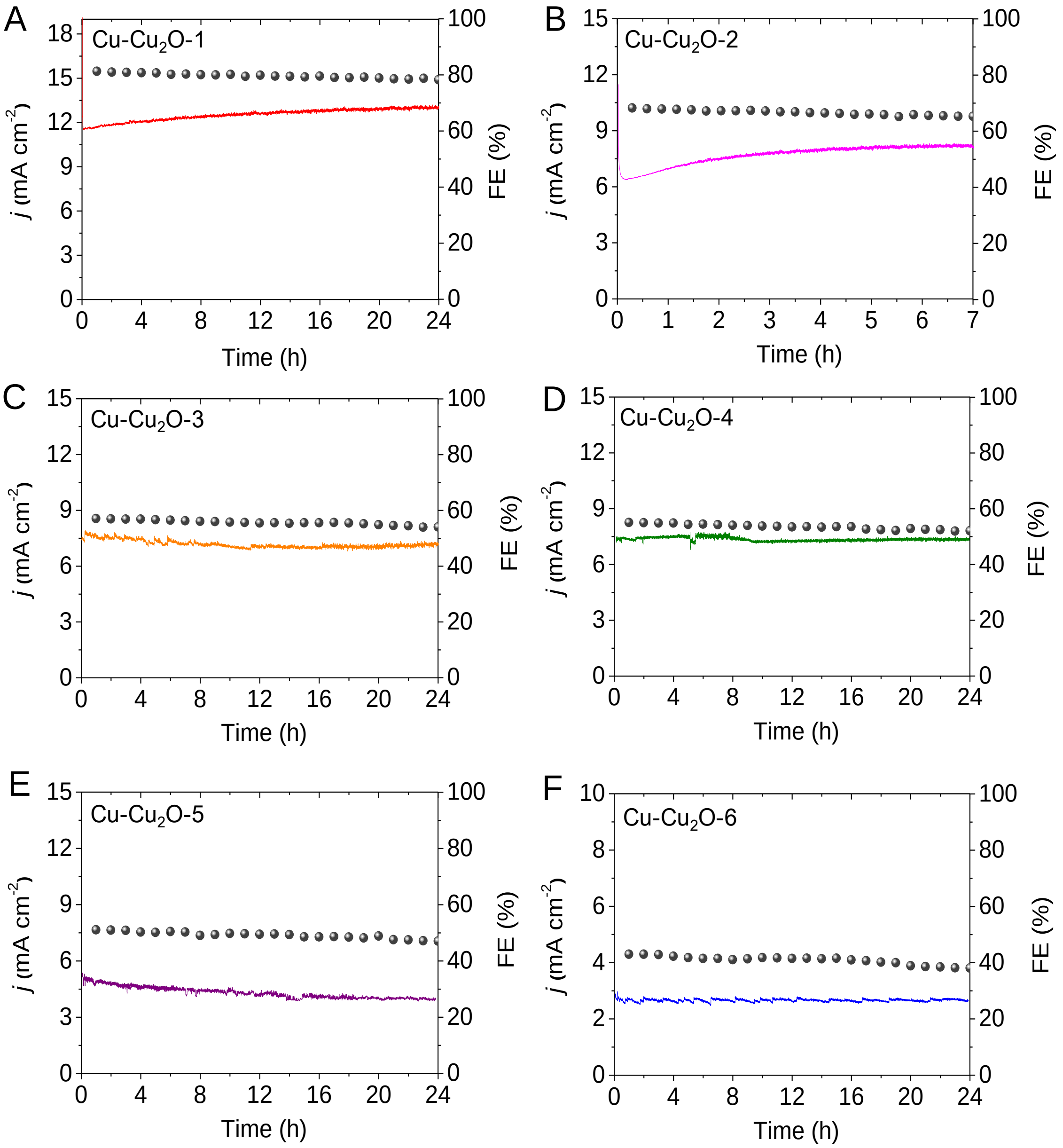

Supplementary Figure 28. Current density and Faradaic efficiency of C_2 products vs time over various Cu-Cu_2O/Cu electrodes at applied potential of -0.4 V vs RHE.

3.8 Production rate and TOF of C_2 products over Cu-Cu_2O electrodes

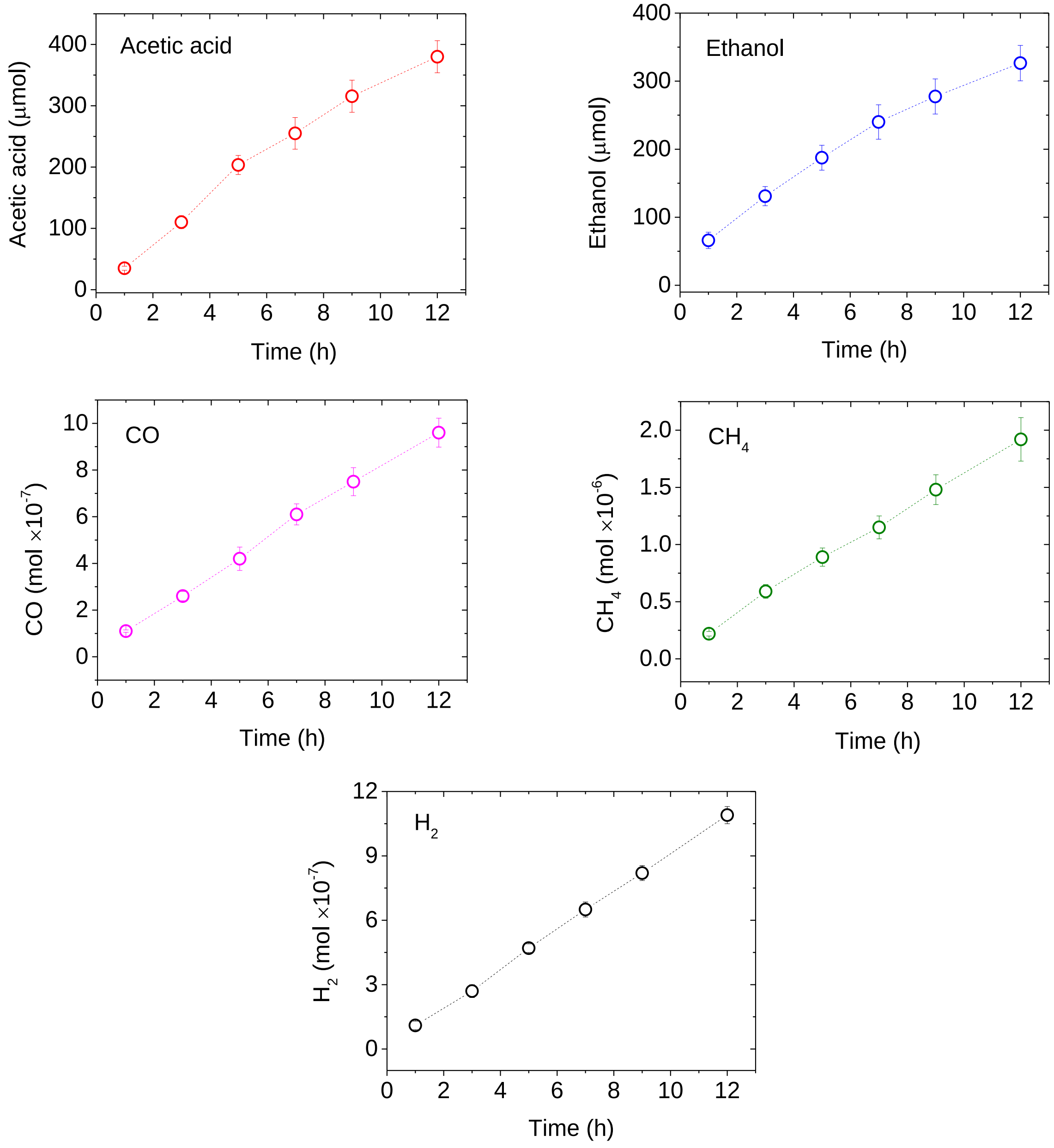

Supplementary Figure 29. The amounts of the liquid and gaseous products generated from CO_2 reduction at different times over Cu-Cu_2O-1/Cu electrode at -0.4 V *vs* RHE. The error bars represent the standard deviation of three measurements.

Supplementary Table 9. Production rate of liquid products over Cu-Cu_2O-1/Cu electrode in 0.1 M KCl aqueous electrolyte at different applied potentials.

Applied potential (V vs RHE)	Production rate[a]	
	Acetic acid	Ethanol
-0.25	2.1 ±0.2	1.2 ±0.1
-0.30	7.6 ±0.3	5.5 ±0.2
-0.35	12.8 ±0.4	11.0 ±0.2
-0.40	17.0 ±0.3	15.2 ±0.3
-0.45	19.5 ±0.4	17.1 ±0.3
-0.50	12.0 ±0.2	19.3 ±0.3
-0.55	6.2 ±0.3	19.6 ±0.5

[a] Production rate was calculated as moles of product (Acetate or Ethanol) per mole of Cu atom per hour after 1 h electrolysis.

Supplementary Table 10. TOF values of liquid products over various catalysts in 0.1 M KCl aqueous electrolyte at optimized potentials.

Catalysts	TOF (h^{-1})	
	Acetic acid	Ethanol
**CO_2 reduction at -0.4 V vs RHE for electrode prepared by electrodeposition method**		
$Cu\text{-}Cu_2O\text{-}1$	17.0±0.3	15.6±0.3
$Cu\text{-}Cu_2O\text{-}2$	4.8±0.2	4.6±0.1
$Cu\text{-}Cu_2O\text{-}3$	1.7±0.1	4.8±0.2
$Cu\text{-}Cu_2O\text{-}4$	1.1±0.1	1.1±0.1
$Cu\text{-}Cu_2O\text{-}5$	4.0±0.2	2.6±0.1
$Cu\text{-}Cu_2O\text{-}6$	2.2±02	2.1±0.1
**CO_2 reduction at -0.7 V vs RHE for electrode prepared by solvothermal method**		
$Cu\text{-}Cu_2O\text{-}1$ (Solvothermal method)	3.8±0.2	4.3±0.3
$Cu\text{-}Cu_2O\text{-}3$ (Solvothermal method)	1.4±0.1	4.1±0.2

[a] TOF was calculated as moles of product (Acetate or Ethanol) per mole of Cu atom per hour after 1 h electrolysis; TOF=TON h^{-1}.

3.9 Effects of electrolytes

We did electrolysis experiments using different electrolytes over Cu-Cu_2O-1/Cu electrode. Supplementary Fig. 30 illustrates the effect of the current density in KCl and $KHCO_3$ aqueous electrolytes. It is clear that the total current density is higher in KCl electrolyte, and this trend is observed at all applied potentials.

Supplementary Table 11 summarizes the selectivity of product for various Cu-Cu_2O electrodes in $KHCO_3$ electrolyte. Interestingly, the electrolysis experiments demonstrate that the product distribution in $KHCO_3$ aqueous solvent lead to production of 47.6% of ethanol as the major C_2 product and 32.4% of formic acid as the major C_1 product on Cu-Cu_2O-1 electrode. The most striking observation is that, for all of the catalysts, higher selectivity and partial current densities toward C_2 products can be attained using a KCl electrolyte compared to a $KHCO_3$ electrolyte at all applied potentials. In Supplementary Fig. 31, the total current density and FE of ethanol increase initially and then decline as the potential is negatively shifted, reaching the maximum FE at -0.4V vs RHE in 0.5M $KHCO_3$ aqueous solvent. The products distributions are also similar on other electrodes. These metrics are extremely noteworthy, as the results demonstrate that the changes of the catalytic activity of Cu-Cu_2O-1 electrode in different electrolytes have also been attributed to the different nature of the solvatized ions in solution. These electrolytes can control the conversion of CO_2 to different product with a level of efficiency and selectivity that is typically only accessible with precious metal catalysts that are both rare and expensive. Similarly, the thickness of the complex film was tuned to optimize the performance of the Cu-Complex by varying the deposition time from 5 min to 2h (Supplementary Fig. 32). The performance of the Cu-Cu_2O-1 catalyst in $KHCO_3$ aqueous solvent also increased with increasing active-site loading until reaching a maximum at deposition time of 1h. Therefore, we can conclude that adding of KCl as electrolyte affects the C_2 product selectivity.

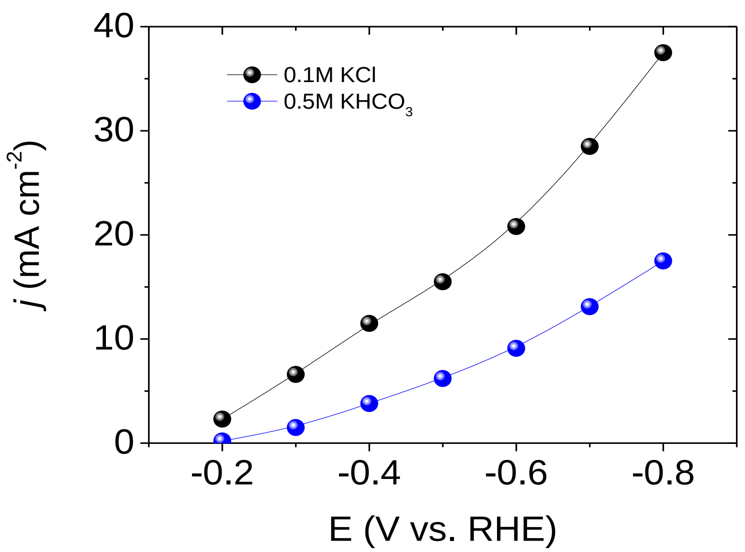

Supplementary Figure 30. Effect of cation and anion on the total activity over Cu-Cu_2O electrode. Scan rate: 20 mV/s.

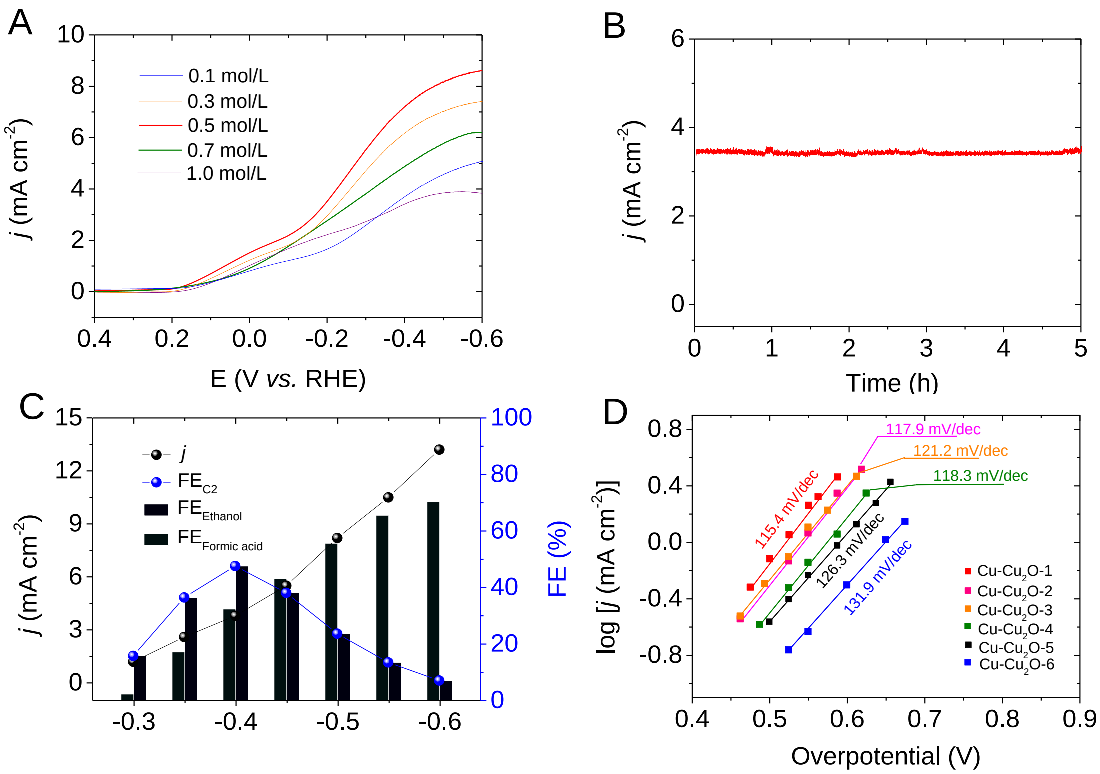

Supplementary Figure 31. Electroreduction of CO_2 over Cu-Cu_2O-1 electrode in $KHCO_3$ aqueous solution. (A) LSV curve of CO_2 reduction over Cu-Cu_2O-1 electrode in different concentration of $KHCO_3$ electrolyte; **(B)** The dependence of current density on time over Cu-Cu_2O-1electrodes; **(C)** Faradaic efficiencies and current densities of C_1

and C_2 products at various applied potential; **(D)** Dependence of partial current density of ethanol on overpotential. Electrolysis data were collected at room temperature and ambient pressure with electrolysis time of 5 h; 0.5 M $KHCO_3$; CO_2 stream, 5 sccm.

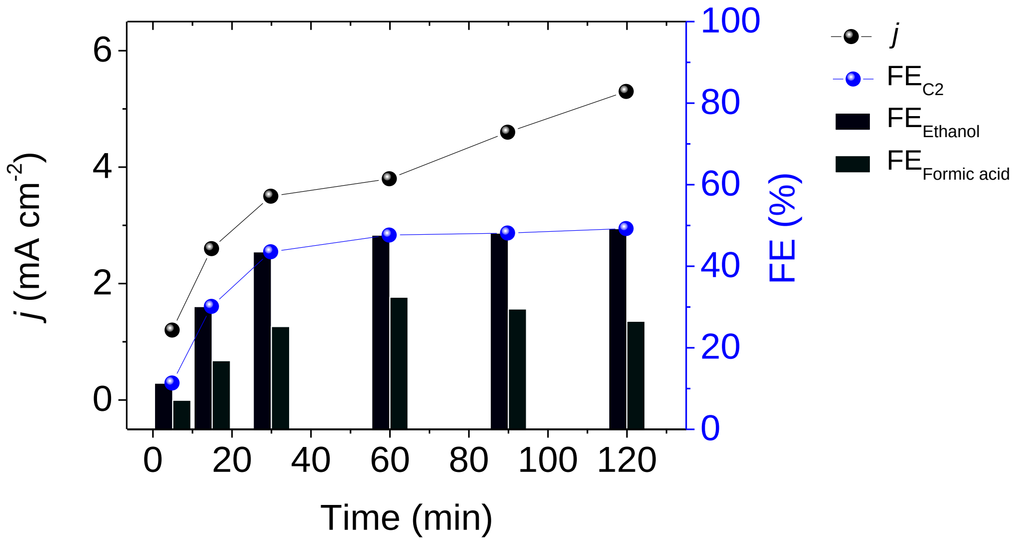

Supplementary Figure 32. Current densities and Faradaic efficiencies of major products in 0.5 M $KHCO_3$ electrolyte using Cu-Cu_2O-1 electrode with electrodeposition time from 5 min to 2 h.

Supplementary Table 11. Faradaic efficiencies and current densities for electrocatalytic reduction of CO_2 over various electrodes in 0.5M $KHCO_3$ aqueous electrolyte at an applied potential of -0.4 V vs RHE.

Entry	Electrode	j_{tot} (mA cm^{-2})	$FE_{Ethanol}$[a] (%)	$FE_{Formic\ acid}$ (%)	FE_{CO} (%)	FE_{CH4} (%)	FE_{H2} (%)	FE_{C1}[b] (%)	FE_{C2}[c] (%)
1	Cu-Cu_2O-1	3.8±0.3	47.6±3.5	32.4±1.2	2.3±0.1	7.2±0.3	10.5±2.3	41.9±1.6	47.6±3.5
2	Cu-Cu_2O-2	2.8±0.2	41.8±3.2	34.2±1.3	2.6±0.1	6.7±0.2	14.7±2.5	43.5±1.6	41.8±3.2
3	Cu-Cu_2O-3	2.83±0.2	45.2±2.8	22.5±0.6	2.8±0.2	9.7±0.4	19.8±3.0	35.0±1.2	45.2±2.8
4	Cu-Cu_2O-4	1.8±0.1	40.2±3.0	5.6±0.3	5.4±0.1	9.5±0.4	39.3±3.2	20.5±0.8	40.2±3.0
5	Cu-Cu_2O-5	1.26±0.1	47.1±4.0	-	4.0±0.3	8.8±0.3	40.1±3.0	12.8±0.6	47.1±4.0
6	Cu-Cu_2O-6	0.75±0.05	31.4±2.6	11.6±1.0	3.1±0.2	5.5±0.3	48.4±5.2	20.2±1.5	31.4±2.6

[a]ethanol. [b]summa of C_1 products. [c]summa of C_2 products.

3.10 $^{12}CO_2$ and $^{13}CO_2$ NMR characterization after electrolysis

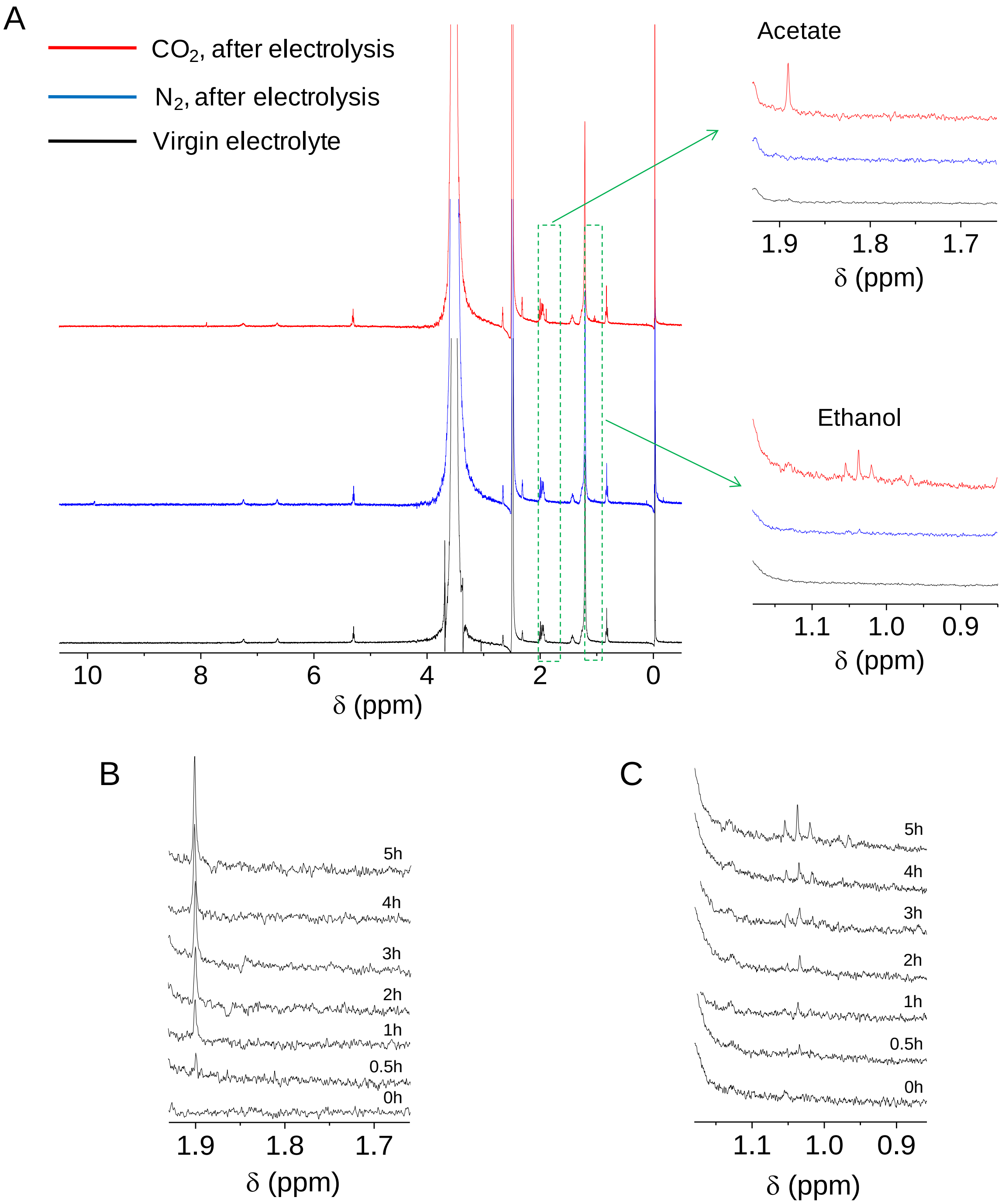

Supplementary Figure 33. Representative ^{1}H NMR spectra of (A) virgin electrolyte (0.1 M KCl aqueous solution), N_2-saturated electrolyte and CO_2 saturated electrolyte after an electrolysis time of 5 h at -0.4V vs RHE. (B) Acetate generated

from CO_2 reduction on Cu-Cu_2O-1 electrode over time at -0.4 V vs RHE. (C) Ethanol generated from CO_2 reduction on Cu-Cu_2O-1 electrode over time at -0.4V vs RHE.

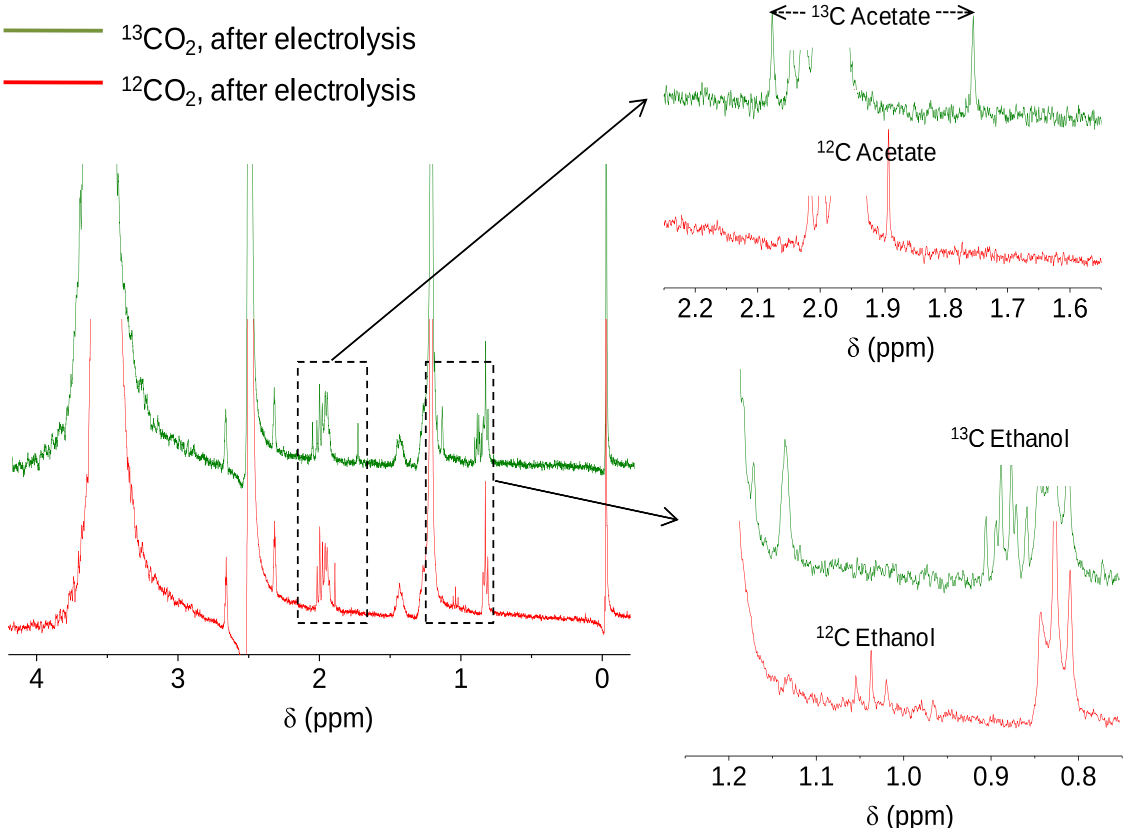

Supplementary Figure 34. ^{1}H NMR spectra of the electrolyte solutions after electrolysis using $^{12}CO_2$ and $^{13}CO_2$ as the feedstocks over Cu-Cu_2O-1 electrode in CO_2-saturated 0.1 M KCl solution at -0.4 V vs RHE with an electrolysis time of 3 h. The splitting of H peaks of acetate and ethanol by ^{13}C atom can be found in the ^{1}H NMR spectra. They distribute on the both sides of the chemical shift of H-^{12}C symmetrically. From the ^{1}H NMR spectra, we can only observe ^{13}C signal for acetate and ethanol, which indicate that the C_2 products were derived from CO_2 rather than other C-based chemicals in our reaction system.

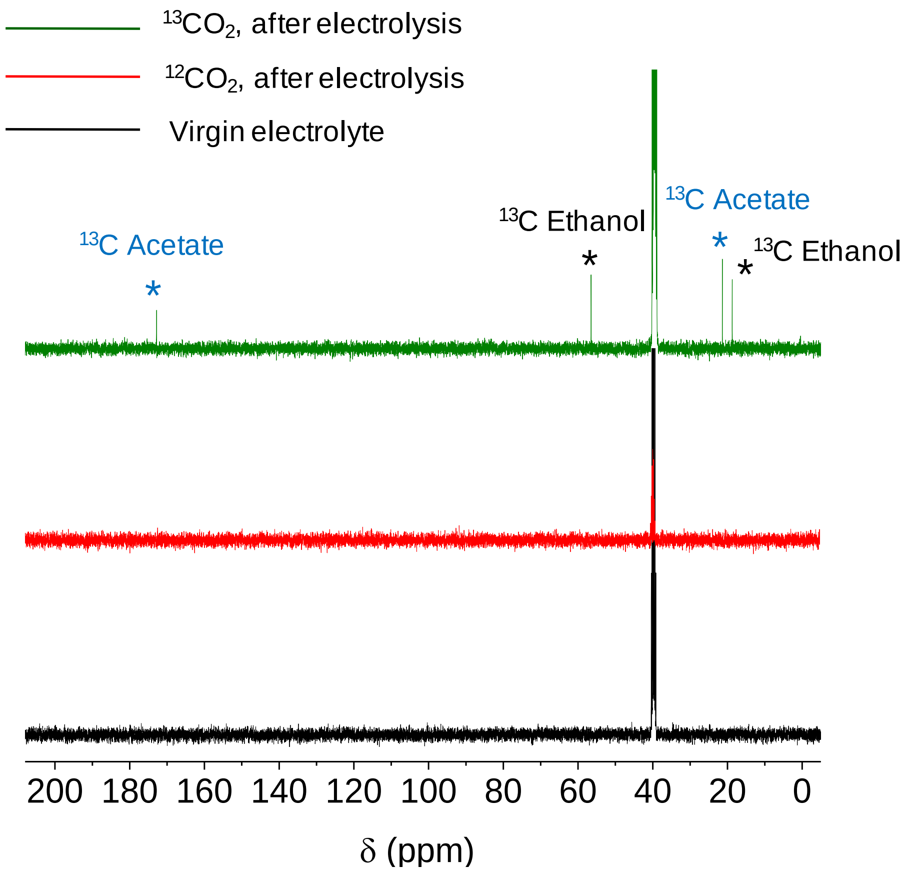

Supplementary Figure 35. ^{13}C NMR spectra of the electrolyte solutions after electrolysis using $^{12}CO_2$ and $^{13}CO_2$ as the feedstocks over Cu-Cu_2O-1 electrode in CO_2-saturated 0.1 M KCl solution at -0.4 V vs RHE with an electrolysis time of 3 h. The spectrum of the virgin electrolyte is also given for comparison. No C signal attributed to acetate or ethanol was detected when we used $^{12}CO_2$ as the feedstock. But strong C signal attributed to acetate and ethanol can be found when $^{13}CO_2$ was used. The results also indicate that all the carbon atoms in the product were from CO_2.

Supplementary note 4. Performance of Cu-Cu_2O electrodes prepared by different methods

4.1 Characterization of Cu-Complexes prepared by solvothermal method

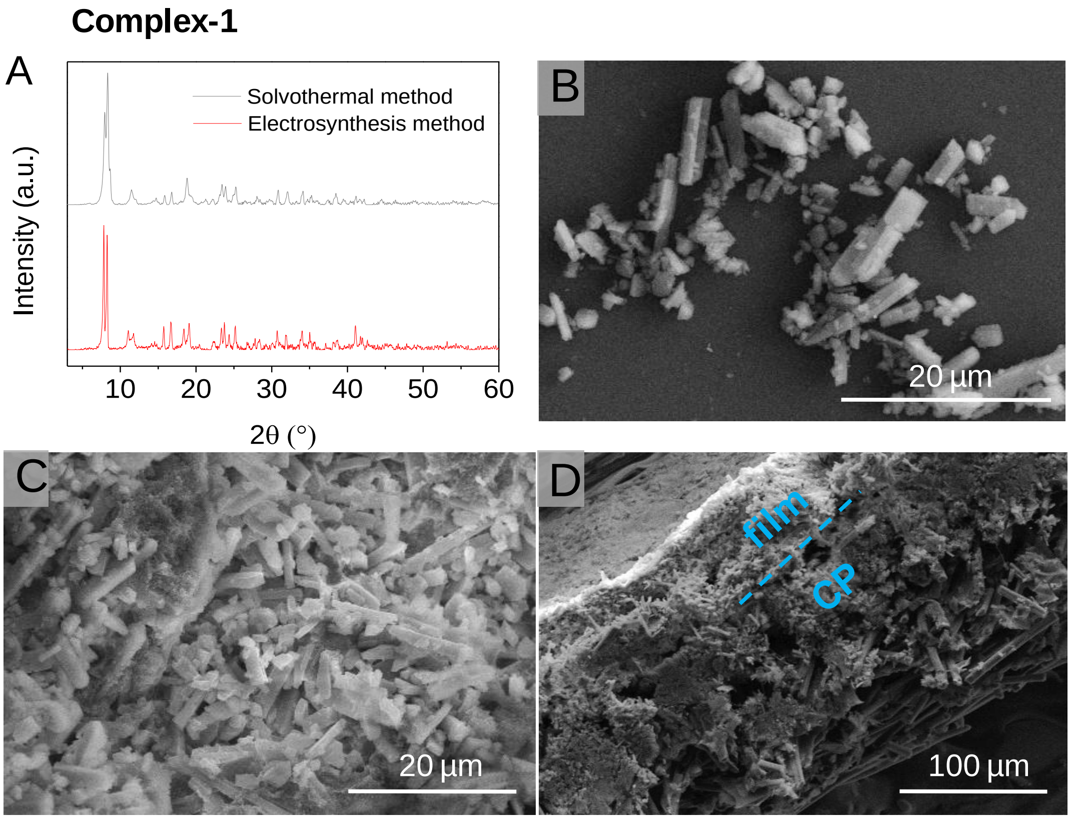

Supplementary Figure 36. (A) Comparison of XRD patterns of the Complex-1 prepared by electrodeposition and solvothermal methods. **(B)** SEM image of Complex-1 powder synthesized by solvothermal method. **(C)** SEM image of top-view of Complex-1 synthesized by solvothermal method on CP substrate. **(D)** SEM image of side-view of Complex-1 synthesized by solvothermal method on CP substrate.

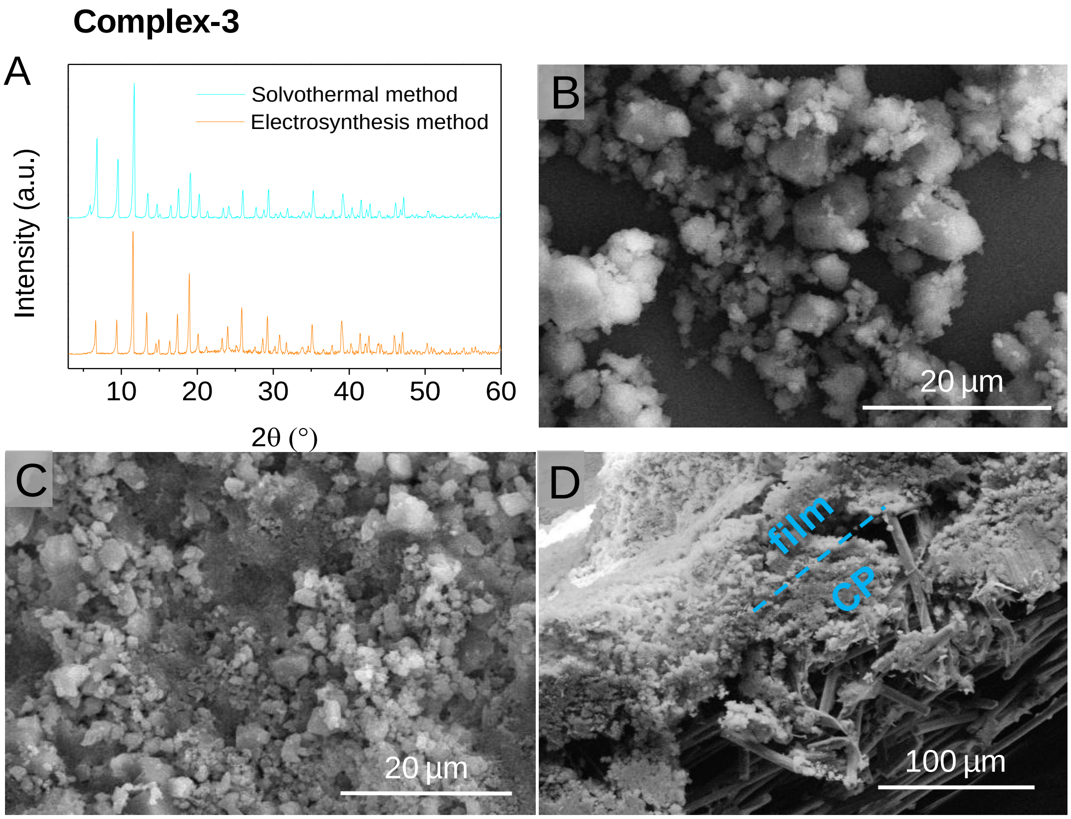

Supplementary Figure 37. (A) Comparison of XRD patterns of Complex-3 prepared by electrodeposition and solvothermal methods. **(B)** SEM image of Complex-3 powder synthesized by solvothermal method. **(C)** SEM image of top-view of Complex-3 synthesized by solvothermal method on CP substrate. **(D)** SEM image of side-view of Complex-3 synthesized by solvothermal method on CP substrate.

4.2 Characterization of the catalyst before and after electrolysis

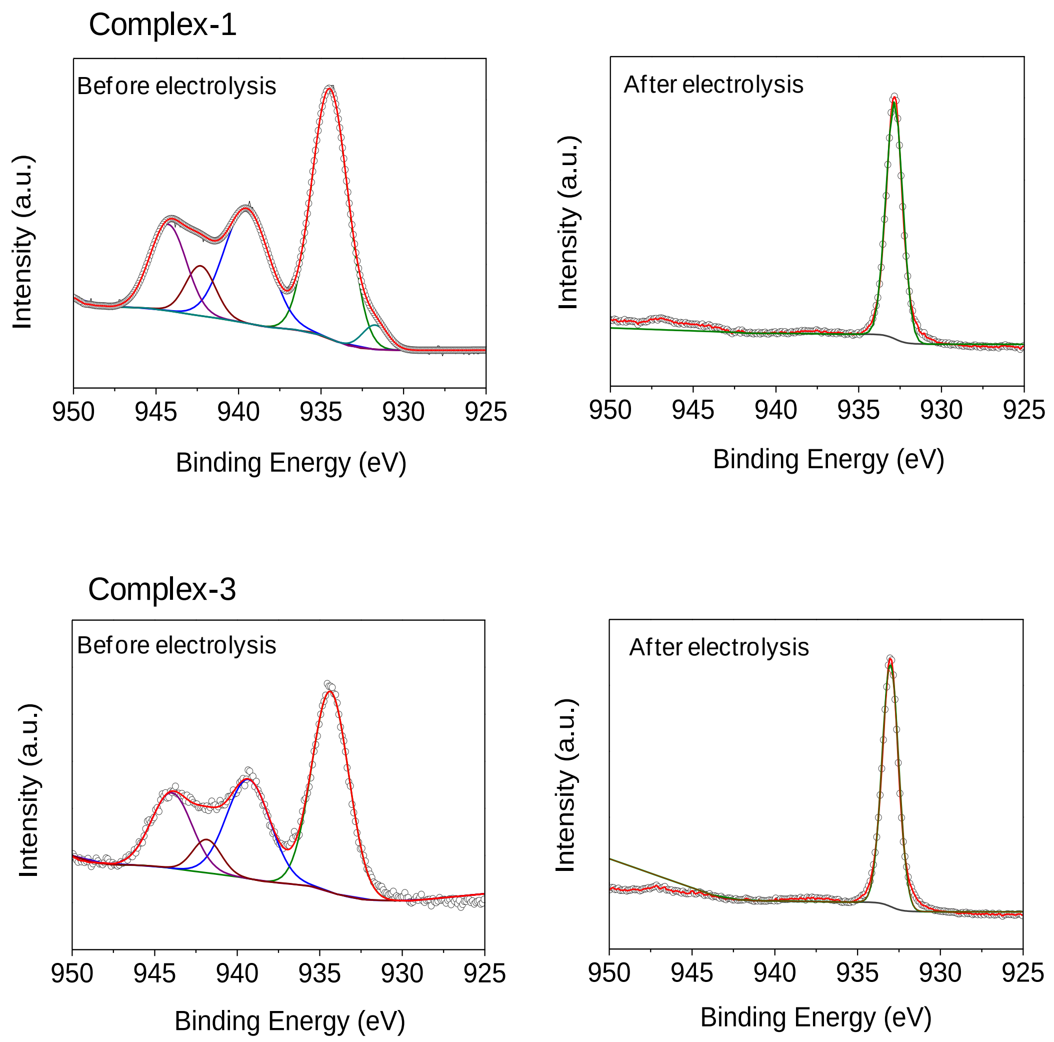

Supplementary Figure 38. Cu 2p$_{3/2}$ XPS spectra of Complex-1 and Complex-3 before and after electrolysis.

4.3 Reduction of CO_2 over Cu-Cu_2O via Cu-Complexes prepared by solvothermal method

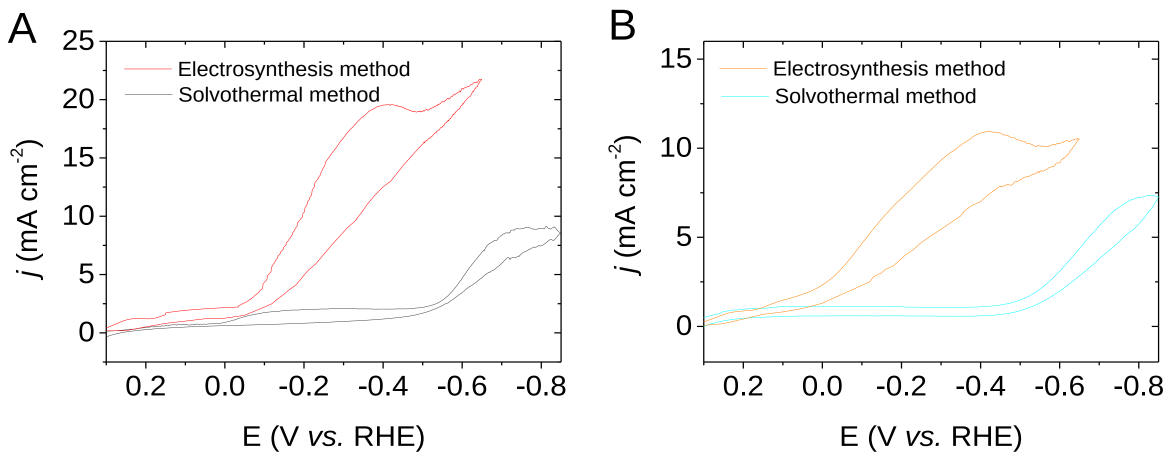

Supplementary Figure 39. Comparison of catalytic performances of Cu-Cu_2O-1 **(A)** and Cu-Cu_2O-3 **(B)** electrodes fabricated by electrodeposition and solvothermal methods.

Supplementary Table 12. The catalytic performances Cu-Cu_2O-1 and Cu-Cu_2O-3 electrodes synthesized via electrosynthesis and solvothermal methods for electrocatalytic reduction of CO_2 at optimized potentials.

Cu-Cu_2O-1 electrode:

Electrode	E_{app} (V vs RHE)	η (V)	j_{tot} (mA cm^{-2})	$FE_{Acetic\ acid}$ (%)	$FE_{Ethanol}$ (%)	FE_{CO} (%)	FE_{CH4} (%)	FE_{H2} (%)	FE_{C1}[a] (%)	FE_{C2}[b] (%)
Electrodeposition method	-0.4	0.484 (ethanol)<br>0.53 (acetic acid)	11.5±0.3	48.5±2.3	32.2±1.5	1.5±0.2	9.6±1.5	6.8±0.3	11.1±0.3	80.7±0.3
Solvothermal method	-0.7	0.784 (ethanol)<br>0.83 (acetic acid)	7.3±0.5	28.6±1.0	13.2±1.3	2.1±0.3	3.5±0.3	55.6±3.9	5.6±0.6	41.8±2.3

[a] summa of C_1 products;
[b] summa of C_2 products.

Cu-Cu_2O-3 electrode:

Electrode	E_{app} (V vs RHE)	η (V)	j_{tot} (mA cm^{-2})	$FE_{Acetic\ acid}$ (%)	$FE_{Ethanol}$ (%)	FE_{CO} (%)	FE_{CH4} (%)	FE_{H2} (%)	FE_{C1}[a] (%)	FE_{C2}[b] (%)
Electrodeposition method	-0.4	0.484 (ethanol)<br>0.53 (acetic acid)	7.3±0.2	17.5±0.3	39.2±2.0	13.8±0.8	14.5±2.3	11.8±1.3	28.3±3.1	56.7±3.3
Solvothermal method	-0.7	0.784 (ethanol)<br>0.83 (acetic acid)	4.9±0.3	8.6±0.5	32.0±3.2	12.2±0.5	4.0±2.3	50.3±3.5	16.2±2.8	40.6±3.7

[a] summa of C_1 products;
[b] summa of C_2 products.

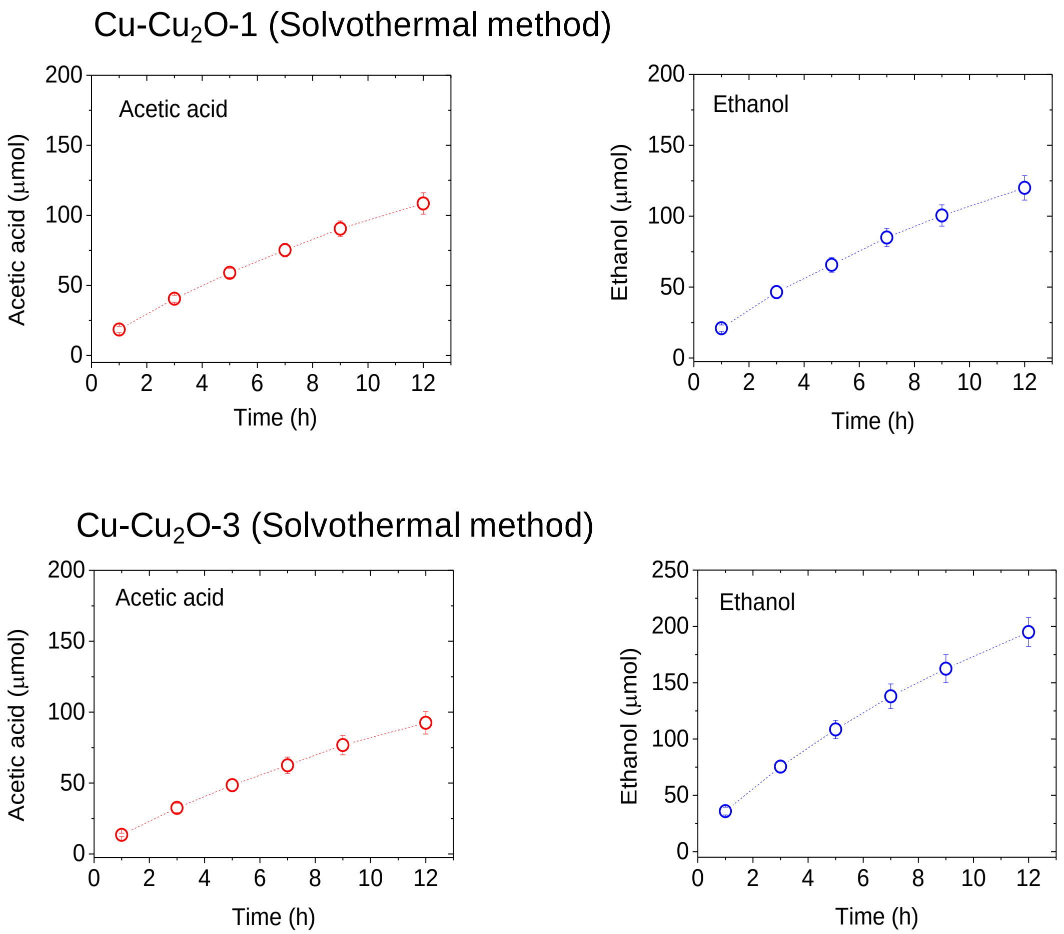

Supplementary Figure 40. The amounts of the liquid products generated from CO_2 reduction at different times over Cu-Cu_2O-1 and Cu-Cu_2O-3 that derived from Complex-1 and Complex-3 prepared by solvothermal method at -0.7 V vs RHE. The error bars represent the standard deviation of three measurements.

4.4 EIS study of the two methods

In this work, we performed the electrochemical impedance spectroscopy (EIS)[86,87]. To measure the solution resistance (R_s), charge transfer resistance (R_{ct}) between catalyst surface and the reactant, as well as film resistance (R_f) between the Cu-Cu_2O and substrate (Cu or carbon paper) for Cu-Cu_2O-1 and Cu-Cu_2O-3 electrodes prepared by two methods (Cu-Complex that prepared by in situ electrosynthesis and solvothermal methods after electrolysis for 5h). The impedance spectra was recorded in CO_2 saturated KCl aqueous solution at an open circuit potential (OCP) with an amplitude of 5 mV of 10^{-2} to 10^{-5} Hz (Supplementary Fig. 41). Two electrical equivalent circuits were used in simulation of impedance behavior of the electrode films from the experimentally obtained impedance data. The simulated results are given in Supplementary Table 13. It indicates that R_s was nearly independent of the method for preparation of the electrodes. However, the two components of the impedance R_{ct} and R_f with in situ synthesized Cu-Cu_2O were much lower than that prepared by solvothermal method. This indicates that surface of electrode prepared via electrosynthesized method has less effective barriers to charge transfer. It was properly due to better coverage and better adherent of the catalyst to the substrate electrode compared to the solvothermal method. The conductivities of substrates also provide a promising support to enhance the charge transport. Even though coatings of solvothermal method covered a large fraction of the surface of the panels, the poor charge transfer behavior appeared to be dominated by defects in the interfacial conductivity. The EIS result confirms that charge transfer can easily occur on the Cu-Cu_2O-1 or Cu-Cu_2O-3 electrodes prepared by electrosynthesis method.

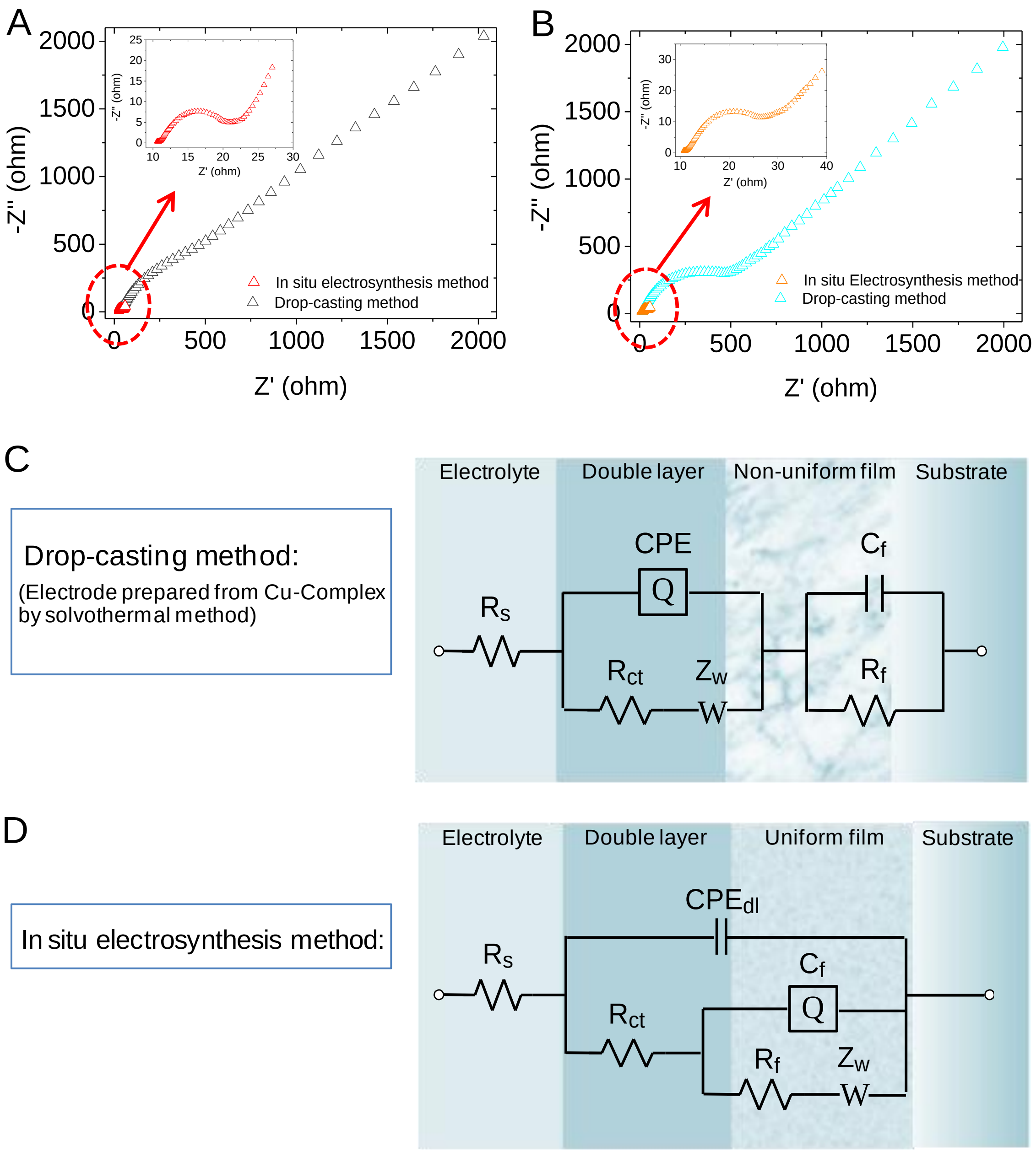

Supplementary Figure 41. (A) EIS study of Cu-Cu_2O-1 electrodes prepared by two methods in CO_2 saturated 0.1M KCl solution after electrolysis for 5h. **(B)** EIS study of Cu-Cu_2O-3 electrodes prepared by two methods in CO_2 saturated 0.1M KCl solution. **(C)** Randles' equivalent circuit used for fitting the experimental impedance data of the electrode prepared by solvothermal method: solution resistance (R_s), constant phase element (CPE) of the electrical double layer, electron transfer resistance (R_{ct}), Warburg-type impedance (Z_w), film capacitance (C_f) and film resistance (R_f). **(D)** Randles' equivalent circuit used for fitting the experimental impedance data of the electrode prepared by in situ electrodeposition method: solution resistance (R_s), double layer capacitance (CPE_{dl}), electron transfer resistance (R_{ct}), film capacitance (C_f), film resistance (R_f) and Warburg-type impedance (Z_w).

Supplementary Table 13. EIS characterization of various electrodes. Values of the main parameters of Randles equivalent circuit elements obtained by fitting the EIS spectra with Randles' equivalent circuit and R(Q(RW))(CR) and R(C(R(Q(RW)))) at OCP.

A:

Entry	Electrode	R_s ($\Omega \cdot cm^2$)	R_{ct} ($\Omega \cdot cm^2$)	R_f ($\Omega \cdot cm^2$)
1	Cu-Cu_2O-1 (Solvothermal method)	12.0	408.1	200.5
2	Cu-Cu_2O-3 (Solvothermal method)	12.3	512.3	248.6
3	Cu-Cu_2O-1 (Electrodeposition method)	10.7	11.8	9.6
4	Cu-Cu_2O-3 (Electrodeposition method)	11.6	19.2	12.5

B:

Entry	Sample	R_{ct} ($\Omega \cdot cm^2$)	
		Before electrolysis	After electrolysis
1	Complex-1	4.3	11.8
2	Complex-2	4.8	14.5
3	Complex-3	5.5	19.2
4	Complex-4	12.2	18.1
5	Complex-5	28.6	18.5
6	Complex-6	128.3	19.8

4.5 Reduction of CO_2 over Cu-Cu_2O derived from Cu-Complex with different electrodepositon time

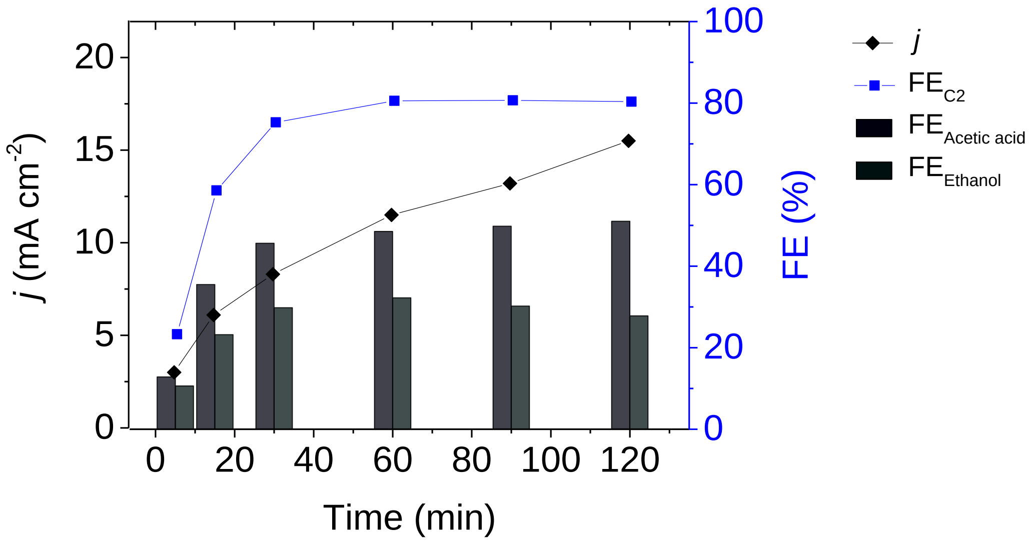

Supplementary Figure 42. Current densities and Faradaic efficiencies of C_2 product in 0.1 M KCl electrolyte using Cu-Cu_2O-1 electrode that derived from Complex-1 with electrodeposition time from 5 min to 2 h.

Supplementary note 5. Electrochemical reduction of CO_2 over other Cu-Cu_2O electrodes

5.1 Current densities and selectivities of C_2 products over various Cu-Cu_2O electrodes

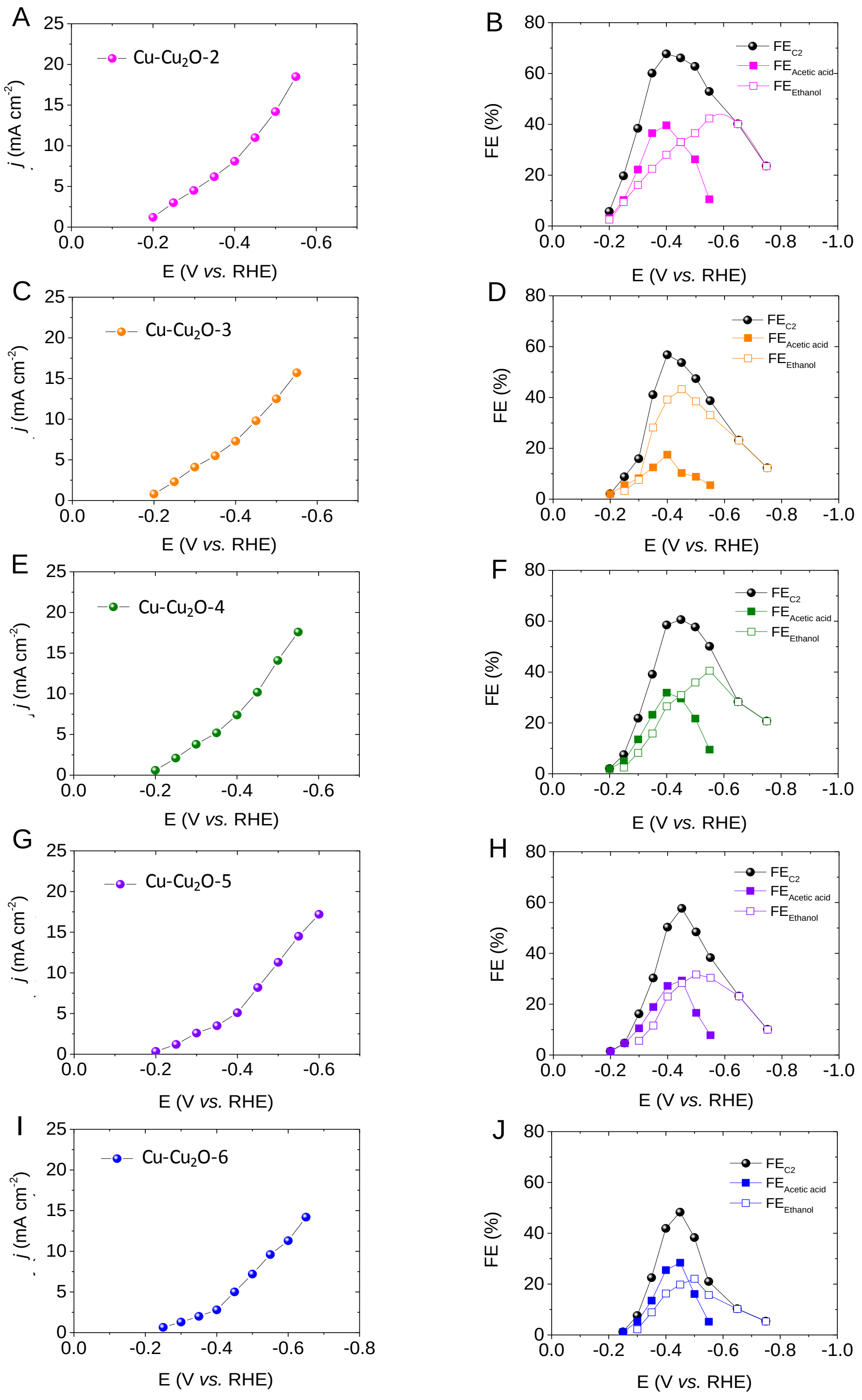

Supplementary Figure 43. Total current density and Faradaic efficiency over the Cu-Cu_2O electrodes at different applied potentials.

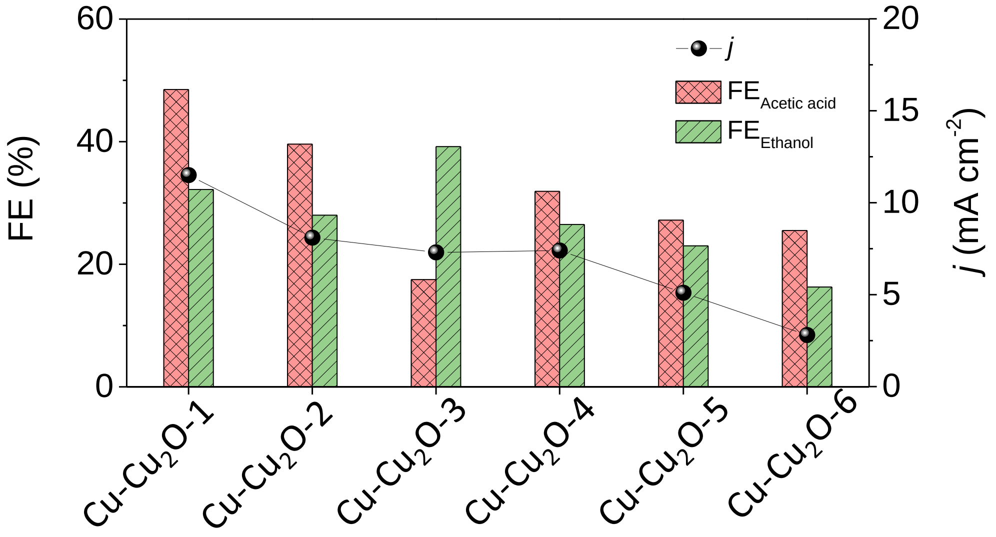

Supplementary Figure 44. Formation of C_2 products over various Cu-Cu_2O electrodes. The result indicates that the active surface Cu^I or Cu^0 sites alone do not improve the efficiencies of CO_2RR and indeed deteriorate the efficiency. It is synergy between surface Cu^I and surface Cu^0 that improves significantly the kinetics and thermodynamics of both CO_2 activation and CO dimerization, while making C_1 unfavorable, thereby boosting the efficiency and selectivity of CO_2RR to C_2 products.

5.2 Production rate of C_2 products over various Cu-Cu_2O electrodes

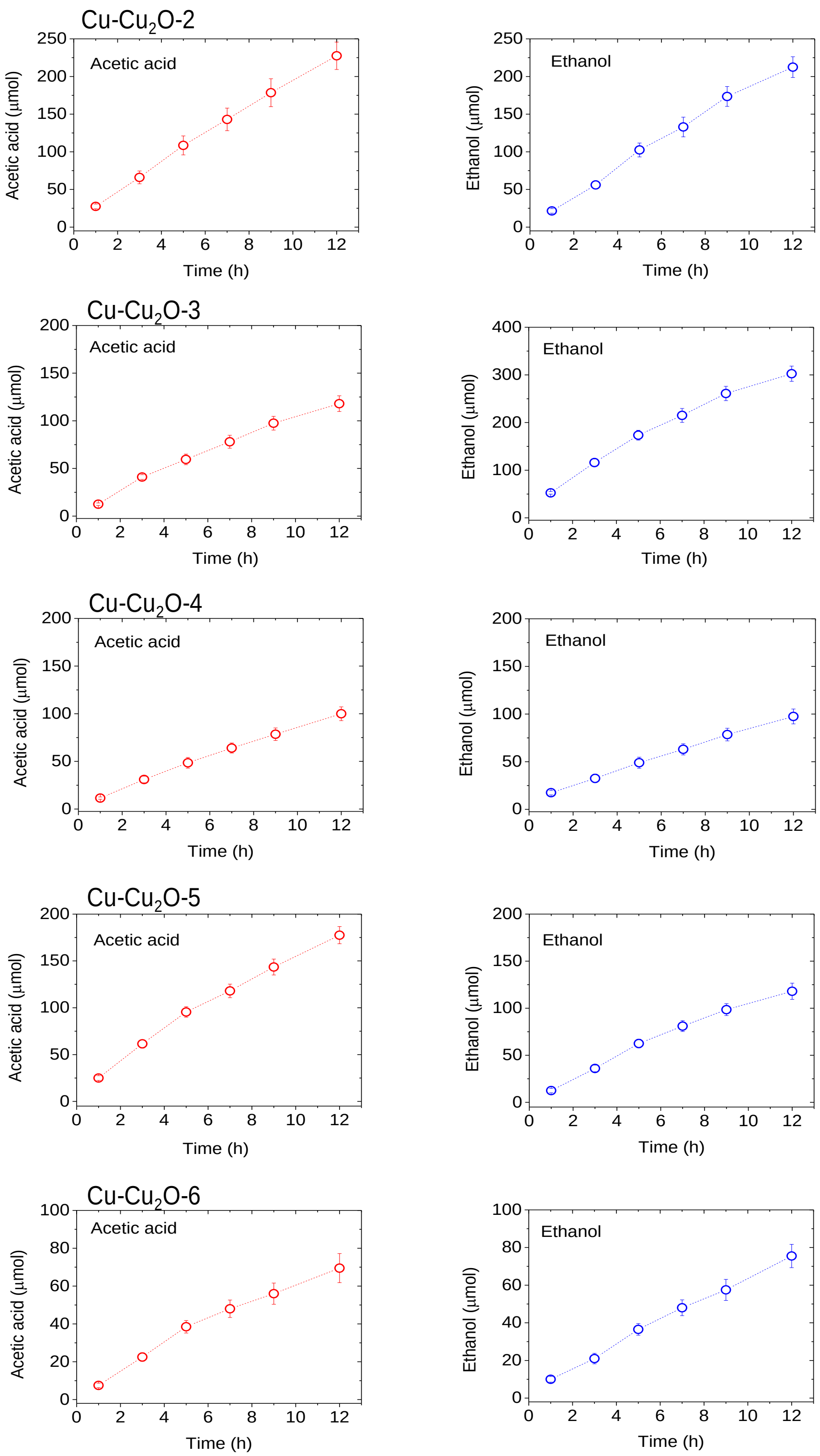

Supplementary Figure 45. The amounts of the liquid products generated from CO_2 reduction at different times over various Cu-Cu_2O electrodes at -0.4 V vs RHE. The error bars represent the standard deviation of three measurements.

Supplementary note 6. Proposed reaction mechanism for the C-C bond formation

1. *Test with possible intermediates.* With the aim to understand the mechanistic pathway towards the formation of acetic acid and ethanol, some tests of CO_2 reduction were made in the presence of the possible reaction intermediates, such as CO, formic acid, formaldehyde, acetaldehyde and acetic acid. Before testing the system with adding of possible intermediates, the air in cathode compartment was replaced by N_2 via bubbling the electrolyte using pure N_2. Then possible intermediate was added and electrolysis was started. The results are given in Supplementary Table 14. When CO or formaldehyde was used, acetic acid and ethanol were formed with high rates on Cu-Cu_2O-1/Cu electrode, respectively, indicating that CO and formaldehyde are a possible intermediate towards the formation of C_2 products. In the presence of acetaldehyde large amount of ethanol was formed, but acetic acid was not detected, indicating that acetaldehyde was a possible intermediate towards ethanol, but not for acetic acid. However, adding of formic acid only produced trace amount of ethanol and acetic acid, suggesting that formic acid was not the main intermediate of C_2 products. Moreover, adding of acetate did not yield detectable reduction product, this result suggests that acetate cannot be reduced further under our experimental condition, which is in agreement with the conclusion of the previous study[88]. All the results demonstrate that CO and formaldehyde are possible intermediates for both ethanol and acetic acid, and acetaldehyde is the possible intermediate for ethanol. The conclusion is the same when Cu-Cu_2O-3/Cu electrode was used, as shown in Supplementary Table 14.

2. *Proposed mechanism for the formation of C_2 products.* The experimental results discussed above support the tentative mechanistic pathway. Supplementary Scheme 2 shows the scheme of the possible mechanistic pathway for the electrocatalytic production of acetic acid and ethanol. The three key steps we focus on are (1) CO_2 activation, which we previously showed to be the rate determining step for $CO_2^{\cdot -}$ free radical; (ii) C_1 product formation, which was found to compete with C_2 products; (iii) CO dimerization, which can be important *COCHO or *COCO intermediate for C_2 products formation. After a first step of reduction with the initial formation of the radical anion $CO_2^{\cdot -}$, the radical anion $CO_2^{\cdot -}$ may strongly interact with the electrode surface depending on the nature of the electrocatalyst. This is a rate-determing step which can be detected from the Tafel slop of the reaction. The catalytic sites may stabilize $CO_2^{\cdot -}$, which can further reduce to more hydrogenated species. The experimental evidence suggests that after one proton and one electron transfer, CO_2 could be reduced to either *CHO or *COO adsorbed on the catalytic surface. Early mechanistic studies found that formic acid cannot be reduced to other products, suggesting that the mechanistic pathway toward formic acid is thus separate from the hydrocarbon pathway, which must go through CO. Therefore, the reaction is divided into two paths subsequently. In the formation route of acetic acid, *CO could be further reduced at low overpotential and undergoes a CO dimerization step mediated by electron transfer rendering a *COCHO intermediate. The intermediate *COCHO can further reduce at the electrode surface until a –CH_3COO^- species is formed. It is noteworthy that these half-reactions occur in a strong reducing environment. That is the electrons coming from the anode side through an external circuit and the protons reaching the cathode from the Nafion membrane in direct contact with the electrocatalyst.

At this point, the CH_3COO^- species may desorb to form acetic acid. In the formation route of ethanol, the*COCHO or *COCO both can be considered as the precursor for the formation of ethanol. The C-C bond formation is subsequently formed and undergoes a CH_3CHO intermediate and reduced to ethanol. Therefore, the pathway towards ethanol should be isolated by two routes until reaching the final CH_3CHO intermediate. This tentative mechanistic pathway is able to explain the experimental observations that we obtained in our electrocatalytic tests.

6.1 Possible reaction intermediates

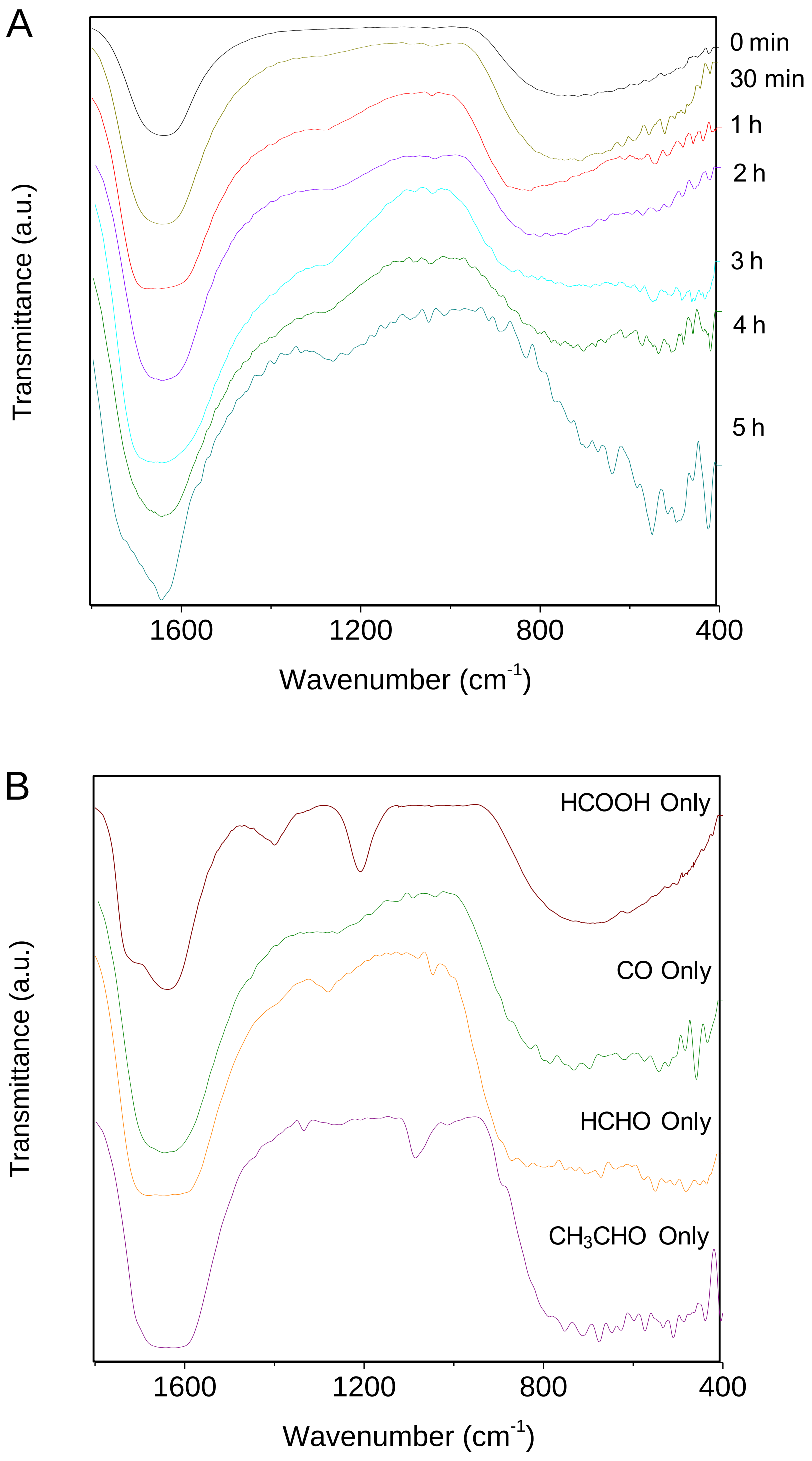

Supplementary Figure 46. IR pectra the electrolyte phase after electrolysis. (A) Electrolysis of CO_2 at different times. **(B)** Electrolysis in the presence of possible intermediates. The formation of COO^- can be verified from the peaks at 1352 cm^{-1} (bridge-bonded) and 1036 cm^{-1} (C-OH)[84]. The bands at 1085 cm^{-1} and 881 cm^{-1} are C–O stretch of ethanol. In addition, the peaks 637 cm^{-1} and 431 cm^{-1} in the fingerprint region can be assigned to the characteristic peak of ethanol[89].

Supplementary Table 14. Electrolysis over Cu-Cu_2O-1/Cu and Cu-Cu_2O-3/Cu electrodes in the presence of possible reaction intermediates at -0.4 V vs RHE in 0.1 M KCl electrolyte.

Reaction	Production rate (μmol/h)		
	Formic acid	Acetic acid	Ethanol
**Cu-Cu_2O-1**			
without CO_2	-	-	-
CO_2 only	-	25.8±3.3	21.7±2.5
HCOOH only	-	2.5±2.8	5.0±4.6
CO only	-	26.3±2.0	23.0±2.2
HCHO only	10.4±0.3	32.5±1.2	30.0±1.0
CH_3CHO only	-	-	198.5±3.6
CH_3COOH only	-	-	-
CO_2 + CO	-	30.4±0.1	28.6±0.2
CO_2 + HCHO	-	38.3±0.7	47.5±1.5
CO_2 + HCOOH	-	25.5±0.3	-
CO_2 + CH_3CHO	-	32.2±1.3	218.6±4.5
CO_2 + CH_3COOH	-	23.0±0.8	-
**Cu-Cu_2O-3**			
without CO_2	-	-	-
CO_2 only	-	14.1±0.4	17.4±0.6
HCOOH only	-	2.3±1.9	2.7±2.1
CO only	-	16.8±0.1	19.8±0.6
HCHO only	7.6±0.4	16.7±0.2	21.4±0.4
CH_3CHO only	-	0.6±0.4	144.5±2.8
CH_3COOH only	-	-	-
CO_2 + CO	-	17.2±0.8	35.0±2.3
CO_2 + HCHO	6.0±0.3	19.0±0.9	20.5±1.2
CO_2 + HCOOH	-	18.2±0.8	-
CO_2 + CH_3CHO	-	21.0±1.1	170.0±4.0
CO_2 + CH_3COOH	-	17.3±0.6	-

6.2 Possible reaction mechanism

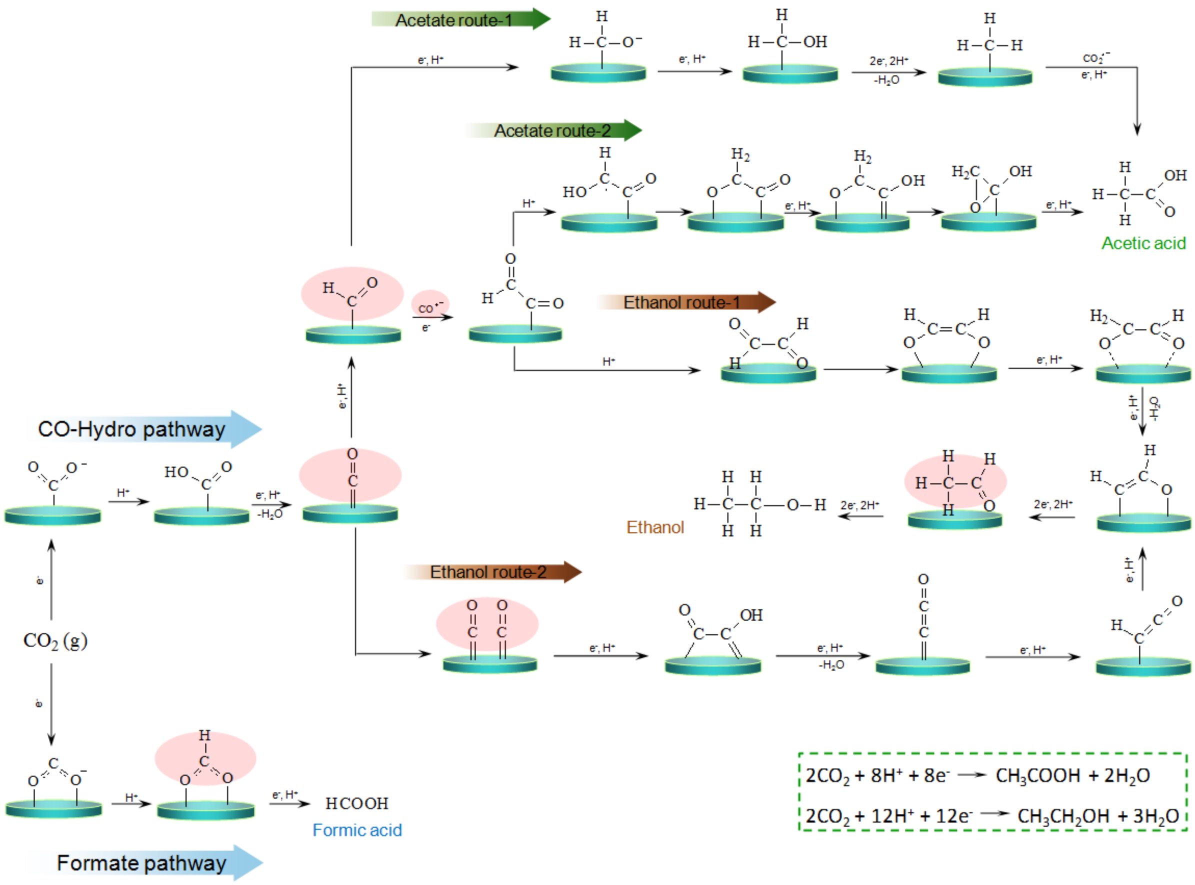

Supplementary Scheme 2. Proposed pathways for electrocatalytic reduction of CO_2 to C_2 products on the Cu-Cu_2O electrode surface.

Supplementary note 7. Crystallogrphic data

Supplementary Table 15. Crystallogrphic data for Complex-1, Complex-4 and Complex-5.

Compound	Complex-1	Complex-4	Complex-5
Formula	$C_{20}H_{44}Cu_4O_{36}$	$C_{18}H_{22}CuO_{18}$	$C_{14}H_{14}CuN_2O_{11}$
Molecular weight	1114.71	589.89	449.81
Crystal system	Monoclinic	Monoclinic	Monoclinic
Space group	I 1 2/c 1	P 1 21 1	P 1 c 1
a (Å)	9.540(4)	11.7001(3)	13.395(3)
b (Å)	18.119(5)	6.6745(2)	10.139(2)
c (Å)	12.077(2)	28.4761(6)	13.836(3)
α (°)	90	90	90
β (°)	112.67	95.390(2)	114.442
γ (°)	90 °	90	90
Volume ($Å^3$)	1926.3(11)	2213.93(10)	1710.8(6)
Z	2	4	4
D_{calc} ($mg\ m^{-3}$)	1.922	1.770	1.746
F (000)	1136	1212	916
μ (mm^{-1})[a]	2.299	1.081	1.344
Crystal size (mm)	0.298 × 0.171 × 0.135	0.185 × 0.041 × 0.027	0.174 × 0.125 × 0.067
θ_{min} to θ_{max} (°)[b]	2.14 to 27.464	1.748 to 31.431	1.670 to 27.489
Total reflections	12706	21612	13009
Total unique reflections (R_{int})	2204 (0.0591)	12670 (0.0449)	7232 (0.0313)
Number of refined parameters	139	678	509
Number of observed data	2204	12670	7232
Restraints	0	37	2
R_1[c], wR_2[d]	0.0269, 0.0728	0.0734, 0.1833	0.0397, 0.0959
S[e]	1.104	1.027	1.064
Largest diff. peak and hole (e $Å^{-3}$)	0.479 and -0.535	0.838 and -0.711	0.408 and -0.432

[a]Absorption coefficient; [b]Theta range for data collection; [c]$R_1 = \sum||F_o| - |F_c||/\sum|F_o|$ for data with $I>2\sigma$; [d]$wR_2 = [\sum[w(F_o^2 - F_c^2)^2]/\sum[w(F_o^2)^2]]^{1/2}$ (for all data); [e]Goodness of fit $S = [\sum w(F_o^2 - F_c^2)^2/(n - p)]^{1/2}$, where n is the number of reflections and p is the number of parameters.

Supplementary Table 16. Selected bond distances (Å) and angles (°) for complex-1[a].

Complex-1					
Cu1-O1	2.0107(13)	O9-H9A	0.8499	Cu1-O7-H7A	123.7
Cu1-O3#1	1.9568(13)	O9-H9B	0.85	Cu1-O7-H7B	121.8
Cu1-O5	1.9519(14)	O8-H8A	0.85	H7A-O7-H7B	106.6
Cu1-O6	2.2382(14)	O8-H8B	0.8499	O1-C1-C2	119.08(14)
Cu1-O7	1.9550(13)	O1-Cu1-O6	86.37(5)	O2-C1-O1	122.71(15)
O1-C1	1.272(2)	O3#1-Cu1-O1	175.08(5)	O2-C1-C2	118.18(15)
O2-C1	1.253(2)	O3#1-Cu1-O6	93.14(6)	C2#2-C2-C1	123.88(9)
O3-C5	1.273(2)	O5-Cu1-O1	93.89(6)	C3-C2-C1	116.66(14)
O4-C5	1.243(2)	O5-Cu1-O3#1	91.01(6)	C3-C2-C2#2	119.43(10)
O5-H5A	0.85	O5-Cu1-O6	90.01(6)	C2-C3-H3	119.5
O5-H5B	0.85	O5-Cu1-O7	173.50(5)	C4-C3-C2	121.01(15)
O6-H6A	0.85	O7-Cu1-O1	88.88(5)	C4-C3-H3	119.5
O6-H6B	0.8501	O7-Cu1-O3#1	86.30(6)	C3-C4-C4#2	119.55(10)
O7-H7A	0.85	O7-Cu1-O6	96.04(6)	C3-C4-C5	119.30(15)
O7-H7B	0.85	C1-O1-Cu1	106.43(11)	C4#2-C4-C5	121.09(9)
C1-C2	1.505(2)	C5-O3-Cu1#3	126.88(12)	O3-C5-C4	115.15(15)
C2-C2#2	1.402(3)	Cu1-O5-H5A	125.6	O4-C5-O3	126.32(16)
C2-C3	1.398(2)	Cu1-O5-H5B	118.4	O4-C5-C4	118.53(15)
C3-H3	0.95	H5A-O5-H5B	109.6	H9A-O9-H9B	86
C3-C4	1.394(2)	Cu1-O6-H6A	99	H8A-O8-H8B	104.5
C4-C4#2	1.401(3)	Cu1-O6-H6B	117.6		
C4-C5	1.509(2)	H6A-O6-H6B	103.5		

[a]Symmetry transformations used to generate equivalent atoms: For Complex-1: #1 -x+1/2,y-1/2,-z+1; #2-x+1,y,-z+3/2; #3 -x+1/2,y+1/2,-z+1; #4 x-1/2,-y+1/2,z; #5 x+1/2,-y+1/2,z; #6 x,-y,z+1/2; #7 -x+1,-y+1,-z+1; #8 x+1/2,y-1/2,z-1/2; #9 -x+1,y,-z+1/2.

Supplementary Table 17. Hydrogen bonds for Complex-1 [Å and °]

D-H...A	d(D-H)	d(H...A)	d(D...A)	<(DHA)
O5-H5A...O1#4	0.85	1.97	2.804(2)	165.5
O5-H5B...O6#2	0.85	1.84	2.689(2)	176.9
O6-H6A...O2#5	0.85	1.84	2.6702(19)	165
O6-H6B...O9#6	0.85	1.83	2.675(2)	171.9
O7-H7A...O8	0.85	1.86	2.702(2)	167.8
O7-H7B...O4#7	0.85	1.84	2.6846(19)	171
O9-H9A...O3#8	0.85	2.07	2.903(2)	166
O9-H9B...O8#9	0.85	2.19	2.996(2)	159.2

Supplementary Table 18. Selected bond distances (Å) and angles (°) for Complex-4[a]

Complex-4					
C1-C2	1.447(11)	O10A-H10A	0.84	Cu1-O14-H14B	103.4
C1-C6	1.388(11)	O12A-H12C	0.84	H14A-O14-H14B	116.6
C1-C7	1.533(11)	O13A-H13D	0.8849	Cu1-O15-H15A	131.1
C2-C3	1.398(11)	O13A-H13E	0.8851	Cu1-O15-H15B	115.4
C2-C8	1.465(11)	O14A-H14D	0.8552	H15A-O15-H15B	104.1
C3-H3	0.95	O14A-H14E	0.8594	H15B-O15-Cu1	115.4
C3-C4	1.405(11)	O15A-H15C	0.8743	H15B-O15-H15A	104.1
C4-H4	0.95	O15A-H15D	0.8763	H15B-O15-H15B	0
C4-C5	1.390(12)	O18-H18A	0.8498	C2A-C1A-C7A	119.7(7)
C5-H5	0.95	O18-H18B	0.8504	C6A-C1A-C2A	117.6(8)
C5-C6	1.419(11)	O20-H20A	0.8402	C6A-C1A-C7A	122.7(7)
C6-C9	1.475(11)	O20-H20B	0.8501	C1A-C2A-C3A	119.1(8)
C7-O1	1.247(12)	O20-H20B	0.8501	C1A-C2A-C8A	122.0(7)
C7-O2	1.202(12)	O19-H19A	0.8496	C3A-C2A-C8A	118.8(8)
C8-O3	1.189(10)	O19-H19B	0.8502	C2A-C3A-H3A	119
C8-O4	1.312(10)	O16-H16A	0.8501	C4A-C3A-C2A	122.0(8)
C9-O5	1.198(11)	O16-H16B	0.8502	C4A-C3A-H3A	119
C9-O6	1.345(10)	O17-H17A	0.8501	C3A-C4A-H4AA	120.7
C10-C11	1.396(11)	O17-H17B	0.85	C5A-C4A-C3A	118.7(8)
C10-C15	1.440(11)	O21-H21A	0.85	C5A-C4A-H4AA	120.7
C10-C17	1.526(11)	O21-H21B	0.8498	C4A-C5A-H5A	119.1
C11-C12	1.397(11)	C2-C1-C7	118.4(8)	C4A-C5A-C6A	121.8(8)
C11-C16	1.486(11)	C6-C1-C2	120.4(8)	C6A-C5A-H5A	119.1
C12-H12	0.95	C6-C1-C7	121.3(7)	C1A-C6A-C5A	120.6(8)
C12-C13	1.387(13)	C1-C2-C8	120.7(8)	C1A-C6A-C9A	119.5(8)
C13-H13	0.95	C3-C2-C1	118.3(8)	C5A-C6A-C9A	119.8(8)
C13-C14	1.426(12)	C3-C2-C8	121.0(7)	O1A-C7A-C1A	112.7(8)
C14-H14	0.95	C2-C3-H3	119.9	O2A-C7A-C1A	121.0(9)
C14-C15	1.381(11)	C2-C3-C4	120.1(7)	O2A-C7A-O1A	126.2(8)
C15-C18	1.476(11)	C4-C3-H3	119.9	O3A-C8A-C2A	122.0(8)
C16-O11	1.216(10)	C3-C4-H4	119.1	O3A-C8A-O4A	123.0(8)
C16-O12	1.328(10)	C5-C4-C3	121.8(8)	O4A-C8A-C2A	114.8(8)
C17-O7	1.272(13)	C5-C4-H4	119.1	O5A-C9A-C6A	122.5(8)
C17-O8	1.215(13)	C4-C5-H5	120.6	O5A-C9A-O6A	123.5(9)
C18-O9	1.214(10)	C4-C5-C6	118.8(8)	O6A-C9A-C6A	113.9(8)
C18-O10	1.306(10)	C6-C5-H5	120.6	C11A-C10A-C15A	118.2(8)
Cu1-O1	1.927(6)	C1-C6-C5	120.4(7)	C11A-C10A-C17A	122.1(7)
Cu1-O7	1.907(6)	C1-C6-C9	120.2(8)	C15A-C10A-C17A	119.6(7)
Cu1-O13	1.980(7)	C5-C6-C9	119.0(7)	C10A-C11A-C12A	119.7(8)
Cu1-O14	2.300(8)	O1-C7-C1	115.2(9)	C10A-C11A-C16A	119.9(7)

Cu1-O15	1.932(6)	O2-C7-C1	120.0(9)	C12A-C11A-C16A	120.3(7)
O4-H4A	0.84	O2-C7-O1	124.7(8)	C11A-C12A-H12B	118.7
O6-H6	0.84	O3-C8-C2	124.3(8)	C13A-C12A-C11A	122.6(8)
O10-H10	0.84	O3-C8-O4	123.0(8)	C13A-C12A-H12B	118.7
O12-H12A	0.84	O4-C8-C2	112.6(8)	C12A-C13A-H13C	121.1
O13-H13A	0.8843	O5-C9-C6	124.0(8)	C12A-C13A-C14A	117.7(8)
O13-H13B	0.8856	O5-C9-O6	124.9(8)	C14A-C13A-H13C	121.1
O14-H14A	0.85	O6-C9-C6	111.1(8)	C13A-C14A-H14C	118.7
O14-H14B	0.8501	C11-C10-C15	119.2(8)	C13A-C14A-C15A	122.5(9)
O15-H15A	0.8853	C11-C10-C17	122.3(7)	C15A-C14A-H14C	118.7
O15-H15B	0.8849	C15-C10-C17	118.5(8)	C10A-C15A-C18A	121.2(8)
O15-H15B	0.8849	C10-C11-C12	121.0(7)	C14A-C15A-C10A	119.3(8)
C1A-C2A	1.403(11)	C10-C11-C16	120.0(7)	C14A-C15A-C18A	119.5(8)
C1A-C6A	1.382(11)	C12-C11-C16	118.4(7)	O11A-C16A-C11A	122.9(8)
C1A-C7A	1.508(11)	C11-C12-H12	120.3	O11A-C16A-O12A	122.4(8)
C2A-C3A	1.404(11)	C13-C12-C11	119.5(8)	O12A-C16A-C11A	114.6(8)
C2A-C8A	1.507(11)	C13-C12-H12	120.3	O7A-C17A-C10A	111.8(9)
C3A-H3A	0.95	C12-C13-H13	119.5	O8A-C17A-C10A	122.1(10)
C3A-C4A	1.359(13)	C12-C13-C14	120.9(8)	O8A-C17A-O7A	126.1(8)
C4A-H4AA	0.95	C14-C13-H13	119.5	O9A-C18A-C15A	122.5(8)
C4A-C5A	1.340(13)	C13-C14-H14	120.2	O9A-C18A-O10A	124.3(8)
C5A-H5A	0.95	C15-C14-C13	119.6(8)	O10A-C18A-C15A	113.1(8)
C5A-C6A	1.396(11)	C15-C14-H14	120.2	O1A-Cu1A-O13A	92.7(2)
C6A-C9A	1.519(12)	C10-C15-C18	120.4(8)	O1A-Cu1A-O14A	90.6(3)
C7A-O1A	1.310(12)	C14-C15-C10	119.7(8)	O1A-Cu1A-O15A	87.3(2)
C7A-O2A	1.237(12)	C14-C15-C18	119.9(7)	O7A-Cu1A-O1A	177.4(2)
C8A-O3A	1.224(11)	O11-C16-C11	123.6(8)	O7A-Cu1A-O13A	89.9(2)
C8A-O4A	1.296(10)	O11-C16-O12	124.4(8)	O7A-Cu1A-O14A	89.0(3)
C9A-O5A	1.221(11)	O12-C16-C11	111.9(7)	O7A-Cu1A-O15A	90.3(2)
C9A-O6A	1.328(10)	O7-C17-C10	116.1(9)	O13A-Cu1A-O14A	102.0(3)
C10A-C11A	1.382(11)	O8-C17-C10	119.2(9)	O15A-Cu1A-O13A	158.3(4)
C10A-C15A	1.401(11)	O8-C17-O7	124.6(8)	O15A-Cu1A-O14A	99.7(4)
C10A-C17A	1.517(11)	O9-C18-C15	123.5(8)	C7A-O1A-Cu1A	115.5(6)
C11A-C12A	1.402(11)	O9-C18-O10	123.4(8)	C8A-O4A-H4AB	109.5
C11A-C16A	1.504(12)	O10-C18-C15	113.1(7)	C9A-O6A-H6A	109.5
C12A-H12B	0.95	O1-Cu1-O13	88.8(3)	C17A-O7A-Cu1A	116.3(6)
C12A-C13A	1.349(13)	O1-Cu1-O14	87.5(3)	C18A-O10A-H10A	109.5
C13A-H13C	0.95	O1-Cu1-O15	92.8(2)	C16A-O12A-H12C	109.5
C13A-C14A	1.360(12)	O7-Cu1-O1	175.5(4)	Cu1A-O13A-H13D	143.5
C14A-H14C	0.95	O7-Cu1-O13	92.5(3)	Cu1A-O13A-H13E	126.1
C14A-C15A	1.395(11)	O7-Cu1-O14	96.8(3)	H13D-O13A-H13E	82.6
C15A-C18A	1.524(11)	O7-Cu1-O15	85.6(2)	Cu1A-O14A-H14D	116
C16A-O11A	1.234(11)	O13-Cu1-O14	86.2(3)	Cu1A-O14A-H14E	132.7
C16A-O12A	1.331(10)	O15-Cu1-O13	174.7(4)	H14D-O14A-H14E	101.5
C17A-O7A	1.328(13)	O15-Cu1-O14	98.9(3)	Cu1A-O15A-H15C	110.3

C17A-O8A	1.249(13)	C7-O1-Cu1	120.2(7)	Cu1A-O15A-H15D	112.8
C18A-O9A	1.216(11)	C8-O4-H4A	109.5	H15C-O15A-H15D	102.9
C18A-O10A	1.304(10)	C9-O6-H6	109.5	H18A-O18-H18B	101
Cu1A-O1A	1.972(6)	C17-O7-Cu1	120.5(6)	H20A-O20-H20B	117.2
Cu1A-O7A	1.970(6)	C18-O10-H10	109.5	H20B-O20-H20A	117.2
Cu1A-O13A	2.005(5)	C16-O12-H12A	109.5	H20B-O20-H20B	0
Cu1A-O14A	2.141(7)	Cu1-O13-H13A	112.8	H19A-O19-H19B	98.6
Cu1A-O15A	2.003(6)	Cu1-O13-H13B	125.4	H16A-O16-H16B	113.4
O4A-H4AB	0.84	H13A-O13-H13B	109.9	H17A-O17-H17B	118.5
O6A-H6A	0.84	Cu1-O14-H14A	132.3	H21A-O21-H21B	85.6

[a]Symmetry transformations used to generate equivalent atoms: For Complex-4: #1 -x+1,y-1/2,-z+1; #2-x+2,y-1/2,-z+1; #3 x,y+1,z; #4 x+1,y,z; #5 x-1,y,z; #6 -x+2,y+1/2,-z+1; #7 -x+1,y+1/2,-z+1; #8 x,y-1,z;

Table S19. Hydrogen bonds for Complex-4 [Å and °]

D-H...A	d(D-H)	d(H...A)	d(D...A)	<(DHA)
O4-H4A...O17#1	0.84	1.84	2.679(8)	173.7
O6-H6...O17#2	0.84	1.85	2.675(8)	165.2
O10-H10...O19#2	0.84	1.82	2.657(8)	173.2
O12-H12A...O19#1	0.84	1.96	2.647(8)	138.7
O13-H13A...O15A	0.88	1.94	2.784(10)	158.2
O13-H13B...O3	0.89	1.88	2.690(10)	151.1
O14-H14A...O8#3	0.85	2.31	3.123(11)	161.2
O14-H14B...O9A	0.85	2.24	2.967(10)	143.1
O15-H15A...O13A#4	0.89	1.95	2.789(8)	156.6
O15-H15B...O3A#4	0.88	1.93	2.788(9)	162.8
O4A-H4AB...O21#5	0.84	1.92	2.662(8)	145.8
O6A-H6A...O21	0.84	1.85	2.684(8)	175.9
O10A-H10A...O16#6	0.84	1.84	2.662(8)	166
O12A-H12C...O16#7	0.84	1.87	2.691(8)	165.6
O13A-H13D...O20#3	0.88	1.92	2.675(9)	142.4
O13A-H13E...O11A	0.89	1.91	2.691(8)	146.3
O14A-H14D...O2A#8	0.86	2.59	3.072(10)	117
O14A-H14D...O20	0.86	2.14	2.910(11)	149.1
O14A-H14E...O8A#8	0.86	2.57	3.125(10)	123.1
O14A-H14E...O18#1	0.86	2.13	2.914(11)	151
O15A-H15C...O11	0.87	2.31	2.977(10)	133.3
O15A-H15C...O5A	0.87	2.24	2.896(10)	131.7
O15A-H15D...O18#7	0.88	2.29	3.063(11)	147.8
O18-H18A...O8A#1	0.85	1.93	2.778(9)	172.6
O18-H18B...O5A#1	0.85	2.14	2.917(9)	151.6
O20-H20A...O2#5	0.84	2.08	2.917(8)	171.5
O20-H20B...O9#5	0.85	2.23	3.009(10)	152.1
O19-H19A...O2A#1	0.85	1.83	2.668(11)	171.1
O19-H19B...O1A#7	0.85	1.86	2.669(10)	159.1
O16-H16A...O1#2	0.85	1.94	2.788(11)	178.4
O16-H16B...O2#6	0.85	1.91	2.710(11)	157.5
O17-H17A...O8A#1	0.85	1.93	2.679(11)	147.1
O17-H17B...O7A#7	0.85	1.86	2.668(10)	157.1
O21-H21A...O7	0.85	1.92	2.761(10)	171
O21-H21B...O8#3	0.85	2.12	2.721(11)	126.9

Supplementary Table 20. Selected bond distances (Å) and angles (°) for complex-5[a]

Complex-5

C1-C2	1.389(8)	O19-H19A	0.85	C16-C15-C20	124.5(5)
C1-C6	1.497(8)	O19-H19B	0.8498	N3-C15-C16	121.5(5)
C1-N1	1.349(7)	O21-H21A	0.85	N3-C15-C20	114.0(4)
C2-H2	0.95	O21-H21B	0.8499	C15-C16-H16	120.4
C2-C3	1.370(9)	O20-H20A	0.8496	C17-C16-C15	119.3(5)
C3-H3	0.95	O20-H20B	0.8497	C17-C16-H16	120.4
C3-C4	1.380(8)	O17-H17A	0.8499	C16-C17-H17	120.5
C4-H4	0.95	O17-H17B	0.8499	C16-C17-C18	119.0(5)
C4-C5	1.390(7)	O18-H18A	0.8501	C18-C17-H17	120.5
C5-C7	1.496(7)	O18-H18B	0.8501	C17-C18-H18	120.7
C5-N1	1.337(7)	O22-H22A	0.8495	C19-C18-C17	118.5(5)
C6-O1	1.223(7)	O22-H22B	0.8498	C19-C18-H18	120.7
C6-O2	1.294(7)	C2-C1-C6	122.4(5)	C18-C19-C21	124.3(5)
C7-O3	1.290(7)	N1-C1-C2	121.8(5)	N3-C19-C18	121.9(5)
C7-O4	1.235(6)	N1-C1-C6	115.8(5)	N3-C19-C21	113.7(4)
C8-C9	1.396(7)	C1-C2-H2	120.5	O9-C20-C15	120.4(5)
C8-C13	1.505(7)	C3-C2-C1	118.9(5)	O9-C20-O10	125.2(5)
C8-N2	1.335(7)	C3-C2-H2	120.5	O10-C20-C15	114.4(4)
C9-H9	0.95	C2-C3-H3	120.1	O11-C21-C19	113.9(4)
C9-C10	1.377(8)	C2-C3-C4	119.9(5)	O12-C21-C19	121.2(5)
C10-H10	0.95	C4-C3-H3	120.1	O12-C21-O11	125.0(5)
C10-C11	1.383(8)	C3-C4-H4	120.9	C23-C22-C27	127.2(5)
C11-H11	0.95	C3-C4-C5	118.2(5)	N4-C22-C23	119.7(4)
C11-C12	1.389(7)	C5-C4-H4	120.9	N4-C22-C27	113.1(5)
C12-C14	1.499(8)	C4-C5-C7	122.7(5)	C22-C23-H23	120.7
C12-N2	1.326(6)	N1-C5-C4	122.5(5)	C24-C23-C22	118.6(5)
C13-O5	1.275(7)	N1-C5-C7	114.8(4)	C24-C23-H23	120.7
C13-O6	1.246(7)	O1-C6-C1	119.8(5)	C23-C24-H24	119.8
C14-O7	1.312(7)	O1-C6-O2	127.5(6)	C25-C24-C23	120.4(5)
C14-O8	1.221(6)	O2-C6-C1	112.7(5)	C25-C24-H24	119.8
Cu1-N1	1.996(4)	O3-C7-C5	113.4(4)	C24-C25-H25	120.8
Cu1-N2	1.910(4)	O4-C7-C5	120.3(5)	C24-C25-C26	118.5(5)
Cu1-O4	2.324(4)	O4-C7-O3	126.3(5)	C26-C25-H25	120.8
Cu1-O5	2.029(5)	C9-C8-C13	128.2(5)	C25-C26-C28	128.4(4)
Cu1-O7	2.037(4)	N2-C8-C9	119.8(5)	N4-C26-C25	119.5(5)
O2-H2A	0.82	N2-C8-C13	112.1(4)	N4-C26-C28	112.1(4)
O3-H3A	0.82	C8-C9-H9	121.1	O15-C27-C22	119.7(5)
C15-C16	1.392(7)	C10-C9-C8	117.9(5)	O15-C27-O16	125.6(5)
C15-C20	1.494(7)	C10-C9-H9	121.1	O16-C27-C22	114.7(4)
C15-N3	1.339(6)	C9-C10-H10	119.3	O13-C28-C26	119.0(4)

C16-H16	0.95	C9-C10-C11	121.4(5)	O13-C28-O14	125.8(5)
C16-C17	1.385(8)	C11-C10-H10	119.3	O14-C28-C26	115.2(4)
C17-H17	0.95	C10-C11-H11	121	N3-Cu2-O9	76.14(16)
C17-C18	1.399(8)	C10-C11-C12	117.9(5)	N3-Cu2-O12	75.95(15)
C18-H18	0.95	C12-C11-H11	121	N3-Cu2-O14	100.06(16)
C18-C19	1.388(7)	C11-C12-C14	127.6(5)	N3-Cu2-O16	99.23(15)
C19-C21	1.504(7)	N2-C12-C11	120.2(5)	N4-Cu2-N3	179.83(19)
C19-N3	1.346(6)	N2-C12-C14	112.2(4)	N4-Cu2-O9	103.92(15)
C20-O9	1.230(6)	O5-C13-C8	114.5(5)	N4-Cu2-O12	103.98(16)
C20-O10	1.296(6)	O6-C13-C8	119.2(5)	N4-Cu2-O14	80.10(16)
C21-O11	1.316(6)	O6-C13-O5	126.3(5)	N4-Cu2-O16	80.62(16)
C21-O12	1.211(6)	O7-C14-C12	114.6(4)	O9-Cu2-O12	152.05(13)
C22-C23	1.389(8)	O8-C14-C12	120.0(5)	O14-Cu2-O9	94.39(14)
C22-C27	1.501(7)	O8-C14-O7	125.4(6)	O14-Cu2-O12	91.98(14)
C22-N4	1.326(7)	N1-Cu1-O4	76.40(16)	O16-Cu2-O9	89.15(14)
C23-H23	0.95	N1-Cu1-O5	99.99(16)	O16-Cu2-O12	93.74(14)
C23-C24	1.388(8)	N1-Cu1-O7	99.50(17)	O16-Cu2-O14	160.68(15)
C24-H24	0.95	N2-Cu1-N1	176.49(18)	C15-N3-C19	119.8(4)
C24-C25	1.382(8)	N2-Cu1-O4	107.11(16)	C15-N3-Cu2	120.1(3)
C25-H25	0.95	N2-Cu1-O5	80.14(17)	C19-N3-Cu2	120.2(3)
C25-C26	1.393(7)	N2-Cu1-O7	80.41(17)	C22-N4-C26	123.3(4)
C26-C28	1.509(7)	O5-Cu1-O4	92.85(16)	C22-N4-Cu2	118.3(3)
C26-N4	1.337(6)	O5-Cu1-O7	160.51(16)	C26-N4-Cu2	118.5(3)
C27-O15	1.221(6)	O7-Cu1-O4	91.69(16)	C20-O9-Cu2	109.3(3)
C27-O16	1.300(6)	C1-N1-Cu1	122.0(4)	C20-O10-H10A	102.8
C28-O13	1.240(6)	C5-N1-C1	118.6(5)	C21-O11-H11A	109.5
C28-O14	1.269(6)	C5-N1-Cu1	119.3(4)	C21-O12-Cu2	109.0(3)
Cu2-N3	1.985(4)	C8-N2-Cu1	118.1(3)	C28-O14-Cu2	114.2(3)
Cu2-N4	1.905(4)	C12-N2-C8	122.9(4)	C27-O16-Cu2	113.4(3)
Cu2-O9	2.320(4)	C12-N2-Cu1	118.8(4)	H19A-O19-H19B	103.7
Cu2-O12	2.345(4)	C6-O2-H2A	99.9	H21A-O21-H21B	91.2
Cu2-O14	2.059(4)	C7-O3-H3A	111.5	H20A-O20-H20B	104.5
Cu2-O16	2.053(3)	C7-O4-Cu1	109.0(3)	H17A-O17-H17B	97.9
O10-H10A	0.82	C13-O5-Cu1	115.1(4)	H18A-O18-H18B	113.5
O11-H11A	0.84	C14-O7-Cu1	113.7(3)	H22A-O22-H22B	125.6

[a]Symmetry transformations used to generate equivalent atoms: For Complex-5: #1 x,-y,z+1/2; #2 x,-y+1,z-1/2; #3 x,y+1,z; #4 x+1,y,z; #5 x+1,-y+1,z+1/2; #6 x,-y+1,z+1/2.

Supplementary Table 21. Hydrogen bonds for Complex-5 [Å and °]

D-H...A	d(D-H)	d(H...A)	d(D...A)	<(DHA)
O2-H2A...O17#1	0.82	1.71	2.461(7)	150.7
O3-H3A...O19	0.82	1.64	2.446(6)	165.1
O10-H10A...O21#2	0.82	1.76	2.508(5)	151
O11-H11A...O22	0.84	1.76	2.598(5)	176.7
O19-H19A...O6#2	0.85	1.83	2.671(6)	170.1
O19-H19B...O20	0.85	1.79	2.533(7)	145.4
O21-H21A...O13#3	0.85	1.97	2.722(5)	146.3
O21-H21B...O6#2	0.85	1.88	2.652(6)	149.8
O17-H17A...O18	0.85	2.03	2.669(8)	131.8
O17-H17B...O22#4	0.85	2.22	3.017(6)	157.2
O18-H18A...O15#5	0.85	1.91	2.734(6)	162
O18-H18B...O7	0.85	1.98	2.826(7)	174.6
O22-H22A...O13#1	0.85	2.02	2.822(5)	157.8
O22-H22B...O16#6	0.85	1.88	2.728(5)	172.1

Supplementary references

1. Jeong, N. C., Samanta, B., Lee, C. Y., Farha, O. K. & Hupp, J. T. Coordination-chemistry control of proton conductivity in the iconic metal-organic framework material HKUST-1. *J. Am. Chem. Soc.* **134**, 51-54 (2011).
2. Köferstein, R. & Robl, C. Synthesis and crystal structure of two Cu^{II}-benzene-1,2,4,5-tetracarboxylates with three-dimensional open frameworks. *Z. Anorg. Allg. Chem.* **640**, 310-316 (2014).
3. Qin, C., Wang, X. L., Wang, E., Hu, C. & Xu, L. $[Cu_2(HBTC)_2(H_2O)_2(\mu_2\text{-}H_2O)] \cdot 2H_2O$: a new arm-shaped two-dimensional copper coordination polymer having both rhombic cavities and helical-like channels. *Inorg. Chem. Commun.* **7**, 788-791 (2004).
4. Ma, L. F., Li, C. P., Wang, L. Y. & Du, M. Co^{II} and Zn^{II} coordination frameworks with benzene-1,2,3-tricarboxylate tecton and flexible dipyridyl co-ligand: a new type of entangled architecture and a unique 4-connected topological network. *Cryst. Growth. Des.* **11**, 3309-3312 (2011).
5. van Albada, G. A., Ghazzali, M., Al-Farhan, K., Bouwman, E. & Reedijk, J. Three new pyridine-2, 6-dicarboxylate copper (II) compounds with coordinated pyrimidine-based ligands: Synthesis, characterization and crystal structures. *Polyhedron* **52**, 1059-1064 (2013).
6. Carson, C. G. et al. Synthesis and structure characterization of copper terephthalate metal-organic frameworks. *Eur. J. Inorg. Chem.* **2009**, 2338-2343 (2009).
7. Chui, S. S. Y., Lo, S. M. F., Charmant, J. P. H., Orpen, A. G. & Williams, L. D. A chemically functionalizable nanoporous material $[Cu_3(TMA)_2(H2O)_3]_n$. *Science* **283**, 1148-1150 (1999).
8. Liu, P. & Hensen, E. J. M. Highly efficient and robust $Au/MgCuCr_2O_4$ catalyst for gas-phase oxidation of ethanol to acetaldehyde. *J. Am. Chem. Soc.* **135**, 14032-14035 (2013).
9. Puigdollers, A. R., Schlexer, P., Tosoni, S. & Pacchioni, G. Increasing oxide reducibility: the role of metal/oxide interface in the formation of oxygen vacancies. *ACS Catal.* **7**, 6493-6531 (2017).
10. Cavalca, F. et al. Nature and distribution of stable subsurface oxygen in copper electrodes during electrochemical CO_2 reduction. *J. Phys. Chem. C* **121**, 25003-25009 (2017).
11. Kumar, B. et al. Reduced SnO_2 porous nanowires with a high density of grain boundaries as catalysts for efficient electrochemical CO_2-into-HCOOH conversion. *Angew. Chem. Int. Ed.* **56**, 3645-3649 (2017).
12. Lee, S., Kim, D. & Lee, J. Electrocatalytic production of C_3-C_4 compounds by conversion of CO_2 on a chloride-induced Bi-Phasic Cu_2O-Cu catalyst. *Angew, Chem. Int. Ed.* **54**, 14701-14705 (2015).
13. Kuhl, K. P., Hatsukade, T., Cave, E. R., Abram, D. N., Kibsgaard, J. & Jaramillo, T.

F. Electrocatalytic conversion of carbon dioxide to methane and methanol on transition metal surfaces. *J. Am. Chem. Soc.* **136**, 14107-14113 (2014).

14. Raciti, D., Livi, K. J. & Wang, C. Highly dense Cu nanowires for low-overpotential CO_2 reduction. *Nano Lett.* **15**, 6829-6835 (2015).

15. Min, S. X. et al. Low overpotential and high current CO_2 reduction with surface reconstructed Cu foam electrodes. *Nano Energy* **27**, 121-129 (2016).

16. Sen, S., Liu, D. & Palmore, G. T. R. Electrochemical reduction of CO_2 at copper nanofoams. *ACS Catal.* **4**, 3091-3095 (2014).

17. Kas, R. et al. Three-dimensional porous hollow fibre copper electrodes for efficient and high-rate electrochemical carbon dioxide reduction. *Nat. Commun.* **7**, 10748 (2016).

18. Merino-Garcia, I., Albo, J. & Irabien, A. Productivity and selectivity of gas-phase CO_2 electroreduction to methane at copper nanoparticle-based electrodes. *Energy Technol.* **5**, 922-928 (2017).

19. Manthiram, K., Beberwyck, B. J. & Alivisatos, A. P. Enhanced electrochemical methanation of carbon dioxide with a dispersible nanoscale copper catalyst. *J. Am. Chem. Soc.* **136**, 13319–13325 (2014).

20. Koo, Y. et al. Aligned carbon nanotube/copper sheets: a new electrocatalyst for CO_2 reduction to hydrocarbons. *RSC Adv.* **4**, 16362–16367 (2014).

21. Hossain, M. N., Wen, J. & Chen, A. Unique copper and reduced graphene oxide nanocomposite toward the efficient electrochemical reduction of carbon dioxide. *Scientific reports* **7**, 3184 (2017).

22. Lum, Y. et al. Trace levels of copper in carbon materials show significant electrochemical CO_2 reduction activity. *ACS Catal.* **6**, 202–209 (2016).

23. Shinagawa, T., Larrazábal, G. O., Martín, A. J., Krumeich, F. & Pérez-Ramírez, J. Sulfur-modified copper catalysts for the electrochemical reduction of carbon dioxide to formate. *ACS Catal.* **8**, 837-844 (2018).

24. Li, C. W. & Kanan, M. W. CO_2 reduction at low overpotential on Cu electrodes resulting from the reduction of thick Cu_2O films. *J. Am. Chem. Soc.* **134**, 7231-7234 (2012).

25. Qiao, J. L. et al. Highly-active copper oxide/copper electrocatalysts induced from hierarchical copper oxide nanospheres for carbon dioxide reduction reaction. *Electrochimica Acta* **153**, 559-565 (2015).

26. Fan, M. Y., Bai, Z. Y., Zhang, Q., Ma, C. Y., Zhou, X. D. & Qiao, J. L. Aqueous CO_2 reduction on morphology controlled Cu_xO nanocatalysts at low overpotential. *RSC Adv.* **4**, 44583-44591 (2014).

27. Lan, Y. C., Gai, C., Kenis, P. J. A. & Lu, J. X. Electrochemical reduction of carbon dioxide on Cu/CuO core/shell catalysts. *ChemElectroChem* **1**, 1577-1582 (2014).

28. Huan, T. N., Simon, P., Benayad, A., Guetaz, L., Artero, V. & Fontecave, M. Cu/Cu_2O electrodes and CO_2 reduction to formic acid: Effects of organic additives on surface morphology and activity. *Chem. Eur. J.* **22**, 1-8 (2016).

29. Tang, Q. et al. Lattice-hydride mechanism in electrocatalytic CO_2 reduction by structurally precise copper-hydride nanoclusters. *J. Am. Chem. Soc.* **139**, 9728-9736 (2017).

30. Han, Z., Kortlever, R., Chen, H. Y., Peters, J. C. & Agapie, T. CO_2 reduction selective for $C_{\geq 2}$ products on polycrystalline copper with N-substituted pyridinium additives. *ACS Cent. Sci.* **3**, 853-859 (2017).

31. Hori, Y., Murata, A. & Takahashi, R. Formation of hydrocarbons in the electrochemical reduction of carbon dioxide at a copper electrode in aqueous solution. *J. Chem. Soc., Faraday Trans. 1* **85**, 2309-2326 (1989).

32. Kuhl, K. P., Cave, E. R., Abram, D. N. & Jaramillo, T. F. New insights into the electrochemical reduction of carbon dioxide on metallic copper surfaces. *Energy Environ. Sci.* **5**, 7050-7059 (2012).

33. Hori, Y., Takahashi, Koga, I. O. & Hoshi, N. Electrochemical reduction of carbon dioxide at various series of copper single crystal electrodes. *J. Mol. Catal. A: Chem.* **199**, 39-47 (2003).

34. Hahn, C. et al. Engineering Cu surfaces for the electrocatalytic conversion of CO_2: Controlling selectivity toward oxygenates and hydrocarbons. *Proc. Natl. Acad. Sci.* **114**, 5918-5923 (2017).

35. Mistry, H. et al. Highly selective plasma-activated copper catalysts for carbon dioxide reduction to ethylene. *Nat. Commun.* **7**, 12123 (2016).

36. Lum, Y., Yue, B., Lobaccaro, P., Bell, A. T. & Ager, J. W. Optimizing C-C coupling on oxide-derived copper catalysts for electrochemical CO_2 reduction. *J. Phys. Chem. C* **121**, 14191-14203 (2017).

37. Gonçalve, M. R. et al. Electrochemical conversion of CO_2 to C_2 hydrocarbons using different ex situ copper electrodeposits, *Electrocmimica Acta* **102**, 388-392 (2013).

38. Jeon, H. S., Kunze, S., Scholten, F. & Cuenya, B. R. Prism-shaped Cu nanocatalysts for electrochemical CO_2 reduction to ethylene. *ACS Catal.* **8**, 531-535 (2018).

39. Reske, R., Mistry, H., Behafarid, F., Cuenya, B. R. & Strasser, P. Particle size effects in the catalytic electroreduction of CO_2 on Cu nanoparticles. *J. Am. Chem. Soc.* **136**, 6978-6986 (2014).

40. Ma, S., Sadakiyo, M., Luo, R., Heima, M., Yamauchi, M. & Kenis, P. J. A. One-step electrolysis of ethylene and ethanol from CO_2 in an alkaline electrolyzer. *J. Power Sources* **301**, 219-228 (2016).

41. Tang, W. et al. The importance of surface morphology in controlling the selectivity of polycrystalline copper for CO_2 electroreduction, *Phys. Chem. Chem. Phys.* **14**, 76-81 (2012).

42. Ren, D., Wong N. T., Handoko, A. D., Huang, Y. & Yeo, B. S. Mechanistic insights into the enhanced activity and stability of agglomerated Cu nanocrystals for the electrochemical reduction of carbon dioxide to n-Propanol. *J. Phys. Chem. Lett.* **7**, 20-24 (2016).

43. Xie, J. F., Huang, Y. X., Li, W. W., Song, X. N., Xiong, L. & Yu, H. Q. Efficient electrochemical CO_2 reduction on a unique chrysanthemum-like Cu nanoflower electrode and direct observation of carbon deposite. *Electrochim. Acta* **139**, 137–144 (2014).

44. Roberts, F. S., Kuhl, K. P. & Nilsson, A. High selectivity for ethylene from carbon dioxide reduction over copper nanocube electrocatalysts. *Angew Chem. Int. Ed.* **54**, 5179-5182 (2015).

45. Loiudice, A. et al. Tailoring copper nanocrystals towards C_2 products in electrochemical CO_2 reduction. *Angew. Chem. Int. Ed.* **55**, 5789-5792 (2016).

46. Gao, D. F. et al. Plasma-activated copper nanocube catalysts for efficient carbon dioxide electroreduction to hydrocarbons and alcohols. *ACS Nano* **11**, 4825-4831 (2017).

47. Reller, C. et al. Selective electroreduction of CO_2 toward ethylene on nano dendritic copper catalysts at high current density. *Adv. Energy Mater.* **7**, 1602114 (2017).

48. Yang, K. D. et al. Morphology-directed selective production of ethylene or ethane from CO_2 on a Cu mesopore electrode. *Angew. Chem. Int. Ed.* **56**, 796-800 (2017).

49. Chen, C. S., Handoko, A. D., Wan, J. H., Ma, L., Ren, D. & Yeo, B. S. Stable and selective electrochemical reduction of carbon dioxide to ethylene on copper mesocrystals. *Catal. Sci. Technol.* **5**, 161–168 (2015).

50. Yano, H., Tanaka, T., Nakayama, M. & Ogura, K. Selective electrochemical reduction of CO_2 to ethylene at a three-phase interface on copper(I) halide-confined Cu-mesh electrodes in acidic solutions of potassium halides, *J. Electroanal. Chem.* **565**, 287-293 (2004).

51. Kwon, Y., Lum, Y., Clark, E. L., Ager, J. W. & Bell, A. T. CO_2 electroreduction with enhanced ethylene and ethanol selectivity by nanostructuring polycrystalline copper. *ChemElectroChem* **3**, 1012-1019 (2016).

52. Ren, D., Fong, J. & Yeo, B. S. The effects of currents and potentials on the selectivities of copper toward carbon dioxide electroreduction. *Nat. Commun.* **9**, 925 (2018).

53. Luna, P. D. et al. Catalyst electro-redeposition controls morphology and oxidation state for selective carbon dioxide reduction. *Nat. Catal.* **1**, 103-110 (2018).

54. Zhou, Y. S. et al. Dopant-induced electron localization drives CO_2 reduction to C_2 hydrocarbons. *Nat. Chem.* **10**, 974-980 (2018).

55. Jiang, K. et al. Metal ion cycling of Cu foil for selective C-C coupling in electrochemical CO_2 reduction. *Nat. Catal.* **1**, 111-119 (2018).

56. Dutta, A., Rahaman, M., Luedi, N. C., Mohos, M. & Broekmann, P. Morphology matters: Tuning the product distribution of CO_2 electroreduction on oxide-derived Cu foam catalysts. *ACS Catal.* **6**, 3804–3814 (2016).

57. Kas, R., Kortlever, R., Milbrat, A., Koper, M. T. M., Mul, G. & Baltrusaitis, J. Electrochemical CO_2 reduction on Cu_2O-derived copper nanoparticles: controlling

the catalytic selectivity of hydrocarbons. *Phys. Chem. Chem. Phys.* **16**, 12194-12201 (2014).

58. Ren, D., Deng Y., Handoko A. D., Chen C. S., Malkhandi S. & Yeo B. S. Selective Electrochemical Reduction of Carbon Dioxide to Ethylene and Ethanol on Copper(I) Oxide Catalysts. *ACS Catal.* **5**, 2814-2821 (2015).

59. Handoko, A. D. et al. Mechanistic insights into the selective electroreduction of carbon dioxide to ethylene on Cu_2O-derived copper catalysts. *J. Phys. Chem. C* **120**, 20058-20067 (2016).

60. Chi, D. H. et al. Morphology-controlled CuO nanoparticles for electroreduction of CO_2 to ethanol. *RSC Adv.* **4**, 37329-37332 (2014).

61. Kim, D., Lee, S., Ocon, J. D., Jeong, B. Lee, J. K. & Lee, J. Insights into an autonomously formed oxygen-evacuated Cu_2O electrode for the selective production of C_2H_4 from CO_2. *Phys. Chem. Chem. Phys.* **17**, 824-830 (2015).

62. Huang, Y., Handoko, A. D., Hirunsit, P. & Yeo, B. S. Electrochemical reduction of CO_2 using copper single-crystal surfaces: effects of CO* coverage on the selective formation of ethylene. *ACS Catal.* **7**, 1749-1756 (2017).

63. Rahaman, M., Dutta, D., Zanetti, A. & Broekmann, P. Electrochemical reduction of CO_2 into multicarbon alcohols on activated Cu mesh catalysts: An indentical location (IL) study. *ACS Catal.* **7**, 7946-7956 (2018).

64. Song, Y. et al. High-selectivity electrochemical conversion of CO_2 to ethanol using a copper nanoparticle/N-doped graphene electrode. *Chemistry Select* **1**, 6055-6061 (2016).

65. Baturina, O. A. et al. CO_2 electroreduction to hydrocarbons on carbon-supported Cu nanoparticles. *ACS Catal.* **4**, 3682-3695 (2014).

66. Jiao, Y., Zheng, Y., Chen, P., Jaroniec, M. & Qiao, S. Z. Molecular scaffolding strategy with synergistic active centers to facilitate electrocatalytic CO_2 reduction to hydrocarbon/Alcohol. *J. Am. Chem. Soc.* **139**, 18093-18100 (2017).

67. Genovese, C., Ampelli, C., Perathoner, S. & Centi, G. Mechanism of C-C bond formation in the electrocatalytic of CO_2 to acetic acid. A challenging reaction to use renewable energy with chemistry. *Green Chem.* **19**, 2406-2415 (2017).

68. Weng, Z. et al. Self-Cleaning catalyst electrodes for stabilized CO_2 reduction to hydrocarbons. *Angew. Chem. Int. Ed.* 56, 13135-13139 (2017).

69. Ren, D., Ang, B. S. H. & Yeo, B. S. Tuning the selectivity of carbon dioxide electroreduction toward ethanol on oxide-derived Cu_xZn catalyst. *ACS Catal.* **6**, 8239-8247 (2016).

70. Jia, F. L., Yu, X. X. & Zhang, L. Z. Enhanced selectivity for the electrochemical reduction of CO_2 to alcohols in aqueous solution with nanostructured Cu-Ag alloy as catalyst. *J. Power Sources* 252, 85-89 (2014).

71. Clark, E. L., Hahn, C., Jaramillo, T. F. & Bell, A. T. Electrochemical CO_2 reduction over compressively strained CuAg surface alloys with enhanced multi-carbon oxygenate selectivity. *J. Am. Chem. Soc.* **139**, 15848-15857 (2017).

72. Chen, C. S., Wan, J. H. & Yeo, B. S. Electrochemical reduction of carbon dioxide to ethane using nanostructured Cu_2O-derived copper catalyst and Palladium(II) chloride. *J. Phys. Chem. C* **119**, 26875-26882 (2015).

73. Yang, H. P., Yue, Y. N., Qin, S., Wang, H. & Lu, J. X. Selective electrochemical reduction of CO_2 to different alcohol products by an organically doped alloy catalyst. *Green Chem.* **18**, 3216-3220 (2016).

74. Hoang, T. T. H., Ma, S., Gold, J. I., Kenis, P. J. A. & Gewirth, A. A. Nanoporous copper films by additive-controlled electrodeposition: CO_2 reduction catalysis. *ACS Catal.* **7**, 3313-3321 (2017).

75. Weng, Z. et al. Electrochemical CO_2 reduction to hydrocarbons on a heterogeneous molecular Cu catalyst in aqueous solution. *J. Am. Chem. Soc.* **138**, 8076-8079 (2016).

76. Albo, J., Vallejo, D., Beobide, G., Castillo, O., Castaño, P. & Irabien, A. Copper-based metal-organic porous materials for CO_2 electrocatalytic reduction to alcohols. *ChemSusChem* **9**, 1-11 (2016).

77. Zhang, F. Y. et al. Cu overlayers on tetrahexahedral Pd nanocrystals with high-index facets for CO_2 electroreduction to alcohols. *Chem. Commun.* **53**, 8085-8088 (2017).

78. Xie, M. S. et al. Amino acid modified copper electrodes for the enhanced selective electroreduction of carbon dioxide toward hydrocarbons. *Energy Environ. Sci.* **9**, 1687-1695 (2016).

79. Sun, X. F. et al. Design of a Cu (I)/C-doped boron nitride electrocatalyst for efficient conversion of CO_2 into acetic acid. *Green Chem.* 19, 2086-2091 (2017).

80. Dinh, C. T. et al. CO_2 electroreduction to ethylene via hydroxide-mediated copper catalysis at an abrupt interface. *Science* **360**, 783-787 (2018).

81. Zhuang, T. T. et al. Steering post-C-C coupling selectivity enable high efficiency electroreduction of carbon dioxide to multi-carbon alcohols. *Nat. Catal.* **1**, 421-428 (2018).

82. Song, Y. F., Chen, W., Zhao, C. C., Li, S. G., Wei, W. & Sun, Y. H. Metal-free Nitrogen-doped mesoporous carbon for electroreduction of CO_2 to ethanol. *Angew. Chem. Int. Ed.* **56**, 10840-10844 (2017).

83. Kortlever, R. et al. Palladium-gold catalyst for the electrochemical reduction of CO_2 to C_1-C_5 hydrocarbons. *Chem. Commun.* **52**, 10229-10232 (2016).

84. Liu, Y. M., Chen, S., Quan, X. & Yu, H. T. Efficient electrochemical reduction of carbon dioxide to acetate on nitrogen-doped nanodiamond. *J. Am. Chem. Soc.* **137**, 11631-11636 (2015).

85. Torelli, D. A. et al. Nickel-Gallium-catalyzed electrochemical reduction of CO_2 to highly reduced products at low overpotentials. *ACS Catal.* **6**, 2100-2104 (2016).

86. Klahr, B., Gimenez, S., Fabregat-Santiago, F., Bisquert, J. & Hamann, T. W. Photoelectrochemical and impedance spectroscopic investigation of water oxidation with "Co-Pi"-coated hematite electrodes. *J. Am. Chem. Soc.* **134**, 16693-16700

(2012).

87. Mao, Y. Y. et al. Foldable interpenetrated metal-organic frameworks/carbon nanotubes thin film for lithium-sulfur batteries. *Nat. Commun.* **8**, 14628 (2017).

88. Bertheussen, E. et al. Acetaldehyde as an intermediate in the Electroreduction of carbon monoxide to ethanol on oxide-derived copper. *Angew. Chem. Int. Ed.* **55**, 1450-1454 (2016).

89. Zhou, Z. Y., Wang, Q., Lin, J. L. & Sun, S. G. In situ FTIR spectroscopic studies of electrooxidation of ethanol on Pd electrode in alkaline media. *Electrochimica Acta* **55**, 7995-7999 (2010).
