# Supplementary material for: Carbon dioxide electroreduction to C2 products over copper-cuprous oxide derived from electrosynthesized copper complex
Source: Nat Commun. 2019 Aug 26;10:3851. doi: 10.1038/s41467-019-11599-7 (PMC6710288; doi:10.1038/s41467-019-11599-7)
Supplement: Supplementary file 3 — Crystal data of complex-1 [file 41467_2019_11599_MOESM3_ESM.pdf]

Table 1. Crystal data and structure refinement for complex-1.

Identification code	complex-1	
Empirical formula	C20 H44 Cu4 O36	
Formula weight	1114.71	
Temperature	110(2) K	
Wavelength	0.71073 Å	
Crystal system	Monoclinic	
Space group	I 1 2/c 1	
Unit cell dimensions	a = 9.540(4) Å	α= 90 °.
	b = 18.119(5) Å	β= 112.67(3) °.
	c = 12.077(2) Å	γ = 90 °.
Volume	1926.3(11) $Å^3$	
Z	2	
Density (calculated)	1.922 Mg/m^3	
Absorption coefficient	2.299 mm^{-1}	
F(000)	1136	
Crystal size	0.298 x 0.171 x 0.135 mm^3	
Theta range for data collection	2.145 to 27.464 °.	
Index ranges	-12<=h<=12, -23<=k<=23, -14<=l<=15	
Reflections collected	12706	
Independent reflections	2204 [R(int) = 0.0591]	
Completeness to theta = 25.242 °	99.4 %	
Absorption correction	Semi-empirical from equivalents	
Max. and min. transmission	1.0000 and 0.69274	
Refinement method	Full-matrix least-squares on F^2	
Data / restraints / parameters	2204 / 0 / 139	
Goodness-of-fit on F^2	1.104	
Final R indices [I>2sigma(I)]	R1 = 0.0269, wR2 = 0.0726	
R indices (all data)	R1 = 0.0271, wR2 = 0.0728	
Extinction coefficient	n/a	
Largest diff. peak and hole	0.479 and -0.535 e.$Å^{-3}$	

Table 2. Atomic coordinates (x 10^4) and equivalent isotropic displacement parameters ($Å^2$x 10^3) for complex-1. U(eq) is defined as one third of the trace of the orthogonalized U^{ij} tensor.

	x	y	z	U(eq)
Cu1	3615(1)	1902(1)	5296(1)	8(1)
O1	4851(1)	2753(1)	6244(1)	9(1)
O2	2544(1)	3240(1)	5479(1)	11(1)
O3	2497(1)	6083(1)	5720(1)	14(1)
O4	4898(2)	6465(1)	6201(1)	19(1)
O5	2688(1)	1696(1)	6449(1)	13(1)
O6	5598(1)	1190(1)	6350(1)	11(1)
O7	4312(2)	2136(1)	4012(1)	14(1)
C1	3916(2)	3270(1)	6168(1)	8(1)
C2	4481(2)	3959(1)	6902(1)	8(1)
C3	3955(2)	4631(1)	6321(2)	9(1)
C4	4471(2)	5300(1)	6906(2)	10(1)
C5	3932(2)	6013(1)	6228(2)	11(1)
O9	5189(2)	274(1)	1231(2)	39(1)
O8	3068(2)	1390(1)	1922(1)	20(1)

Table 3. Bond lengths [Å] and angles [°] for complex-1.

Cu1-O1	2.0107(13)
Cu1-O3#1	1.9568(13)
Cu1-O5	1.9519(14)
Cu1-O6	2.2382(14)
Cu1-O7	1.9550(13)
O1-C1	1.272(2)
O2-C1	1.253(2)
O3-C5	1.273(2)
O4-C5	1.243(2)
O5-H5A	0.8500
O5-H5B	0.8500
O6-H6A	0.8500
O6-H6B	0.8501
O7-H7A	0.8500
O7-H7B	0.8500
C1-C2	1.505(2)
C2-C2#2	1.402(3)
C2-C3	1.398(2)
C3-H3	0.9500
C3-C4	1.394(2)
C4-C4#2	1.401(3)
C4-C5	1.509(2)
O9-H9A	0.8499
O9-H9B	0.8500
O8-H8A	0.8500
O8-H8B	0.8499
O1-Cu1-O6	86.37(5)
O3#1-Cu1-O1	175.08(5)
O3#1-Cu1-O6	93.14(6)
O5-Cu1-O1	93.89(6)
O5-Cu1-O3#1	91.01(6)
O5-Cu1-O6	90.01(6)
O5-Cu1-O7	173.50(5)

O7-Cu1-O1	88.88(5)
O7-Cu1-O3#1	86.30(6)
O7-Cu1-O6	96.04(6)
C1-O1-Cu1	106.43(11)
C5-O3-Cu1#3	126.88(12)
Cu1-O5-H5A	125.6
Cu1-O5-H5B	118.4
H5A-O5-H5B	109.6
Cu1-O6-H6A	99.0
Cu1-O6-H6B	117.6
H6A-O6-H6B	103.5
Cu1-O7-H7A	123.7
Cu1-O7-H7B	121.8
H7A-O7-H7B	106.6
O1-C1-C2	119.08(14)
O2-C1-O1	122.71(15)
O2-C1-C2	118.18(15)
C2#2-C2-C1	123.88(9)
C3-C2-C1	116.66(14)
C3-C2-C2#2	119.43(10)
C2-C3-H3	119.5
C4-C3-C2	121.01(15)
C4-C3-H3	119.5
C3-C4-C4#2	119.55(10)
C3-C4-C5	119.30(15)
C4#2-C4-C5	121.09(9)
O3-C5-C4	115.15(15)
O4-C5-O3	126.32(16)
O4-C5-C4	118.53(15)
H9A-O9-H9B	86.0
H8A-O8-H8B	104.5

Symmetry transformations used to generate equivalent atoms:

#1 -x+1/2,y-1/2,-z+1   #2 -x+1,y,-z+3/2   #3 -x+1/2,y+1/2,-z+1

Table 4. Anisotropic displacement parameters ($Å^2 x 10^3$) for complex-1. The anisotropic displacement factor exponent takes the form: $-2\pi^2[h^2 a^{*2}U^{11} + ... + 2 h k a^* b^* U^{12}]$

	U^{11}	U^{22}	U^{33}	U^{23}	U^{13}	U^{12}
Cu1	9(1)	6(1)	8(1)	-2(1)	2(1)	-2(1)
O1	10(1)	6(1)	12(1)	-2(1)	5(1)	-1(1)
O2	9(1)	10(1)	12(1)	-2(1)	2(1)	-1(1)
O3	14(1)	9(1)	14(1)	3(1)	0(1)	2(1)
O4	19(1)	10(1)	20(1)	5(1)	0(1)	-4(1)
O5	9(1)	18(1)	14(1)	4(1)	4(1)	3(1)
O6	11(1)	9(1)	13(1)	0(1)	5(1)	0(1)
O7	22(1)	10(1)	10(1)	-1(1)	6(1)	-5(1)
C1	12(1)	6(1)	7(1)	-1(1)	5(1)	-2(1)
C2	8(1)	7(1)	9(1)	-1(1)	3(1)	-1(1)
C3	9(1)	9(1)	7(1)	1(1)	1(1)	1(1)
C4	9(1)	7(1)	10(1)	1(1)	2(1)	0(1)
C5	14(1)	6(1)	9(1)	-1(1)	0(1)	0(1)
O9	76(1)	12(1)	56(1)	-5(1)	54(1)	-6(1)
O8	22(1)	21(1)	12(1)	0(1)	1(1)	2(1)

Table 5. Hydrogen coordinates (x 10^4) and isotropic displacement parameters (Å^{2}x 10 ^{3}) for complex-1.

	x	y	z	U(eq)
H5A	1919	1917	6483	20
H5B	3255	1547	7145	20
H6A	6189	1301	5999	16
H6B	5492	725	6255	16
H7A	3907	1963	3305	22
H7B	4654	2560	3948	22
H3	3235	4631	5516	11
H9A	5844	453	990	59
H9B	5657	507	1880	59
H8A	3126	1531	1269	29
H8B	2121	1356	1758	29

Table 6. Torsion angles [°] for complex-1.

Cu1-O1-C1-O2	-8.78(19)
Cu1-O1-C1-C2	173.32(11)
Cu1#3-O3-C5-O4	2.3(3)
Cu1#3-O3-C5-C4	-176.84(11)
O1-C1-C2-C2#2	-41.7(3)
O1-C1-C2-C3	136.38(16)
O2-C1-C2-C2#2	140.3(2)
O2-C1-C2-C3	-41.6(2)
C1-C2-C3-C4	-176.74(15)
C2#2-C2-C3-C4	1.4(3)
C2-C3-C4-C4#2	-0.2(3)
C2-C3-C4-C5	177.06(15)
C3-C4-C5-O3	53.5(2)
C3-C4-C5-O4	-125.73(18)
C4#2-C4-C5-O3	-129.3(2)
C4#2-C4-C5-O4	51.5(3)

Symmetry transformations used to generate equivalent atoms:

#1 -x+1/2,y-1/2,-z+1 #2 -x+1,y,-z+3/2 #3 -x+1/2,y+1/2,-z+1

Table 7. Hydrogen bonds for complex-1 [Å and °].

D-H...A	d(D-H)	d(H...A)	d(D...A)	<(DHA)
O5-H5A...O1#4	0.85	1.97	2.804(2)	165.5
O5-H5B...O6#2	0.85	1.84	2.689(2)	176.9
O6-H6A...O2#5	0.85	1.84	2.6702(19)	165.0
O6-H6B...O9#6	0.85	1.83	2.675(2)	171.9
O7-H7A...O8	0.85	1.86	2.702(2)	167.8
O7-H7B...O4#7	0.85	1.84	2.6846(19)	171.0
O9-H9A...O3#8	0.85	2.07	2.903(2)	166.0
O9-H9B...O8#9	0.85	2.19	2.996(2)	159.2

Symmetry transformations used to generate equivalent atoms:

#1 -x+1/2,y-1/2,-z+1 #2 -x+1,y,-z+3/2 #3 -x+1/2,y+1/2,-z+1

#4 x-1/2,-y+1/2,z #5 x+1/2,-y+1/2,z #6 x,-y,z+1/2

#7 -x+1,-y+1,-z+1 #8 x+1/2,y-1/2,z-1/2 #9 -x+1,y,-z+1/2
