# Supplementary material for: Carbon dioxide electroreduction to C2 products over copper-cuprous oxide derived from electrosynthesized copper complex
Source: Nat Commun. 2019 Aug 26;10:3851. doi: 10.1038/s41467-019-11599-7 (PMC6710288; doi:10.1038/s41467-019-11599-7)
Supplement: Supplementary file 4 — Crystal data of complex-4 [file 41467_2019_11599_MOESM4_ESM.pdf]

Table 1. Crystal data and structure refinement for complex-4.

Identification code	complex-4	
Empirical formula	C18 H22 Cu O18	
Formula weight	589.89	
Temperature	173.15 K	
Wavelength	0.71073 Å	
Crystal system	Monoclinic	
Space group	P 1 21 1	
Unit cell dimensions	a = 11.7001(3) Å	α= 90 °.
	b = 6.6745(2) Å	β= 95.390(2) °.
	c = 28.4761(6) Å	γ = 90 °.
Volume	2213.93(10) $Å^3$	
Z	4	
Density (calculated)	1.770 Mg/m^3	
Absorption coefficient	1.081 mm^{-1}	
F(000)	1212	
Crystal size	0.185 x 0.041 x 0.027 mm^3	
Theta range for data collection	1.748 to 31.431 °.	
Index ranges	-16<=h<=17, -9<=k<=9, -39<=l<=41	
Reflections collected	21612	
Independent reflections	12670 [R(int) = 0.0449]	
Completeness to theta = 25.242 °	99.8 %	
Absorption correction	Semi-empirical from equivalents	
Max. and min. transmission	1.00000 and 0.52451	
Refinement method	Full-matrix least-squares on F^2	
Data / restraints / parameters	12670 / 37 / 678	
Goodness-of-fit on F^2	1.027	
Final R indices [I>2sigma(I)]	R1 = 0.0734, wR2 = 0.1582	
R indices (all data)	R1 = 0.1119, wR2 = 0.1833	
Absolute structure parameter	0.50(3)	
Extinction coefficient	n/a	
Largest diff. peak and hole	0.838 and -0.711 e.$Å^{-3}$	

Table 2. Atomic coordinates (x 10^4) and equivalent isotropic displacement parameters ($Å^2$x 10^3) for complex-4. U(eq) is defined as one third of the trace of the orthogonalized U^{ij} tensor.

	x	y	z	U(eq)
C1	10263(7)	2568(16)	1093(3)	15(2)
C2	9143(7)	2462(17)	831(3)	18(2)
C3	9074(6)	2675(19)	340(3)	21(2)
C4	10078(7)	2911(18)	112(3)	20(2)
C5	11160(7)	2927(17)	359(3)	20(2)
C6	11246(6)	2718(18)	857(3)	18(2)
C7	10332(7)	2493(17)	1633(3)	15(2)
C8	8107(7)	2190(15)	1075(3)	12(2)
C9	12381(7)	2912(16)	1126(3)	17(2)
C10	9728(7)	2372(17)	3891(3)	18(2)
C11	8756(6)	2464(16)	4139(3)	13(2)
C12	8855(7)	2510(20)	4632(3)	28(3)
C13	9934(9)	2420(20)	4878(3)	36(3)
C14	10941(7)	2244(19)	4635(3)	23(2)
C15	10844(7)	2222(17)	4148(3)	16(2)
C16	7605(7)	2787(15)	3883(3)	15(2)
C17	9649(7)	2344(17)	3353(3)	15(2)
C18	11886(7)	2104(16)	3895(3)	14(2)
Cu1	9908(1)	4157(2)	2491(1)	15(1)
O1	10115(5)	4103(13)	1828(2)	16(1)
O2	10626(5)	978(12)	1838(2)	17(1)
O3	8109(5)	1659(12)	1474(2)	24(2)
O4	7165(5)	2658(14)	811(2)	26(2)
O5	12523(5)	3481(12)	1526(2)	23(2)
O6	13223(5)	2317(14)	867(2)	23(2)
O7	9772(5)	4023(12)	3153(2)	14(1)
O8	9407(5)	788(13)	3146(2)	19(1)
O9	11870(5)	1698(13)	3479(2)	28(2)
O10	12827(5)	2494(14)	4163(2)	23(2)
O11	7460(5)	3351(12)	3476(2)	24(2)
O12	6766(5)	2266(13)	4141(2)	22(2)

O13	8286(6)	3363(17)	2334(2)	48(2)
O14	9276(6)	7404(13)	2385(2)	40(2)
O15	11515(5)	4685(12)	2667(2)	29(2)
C1A	4779(7)	7465(16)	3906(3)	14(2)
C2A	3792(7)	7282(17)	4147(3)	16(2)
C3A	3904(8)	7330(20)	4642(3)	29(2)
C4A	4945(9)	7440(20)	4896(3)	35(3)
C5A	5891(8)	7470(20)	4664(3)	30(3)
C6A	5829(7)	7518(18)	4172(3)	18(2)
C7A	4666(7)	7538(16)	3375(3)	12(2)
C8A	2607(7)	7160(17)	3891(3)	20(2)
C9A	6923(8)	7701(17)	3927(3)	19(2)
C10A	5273(7)	7445(16)	1094(3)	13(2)
C11A	4223(7)	7425(16)	828(3)	13(2)
C12A	4181(8)	7290(18)	335(3)	23(2)
C13A	5135(9)	7130(20)	106(3)	27(2)
C14A	6168(8)	7130(19)	368(3)	25(2)
C15A	6266(7)	7244(16)	860(3)	16(2)
C16A	3138(8)	7731(17)	1064(3)	20(2)
C17A	5372(7)	7556(16)	1628(3)	15(2)
C18A	7454(7)	7230(16)	1130(3)	18(2)
Cu1A	4961(1)	5807(2)	2499(1)	15(1)
O1A	4816(5)	5784(12)	3183(2)	17(1)
O2A	4520(5)	9148(13)	3162(2)	21(1)
O3A	2450(5)	6667(13)	3475(2)	27(2)
O4A	1775(5)	7493(13)	4150(2)	21(2)
O5A	6922(5)	8272(12)	3520(2)	23(2)
O6A	7872(5)	7288(14)	4203(2)	21(2)
O7A	5178(5)	5791(12)	1821(2)	15(1)
O8A	5571(5)	9164(13)	1845(2)	23(2)
O9A	7606(5)	6802(13)	1547(2)	26(2)
O10A	8277(5)	7596(13)	863(2)	22(2)
O11A	3135(5)	8345(11)	1472(2)	20(2)
O12A	2176(5)	7354(14)	790(2)	23(2)
O13A	3350(4)	6793(10)	2349(2)	14(1)
O14A	4737(7)	2624(10)	2469(2)	39(2)

O15A	6664(5)	5942(14)	2659(2)	34(2)
O18	3085(6)	5508(10)	7361(2)	38(2)
O20	2596(6)	467(10)	2534(2)	42(2)
O19	5318(5)	7449(11)	6308(2)	17(1)
O16	9622(5)	2595(12)	8688(2)	19(2)
O17	4660(5)	7468(11)	8689(2)	18(1)
O21	9685(5)	7448(12)	3692(2)	18(2)

Table 3. Bond lengths [Å] and angles [°] for complex-4.

C1-C2	1.447(11)
C1-C6	1.388(11)
C1-C7	1.533(11)
C2-C3	1.398(11)
C2-C8	1.465(11)
C3-H3	0.9500
C3-C4	1.405(11)
C4-H4	0.9500
C4-C5	1.390(12)
C5-H5	0.9500
C5-C6	1.419(11)
C6-C9	1.475(11)
C7-O1	1.247(12)
C7-O2	1.202(12)
C8-O3	1.189(10)
C8-O4	1.312(10)
C9-O5	1.198(11)
C9-O6	1.345(10)
C10-C11	1.396(11)
C10-C15	1.440(11)
C10-C17	1.526(11)
C11-C12	1.397(11)
C11-C16	1.486(11)
C12-H12	0.9500
C12-C13	1.387(13)
C13-H13	0.9500
C13-C14	1.426(12)
C14-H14	0.9500
C14-C15	1.381(11)
C15-C18	1.476(11)
C16-O11	1.216(10)
C16-O12	1.328(10)
C17-O7	1.272(13)
C17-O8	1.215(13)

C18-O9	1.214(10)
C18-O10	1.306(10)
Cu1-O1	1.927(6)
Cu1-O7	1.907(6)
Cu1-O13	1.980(7)
Cu1-O14	2.300(8)
Cu1-O15	1.932(6)
O4-H4A	0.8400
O6-H6	0.8400
O10-H10	0.8400
O12-H12A	0.8400
O13-H13A	0.8843
O13-H13B	0.8856
O14-H14A	0.8500
O14-H14B	0.8501
O15-H15A	0.8853
O15-H15B	0.8849
O15-H15B	0.8849
C1A-C2A	1.403(11)
C1A-C6A	1.382(11)
C1A-C7A	1.508(11)
C2A-C3A	1.404(11)
C2A-C8A	1.507(11)
C3A-H3A	0.9500
C3A-C4A	1.359(13)
C4A-H4AA	0.9500
C4A-C5A	1.340(13)
C5A-H5A	0.9500
C5A-C6A	1.396(11)
C6A-C9A	1.519(12)
C7A-O1A	1.310(12)
C7A-O2A	1.237(12)
C8A-O3A	1.224(11)
C8A-O4A	1.296(10)
C9A-O5A	1.221(11)
C9A-O6A	1.328(10)

C10A-C11A	1.382(11)
C10A-C15A	1.401(11)
C10A-C17A	1.517(11)
C11A-C12A	1.402(11)
C11A-C16A	1.504(12)
C12A-H12B	0.9500
C12A-C13A	1.349(13)
C13A-H13C	0.9500
C13A-C14A	1.360(12)
C14A-H14C	0.9500
C14A-C15A	1.395(11)
C15A-C18A	1.524(11)
C16A-O11A	1.234(11)
C16A-O12A	1.331(10)
C17A-O7A	1.328(13)
C17A-O8A	1.249(13)
C18A-O9A	1.216(11)
C18A-O10A	1.304(10)
Cu1A-O1A	1.972(6)
Cu1A-O7A	1.970(6)
Cu1A-O13A	2.005(5)
Cu1A-O14A	2.141(7)
Cu1A-O15A	2.003(6)
O4A-H4AB	0.8400
O6A-H6A	0.8400
O10A-H10A	0.8400
O12A-H12C	0.8400
O13A-H13D	0.8849
O13A-H13E	0.8851
O14A-H14D	0.8552
O14A-H14E	0.8594
O15A-H15C	0.8743
O15A-H15D	0.8763
O18-H18A	0.8498
O18-H18B	0.8504
O20-H20A	0.8402

O20-H20B	0.8501
O20-H20B	0.8501
O19-H19A	0.8496
O19-H19B	0.8502
O16-H16A	0.8501
O16-H16B	0.8502
O17-H17A	0.8501
O17-H17B	0.8500
O21-H21A	0.8500
O21-H21B	0.8498

C2-C1-C7	118.4(8)
C6-C1-C2	120.4(8)
C6-C1-C7	121.3(7)
C1-C2-C8	120.7(8)
C3-C2-C1	118.3(8)
C3-C2-C8	121.0(7)
C2-C3-H3	119.9
C2-C3-C4	120.1(7)
C4-C3-H3	119.9
C3-C4-H4	119.1
C5-C4-C3	121.8(8)
C5-C4-H4	119.1
C4-C5-H5	120.6
C4-C5-C6	118.8(8)
C6-C5-H5	120.6
C1-C6-C5	120.4(7)
C1-C6-C9	120.2(8)
C5-C6-C9	119.0(7)
O1-C7-C1	115.2(9)
O2-C7-C1	120.0(9)
O2-C7-O1	124.7(8)
O3-C8-C2	124.3(8)
O3-C8-O4	123.0(8)
O4-C8-C2	112.6(8)
O5-C9-C6	124.0(8)

O5-C9-O6	124.9(8)
O6-C9-C6	111.1(8)
C11-C10-C15	119.2(8)
C11-C10-C17	122.3(7)
C15-C10-C17	118.5(8)
C10-C11-C12	121.0(7)
C10-C11-C16	120.0(7)
C12-C11-C16	118.4(7)
C11-C12-H12	120.3
C13-C12-C11	119.5(8)
C13-C12-H12	120.3
C12-C13-H13	119.5
C12-C13-C14	120.9(8)
C14-C13-H13	119.5
C13-C14-H14	120.2
C15-C14-C13	119.6(8)
C15-C14-H14	120.2
C10-C15-C18	120.4(8)
C14-C15-C10	119.7(8)
C14-C15-C18	119.9(7)
O11-C16-C11	123.6(8)
O11-C16-O12	124.4(8)
O12-C16-C11	111.9(7)
O7-C17-C10	116.1(9)
O8-C17-C10	119.2(9)
O8-C17-O7	124.6(8)
O9-C18-C15	123.5(8)
O9-C18-O10	123.4(8)
O10-C18-C15	113.1(7)
O1-Cu1-O13	88.8(3)
O1-Cu1-O14	87.5(3)
O1-Cu1-O15	92.8(2)
O7-Cu1-O1	175.5(4)
O7-Cu1-O13	92.5(3)
O7-Cu1-O14	96.8(3)
O7-Cu1-O15	85.6(2)

O13-Cu1-O14	86.2(3)
O15-Cu1-O13	174.7(4)
O15-Cu1-O14	98.9(3)
C7-O1-Cu1	120.2(7)
C8-O4-H4A	109.5
C9-O6-H6	109.5
C17-O7-Cu1	120.5(6)
C18-O10-H10	109.5
C16-O12-H12A	109.5
Cu1-O13-H13A	112.8
Cu1-O13-H13B	125.4
H13A-O13-H13B	109.9
Cu1-O14-H14A	132.3
Cu1-O14-H14B	103.4
H14A-O14-H14B	116.6
Cu1-O15-H15A	131.1
Cu1-O15-H15B	115.4
H15A-O15-H15B	104.1
H15B-O15-Cu1	115.4
H15B-O15-H15A	104.1
H15B-O15-H15B	0.0
C2A-C1A-C7A	119.7(7)
C6A-C1A-C2A	117.6(8)
C6A-C1A-C7A	122.7(7)
C1A-C2A-C3A	119.1(8)
C1A-C2A-C8A	122.0(7)
C3A-C2A-C8A	118.8(8)
C2A-C3A-H3A	119.0
C4A-C3A-C2A	122.0(8)
C4A-C3A-H3A	119.0
C3A-C4A-H4AA	120.7
C5A-C4A-C3A	118.7(8)
C5A-C4A-H4AA	120.7
C4A-C5A-H5A	119.1
C4A-C5A-C6A	121.8(8)
C6A-C5A-H5A	119.1

C1A-C6A-C5A	120.6(8)
C1A-C6A-C9A	119.5(8)
C5A-C6A-C9A	119.8(8)
O1A-C7A-C1A	112.7(8)
O2A-C7A-C1A	121.0(9)
O2A-C7A-O1A	126.2(8)
O3A-C8A-C2A	122.0(8)
O3A-C8A-O4A	123.0(8)
O4A-C8A-C2A	114.8(8)
O5A-C9A-C6A	122.5(8)
O5A-C9A-O6A	123.5(9)
O6A-C9A-C6A	113.9(8)
C11A-C10A-C15A	118.2(8)
C11A-C10A-C17A	122.1(7)
C15A-C10A-C17A	119.6(7)
C10A-C11A-C12A	119.7(8)
C10A-C11A-C16A	119.9(7)
C12A-C11A-C16A	120.3(7)
C11A-C12A-H12B	118.7
C13A-C12A-C11A	122.6(8)
C13A-C12A-H12B	118.7
C12A-C13A-H13C	121.1
C12A-C13A-C14A	117.7(8)
C14A-C13A-H13C	121.1
C13A-C14A-H14C	118.7
C13A-C14A-C15A	122.5(9)
C15A-C14A-H14C	118.7
C10A-C15A-C18A	121.2(8)
C14A-C15A-C10A	119.3(8)
C14A-C15A-C18A	119.5(8)
O11A-C16A-C11A	122.9(8)
O11A-C16A-O12A	122.4(8)
O12A-C16A-C11A	114.6(8)
O7A-C17A-C10A	111.8(9)
O8A-C17A-C10A	122.1(10)
O8A-C17A-O7A	126.1(8)

O9A-C18A-C15A	122.5(8)
O9A-C18A-O10A	124.3(8)
O10A-C18A-C15A	113.1(8)
O1A-Cu1A-O13A	92.7(2)
O1A-Cu1A-O14A	90.6(3)
O1A-Cu1A-O15A	87.3(2)
O7A-Cu1A-O1A	177.4(2)
O7A-Cu1A-O13A	89.9(2)
O7A-Cu1A-O14A	89.0(3)
O7A-Cu1A-O15A	90.3(2)
O13A-Cu1A-O14A	102.0(3)
O15A-Cu1A-O13A	158.3(4)
O15A-Cu1A-O14A	99.7(4)
C7A-O1A-Cu1A	115.5(6)
C8A-O4A-H4AB	109.5
C9A-O6A-H6A	109.5
C17A-O7A-Cu1A	116.3(6)
C18A-O10A-H10A	109.5
C16A-O12A-H12C	109.5
Cu1A-O13A-H13D	143.5
Cu1A-O13A-H13E	126.1
H13D-O13A-H13E	82.6
Cu1A-O14A-H14D	116.0
Cu1A-O14A-H14E	132.7
H14D-O14A-H14E	101.5
Cu1A-O15A-H15C	110.3
Cu1A-O15A-H15D	112.8
H15C-O15A-H15D	102.9
H18A-O18-H18B	101.0
H20A-O20-H20B	117.2
H20B-O20-H20A	117.2
H20B-O20-H20B	0.0
H19A-O19-H19B	98.6
H16A-O16-H16B	113.4
H17A-O17-H17B	118.5
H21A-O21-H21B	85.6

Symmetry transformations used to generate equivalent atoms:

Table 4. Anisotropic displacement parameters ($Å^2 x 10^3$) for complex-4. The anisotropic displacement factor exponent takes the form: $-2\pi^2[h^2 a^{*2}U^{11} + ... + 2 h k a^* b^* U^{12}]$

	U^{11}	U^{22}	U^{33}	U^{23}	U^{13}	U^{12}
C1	19(4)	17(5)	8(3)	0(3)	-3(3)	2(4)
C2	16(4)	26(5)	13(4)	-4(4)	0(3)	2(4)
C3	5(3)	44(7)	14(4)	-8(4)	3(3)	6(4)
C4	13(4)	36(6)	12(4)	4(4)	3(3)	0(4)
C5	16(4)	28(6)	18(4)	-3(4)	5(3)	0(4)
C6	6(4)	33(6)	17(4)	5(4)	6(3)	5(4)
C7	9(4)	22(5)	13(4)	-9(4)	-1(3)	-2(4)
C8	7(4)	13(5)	17(4)	-6(4)	5(3)	-3(3)
C9	15(4)	20(5)	18(4)	3(4)	6(3)	6(4)
C10	18(4)	19(5)	16(4)	5(4)	-4(3)	-6(4)
C11	7(3)	19(5)	15(4)	-2(3)	3(3)	-3(3)
C12	17(4)	52(7)	16(4)	0(5)	1(3)	-14(5)
C13	24(5)	69(9)	17(5)	-2(5)	8(4)	-1(6)
C14	6(3)	48(7)	15(4)	9(4)	1(3)	1(4)
C15	13(4)	19(5)	17(4)	-1(3)	3(3)	0(4)
C16	15(4)	17(5)	12(4)	-1(4)	0(3)	-6(4)
C17	9(4)	26(5)	10(4)	8(4)	1(3)	3(4)
C18	8(4)	22(5)	13(4)	0(4)	2(3)	-1(4)
Cu1	15(1)	20(1)	10(1)	-1(1)	4(1)	-4(1)
O1	17(3)	19(3)	12(3)	0(3)	9(2)	-7(3)
O2	18(3)	16(3)	17(3)	-1(3)	2(2)	5(3)
O3	16(3)	36(5)	21(3)	5(3)	5(3)	-10(3)
O4	13(3)	41(5)	22(3)	6(4)	-1(3)	10(4)
O5	16(3)	33(4)	19(3)	-5(3)	1(2)	2(3)
O6	10(3)	39(5)	21(3)	2(4)	3(2)	5(3)
O7	13(2)	18(3)	11(3)	-4(3)	3(2)	-2(3)
O8	21(3)	19(3)	17(3)	-2(3)	6(2)	-7(3)
O9	13(3)	47(5)	25(3)	-6(4)	2(3)	11(3)
O10	13(3)	37(4)	20(3)	-4(3)	4(2)	-10(3)
O11	16(3)	36(4)	17(3)	10(3)	-2(3)	-2(3)
O12	12(3)	34(4)	20(3)	-1(3)	3(2)	-4(3)

O13	25(4)	96(7)	23(4)	-3(4)	0(3)	13(4)
O14	47(4)	35(4)	37(4)	-9(3)	5(3)	7(3)
O15	27(3)	48(4)	12(3)	0(3)	4(2)	-15(3)
C1A	10(3)	18(4)	14(3)	2(3)	8(3)	3(3)
C2A	16(4)	22(5)	10(4)	-5(4)	0(3)	5(4)
C3A	21(4)	45(5)	21(4)	-6(4)	10(3)	-6(4)
C4A	19(4)	76(10)	10(5)	-5(5)	-2(4)	8(6)
C5A	29(5)	52(7)	8(4)	3(4)	-1(3)	2(5)
C6A	14(4)	29(6)	11(4)	-4(4)	0(3)	-1(4)
C7A	7(3)	17(4)	12(3)	5(3)	1(3)	3(3)
C8A	16(4)	22(5)	22(5)	-1(4)	11(4)	-6(4)
C9A	17(4)	19(5)	21(4)	-4(4)	-3(3)	0(4)
C10A	9(3)	18(4)	13(3)	1(3)	5(3)	2(3)
C11A	14(3)	13(4)	12(3)	-4(3)	3(3)	3(3)
C12A	25(5)	30(5)	14(4)	-4(4)	-4(3)	9(4)
C13A	31(5)	42(7)	9(4)	5(4)	1(4)	-3(5)
C14A	24(5)	35(6)	15(4)	0(5)	7(4)	2(5)
C15A	21(4)	14(5)	12(4)	6(4)	1(3)	1(4)
C16A	16(4)	27(6)	15(4)	-2(4)	-4(3)	-1(4)
C17A	8(4)	21(5)	16(4)	-6(4)	5(3)	0(4)
C18A	9(4)	18(5)	25(5)	3(4)	2(3)	4(4)
Cu1A	15(1)	21(1)	11(1)	0(1)	2(1)	-2(1)
O1A	25(3)	19(3)	9(3)	-2(3)	2(2)	6(3)
O2A	27(3)	17(3)	19(3)	3(3)	-3(2)	-3(3)
O3A	20(3)	38(5)	22(3)	-5(3)	3(3)	-7(3)
O4A	12(3)	33(4)	19(3)	-1(3)	3(2)	-2(3)
O5A	23(3)	33(5)	13(3)	2(3)	3(2)	2(3)
O6A	8(3)	38(4)	17(3)	3(3)	-1(2)	-1(3)
O7A	19(3)	14(3)	11(3)	5(3)	3(2)	-4(3)
O8A	36(4)	19(3)	13(3)	-4(3)	-1(2)	6(4)
O9A	15(3)	44(5)	21(3)	6(3)	0(3)	2(3)
O10A	10(3)	35(5)	21(3)	3(3)	2(2)	0(3)
O11A	22(3)	28(4)	10(3)	1(3)	-3(2)	-3(3)
O12A	11(3)	41(5)	16(3)	3(3)	1(2)	6(3)
O13A	10(2)	20(3)	14(3)	0(2)	5(2)	9(2)
O14A	77(6)	13(3)	29(4)	-3(3)	22(4)	-2(3)

O15A	9(3)	82(6)	11(3)	5(3)	2(2)	3(3)
O18	53(4)	30(4)	28(3)	-3(3)	-12(3)	24(3)
O20	46(4)	46(5)	32(3)	8(4)	-12(3)	8(4)
O19	20(3)	16(3)	15(3)	0(3)	1(2)	2(3)
O16	13(3)	25(4)	18(3)	0(3)	3(2)	1(3)
O17	17(3)	15(3)	22(3)	2(3)	1(2)	-1(3)
O21	11(3)	29(4)	15(3)	-1(3)	2(2)	2(3)

Table 5. Hydrogen coordinates (x 10^4) and isotropic displacement parameters ($Å^2$x 10 ^{3}) for complex-4.

	x	y	z	U(eq)
H3	8346	2660	162	25
H4	10015	3065	-221	24
H5	11830	3074	198	24
H12	8188	2607	4797	34
H13	10003	2471	5213	43
H14	11674	2144	4808	28
H4A	6596	2504	966	38
H6	13864	2583	1012	35
H10	13394	2391	4004	35
H12A	6169	2911	4051	33
H13A	7800	4354	2368	72
H13B	8028	2598	2093	72
H14A	9409	8464	2546	59
H14B	8600	7268	2251	59
H15A	12064	5116	2499	43
H15B	11675	5253	2946	43
H3A	3231	7284	4805	34
H4AA	5002	7489	5231	42
H5A	6622	7460	4840	36
H12B	3454	7310	157	28
H13C	5087	7029	-228	33
H14C	6848	7047	211	29
H4AB	1209	7961	3982	32
H6A	8436	7277	4041	31
H10A	8898	7747	1033	33
H12C	1631	7213	959	34
H13D	2837	7660	2435	21
H13E	3012	7052	2064	21
H14D	4158	2164	2596	58
H14E	5211	1640	2507	58
H15C	6830	5888	2964	51

H15D	6957	7094	2583	51
H18A	3544	5078	7587	57
H18B	3338	4908	7128	57
H20A	1998	508	2347	63
H20B	2510	427	2827	63
H19A	5296	6421	6483	26
H19B	5163	8333	6507	26
H16A	9690	1535	8528	28
H16B	9352	3581	8524	28
H17A	4377	6352	8593	27
H17B	4511	8504	8521	27
H21A	9633	6384	3527	27
H21B	9234	7959	3473	27

Table 6. Torsion angles [°] for complex-4.

C1-C2-C3-C4	2.3(17)
C1-C2-C8-O3	-15.3(17)
C1-C2-C8-O4	162.8(10)
C1-C6-C9-O5	-20.8(17)
C1-C6-C9-O6	157.6(10)
C1-C7-O1-Cu1	169.4(5)
C2-C1-C6-C5	5.0(17)
C2-C1-C6-C9	177.4(10)
C2-C1-C7-O1	-79.7(12)
C2-C1-C7-O2	103.2(11)
C2-C3-C4-C5	0.5(18)
C3-C2-C8-O3	166.4(11)
C3-C2-C8-O4	-15.6(15)
C3-C4-C5-C6	-0.7(17)
C4-C5-C6-C1	-2.1(17)
C4-C5-C6-C9	-174.6(10)
C5-C6-C9-O5	151.8(11)
C5-C6-C9-O6	-29.9(14)
C6-C1-C2-C3	-5.1(16)
C6-C1-C2-C8	176.5(10)
C6-C1-C7-O1	100.8(11)
C6-C1-C7-O2	-76.2(13)
C7-C1-C2-C3	175.5(11)
C7-C1-C2-C8	-2.9(16)
C7-C1-C6-C5	-175.6(10)
C7-C1-C6-C9	-3.1(16)
C8-C2-C3-C4	-179.3(10)
C10-C11-C12-C13	-1.5(19)
C10-C11-C16-O11	-14.3(16)
C10-C11-C16-O12	161.8(10)
C10-C15-C18-O9	-16.3(17)
C10-C15-C18-O10	163.3(10)
C10-C17-O7-Cu1	171.2(5)
C11-C10-C15-C14	-2.3(16)

C11-C10-C15-C18	179.5(10)
C11-C10-C17-O7	96.4(12)
C11-C10-C17-O8	-79.1(13)
C11-C12-C13-C14	-1(2)
C12-C11-C16-O11	157.8(11)
C12-C11-C16-O12	-26.2(14)
C12-C13-C14-C15	2(2)
C13-C14-C15-C10	-0.1(18)
C13-C14-C15-C18	178.1(11)
C14-C15-C18-O9	165.5(12)
C14-C15-C18-O10	-14.9(15)
C15-C10-C11-C12	3.1(17)
C15-C10-C11-C16	175.0(10)
C15-C10-C17-O7	-85.9(12)
C15-C10-C17-O8	98.6(12)
C16-C11-C12-C13	-173.5(11)
C17-C10-C11-C12	-179.2(11)
C17-C10-C11-C16	-7.4(16)
C17-C10-C15-C14	180.0(11)
C17-C10-C15-C18	1.7(16)
O2-C7-O1-Cu1	-13.7(12)
O8-C17-O7-Cu1	-13.6(12)
C1A-C2A-C3A-C4A	-4(2)
C1A-C2A-C8A-O3A	20.4(17)
C1A-C2A-C8A-O4A	-163.7(11)
C1A-C6A-C9A-O5A	17.7(17)
C1A-C6A-C9A-O6A	-165.6(10)
C1A-C7A-O1A-Cu1A	-169.5(5)
C2A-C1A-C6A-C5A	-2.5(17)
C2A-C1A-C6A-C9A	179.6(10)
C2A-C1A-C7A-O1A	-96.0(11)
C2A-C1A-C7A-O2A	88.3(12)
C2A-C3A-C4A-C5A	-1(2)
C3A-C2A-C8A-O3A	-163.3(12)
C3A-C2A-C8A-O4A	12.6(16)
C3A-C4A-C5A-C6A	4(2)

C4A-C5A-C6A-C1A	-2(2)
C4A-C5A-C6A-C9A	175.7(13)
C5A-C6A-C9A-O5A	-160.2(12)
C5A-C6A-C9A-O6A	16.4(16)
C6A-C1A-C2A-C3A	5.2(16)
C6A-C1A-C2A-C8A	-178.5(11)
C6A-C1A-C7A-O1A	82.2(13)
C6A-C1A-C7A-O2A	-93.4(12)
C7A-C1A-C2A-C3A	-176.4(11)
C7A-C1A-C2A-C8A	-0.1(16)
C7A-C1A-C6A-C5A	179.2(12)
C7A-C1A-C6A-C9A	1.3(17)
C8A-C2A-C3A-C4A	179.9(12)
C10A-C11A-C12A-C13A	1.5(18)
C10A-C11A-C16A-O11A	12.9(17)
C10A-C11A-C16A-O12A	-169.4(10)
C10A-C15A-C18A-O9A	22.2(16)
C10A-C15A-C18A-O10A	-161.8(10)
C10A-C17A-O7A-Cu1A	-167.8(5)
C11A-C10A-C15A-C14A	3.1(16)
C11A-C10A-C15A-C18A	-179.3(10)
C11A-C10A-C17A-O7A	78.5(12)
C11A-C10A-C17A-O8A	-99.1(12)
C11A-C12A-C13A-C14A	-0.7(19)
C12A-C11A-C16A-O11A	-161.6(11)
C12A-C11A-C16A-O12A	16.1(15)
C12A-C13A-C14A-C15A	1.2(19)
C13A-C14A-C15A-C10A	-2.5(18)
C13A-C14A-C15A-C18A	179.9(11)
C14A-C15A-C18A-O9A	-160.3(11)
C14A-C15A-C18A-O10A	15.7(15)
C15A-C10A-C11A-C12A	-2.6(16)
C15A-C10A-C11A-C16A	-177.2(10)
C15A-C10A-C17A-O7A	-97.8(11)
C15A-C10A-C17A-O8A	84.6(12)
C16A-C11A-C12A-C13A	176.1(11)

C17A-C10A-C11A-C12A	-179.0(11)
C17A-C10A-C11A-C16A	6.4(16)
C17A-C10A-C15A-C14A	179.6(11)
C17A-C10A-C15A-C18A	-2.9(15)
O2A-C7A-O1A-Cu1A	5.9(11)
O8A-C17A-O7A-Cu1A	9.6(12)

Symmetry transformations used to generate equivalent atoms:

Table 7. Hydrogen bonds for complex-4 [Å and °].

D-H...A	d(D-H)	d(H...A)	d(D...A)	<(DHA)
O4-H4A...O17#1	0.84	1.84	2.679(8)	173.7
O6-H6...O17#2	0.84	1.85	2.675(8)	165.2
O10-H10...O19#2	0.84	1.82	2.657(8)	173.2
O12-H12A...O19#1	0.84	1.96	2.647(8)	138.7
O13-H13A...O15A	0.88	1.94	2.784(10)	158.2
O13-H13B...O3	0.89	1.88	2.690(10)	151.1
O14-H14A...O8#3	0.85	2.31	3.123(11)	161.2
O14-H14B...O9A	0.85	2.24	2.967(10)	143.1
O15-H15A...O13A#4	0.89	1.95	2.789(8)	156.6
O15-H15B...O3A#4	0.88	1.93	2.788(9)	162.8
O4A-H4AB...O21#5	0.84	1.92	2.662(8)	145.8
O6A-H6A...O21	0.84	1.85	2.684(8)	175.9
O10A-H10A...O16#6	0.84	1.84	2.662(8)	166.0
O12A-H12C...O16#7	0.84	1.87	2.691(8)	165.6
O13A-H13D...O20#3	0.88	1.92	2.675(9)	142.4
O13A-H13E...O11A	0.89	1.91	2.691(8)	146.3
O14A-H14D...O2A#8	0.86	2.59	3.072(10)	117.0
O14A-H14D...O20	0.86	2.14	2.910(11)	149.1
O14A-H14E...O8A#8	0.86	2.57	3.125(10)	123.1
O14A-H14E...O18#1	0.86	2.13	2.914(11)	151.0
O15A-H15C...O11	0.87	2.31	2.977(10)	133.3
O15A-H15C...O5A	0.87	2.24	2.896(10)	131.7
O15A-H15D...O18#7	0.88	2.29	3.063(11)	147.8
O18-H18A...O8A#1	0.85	1.93	2.778(9)	172.6
O18-H18B...O5A#1	0.85	2.14	2.917(9)	151.6
O20-H20A...O2#5	0.84	2.08	2.917(8)	171.5
O20-H20B...O9#5	0.85	2.23	3.009(10)	152.1
O19-H19A...O2A#1	0.85	1.83	2.668(11)	171.1
O19-H19B...O1A#7	0.85	1.86	2.669(10)	159.1
O16-H16A...O1#2	0.85	1.94	2.788(11)	178.4
O16-H16B...O2#6	0.85	1.91	2.710(11)	157.5
O17-H17A...O8A#1	0.85	1.93	2.679(11)	147.1

O17-H17B...O7A#7	0.85	1.86	2.668(10)	157.1
O21-H21A...O7	0.85	1.92	2.761(10)	171.0
O21-H21B...O8#3	0.85	2.12	2.721(11)	126.9

Symmetry transformations used to generate equivalent atoms:

#1 -x+1,y-1/2,-z+1 #2 -x+2,y-1/2,-z+1 #3 x,y+1,z

#4 x+1,y,z #5 x-1,y,z #6 -x+2,y+1/2,-z+1

#7 -x+1,y+1/2,-z+1 #8 x,y-1,z
