# Supplementary material for: Carbon dioxide electroreduction to C2 products over copper-cuprous oxide derived from electrosynthesized copper complex
Source: Nat Commun. 2019 Aug 26;10:3851. doi: 10.1038/s41467-019-11599-7 (PMC6710288; doi:10.1038/s41467-019-11599-7)
Supplement: Supplementary file 5 — Crystal data of complex-5 [file 41467_2019_11599_MOESM5_ESM.pdf]

Table 1. Crystal data and structure refinement for complex-5.

Identification code	complex-5	
Empirical formula	C14 H14 Cu N2 O11	
Formula weight	449.81	
Temperature	173.15 K	
Wavelength	0.71073 Å	
Crystal system	Monoclinic	
Space group	P 1 c 1	
Unit cell dimensions	a = 13.395(3) Å	α= 90 °.
	b = 10.139(2) Å	β= 114.442(2) °.
	c = 13.836(3) Å	γ = 90 °.
Volume	1710.8(6) $Å^3$	
Z	4	
Density (calculated)	1.746 Mg/m^3	
Absorption coefficient	1.344 mm^{-1}	
F(000)	916	
Crystal size	0.174 x 0.125 x 0.067 mm^3	
Theta range for data collection	1.670 to 27.489 °.	
Index ranges	-17<=h<=17, -13<=k<=13, -17<=l<=17	
Reflections collected	13009	
Independent reflections	7232 [R(int) = 0.0313]	
Completeness to theta = 25.242 °	99.5 %	
Absorption correction	Semi-empirical from equivalents	
Max. and min. transmission	1.00000 and 0.72748	
Refinement method	Full-matrix least-squares on F^2	
Data / restraints / parameters	7232 / 2 / 509	
Goodness-of-fit on F^2	1.064	
Final R indices [I>2sigma(I)]	R1 = 0.0397, wR2 = 0.0940	
R indices (all data)	R1 = 0.0417, wR2 = 0.0959	
Absolute structure parameter	0.019(8)	
Extinction coefficient	n/a	
Largest diff. peak and hole	0.408 and -0.432 e.$Å^{-3}$	

Table 2. Atomic coordinates (x 10^4) and equivalent isotropic displacement parameters ($Å^2$x 10^3) for complex-5. U(eq) is defined as one third of the trace of the orthogonalized U^{ij} tensor.

	x	y	z	U(eq)
C1	9794(5)	2231(6)	8264(4)	28(1)
C2	10863(4)	2698(6)	8751(4)	31(1)
C3	11058(4)	4010(6)	8664(4)	36(1)
C4	10197(4)	4843(6)	8102(4)	33(1)
C5	9146(4)	4314(5)	7645(4)	29(1)
C6	9509(5)	812(6)	8309(4)	33(1)
C7	8156(4)	5124(6)	7013(4)	32(1)
C8	5408(4)	1566(5)	7059(4)	27(1)
C9	4394(5)	928(6)	6677(5)	35(1)
C10	3991(5)	421(5)	5663(5)	35(1)
C11	4572(5)	529(5)	5042(4)	33(1)
C12	5585(4)	1154(5)	5475(4)	28(1)
C13	6025(4)	2185(6)	8132(4)	30(1)
C14	6358(5)	1387(5)	4960(4)	31(1)
Cu1	7401(1)	2383(1)	7054(1)	29(1)
N1	8945(4)	3035(5)	7716(3)	27(1)
N2	5958(3)	1653(4)	6447(3)	26(1)
O1	8552(3)	457(4)	7882(3)	37(1)
O2	10362(4)	90(4)	8825(4)	45(1)
O3	8387(3)	6321(4)	6863(3)	42(1)
O4	7233(3)	4627(4)	6676(3)	39(1)
O5	6981(3)	2600(4)	8295(3)	35(1)
O6	5579(3)	2262(4)	8760(3)	40(1)
O7	7291(3)	1941(4)	5577(3)	33(1)
O8	6101(4)	1054(5)	4039(3)	47(1)
C15	4411(4)	3502(5)	2429(4)	24(1)
C16	5431(4)	4107(5)	2925(4)	30(1)
C17	5776(4)	4539(5)	3963(4)	30(1)
C18	5090(4)	4350(5)	4487(4)	28(1)
C19	4084(4)	3740(5)	3943(4)	23(1)
C20	3942(4)	3004(5)	1313(4)	24(1)

C21	3257(4)	3498(5)	4397(4)	26(1)
C22	57(5)	2418(5)	905(4)	24(1)
C23	-972(4)	1854(6)	368(4)	32(1)
C24	-1083(4)	504(6)	456(4)	32(1)
C25	-182(4)	-262(5)	1045(4)	30(1)
C26	831(4)	355(5)	1559(4)	23(1)
C27	359(4)	3844(5)	909(4)	29(1)
C28	1924(4)	-261(5)	2235(4)	24(1)
Cu2	2302(1)	2476(1)	2189(1)	23(1)
N3	3756(3)	3328(4)	2933(3)	24(1)
N4	905(3)	1662(4)	1475(3)	22(1)
O9	3024(3)	2499(3)	928(3)	27(1)
O10	4568(3)	3174(4)	818(3)	32(1)
O11	3581(3)	3885(4)	5389(3)	33(1)
O12	2379(3)	2994(4)	3870(3)	32(1)
O13	1980(3)	-1475(3)	2357(3)	32(1)
O14	2720(3)	540(3)	2615(3)	28(1)
O15	-338(4)	4640(4)	382(4)	45(1)
O16	1386(3)	4109(3)	1480(3)	28(1)
O19	6874(4)	7797(5)	5836(4)	56(1)
O21	3683(3)	7185(4)	3849(3)	39(1)
O20	6707(5)	8852(6)	7422(4)	63(1)
O17	10119(4)	2255(4)	4136(4)	55(1)
O18	8440(5)	3162(5)	4507(5)	85(2)
O22	1992(3)	3512(4)	5973(3)	38(1)

Table 3. Bond lengths [Å] and angles [°] for complex-5.

C1-C2	1.389(8)
C1-C6	1.497(8)
C1-N1	1.349(7)
C2-H2	0.9500
C2-C3	1.370(9)
C3-H3	0.9500
C3-C4	1.380(8)
C4-H4	0.9500
C4-C5	1.390(7)
C5-C7	1.496(7)
C5-N1	1.337(7)
C6-O1	1.223(7)
C6-O2	1.294(7)
C7-O3	1.290(7)
C7-O4	1.235(6)
C8-C9	1.396(7)
C8-C13	1.505(7)
C8-N2	1.335(7)
C9-H9	0.9500
C9-C10	1.377(8)
C10-H10	0.9500
C10-C11	1.383(8)
C11-H11	0.9500
C11-C12	1.389(7)
C12-C14	1.499(8)
C12-N2	1.326(6)
C13-O5	1.275(7)
C13-O6	1.246(7)
C14-O7	1.312(7)
C14-O8	1.221(6)
Cu1-N1	1.996(4)
Cu1-N2	1.910(4)
Cu1-O4	2.324(4)
Cu1-O5	2.029(5)

Cu1-O7	2.037(4)
O2-H2A	0.8200
O3-H3A	0.8200
C15-C16	1.392(7)
C15-C20	1.494(7)
C15-N3	1.339(6)
C16-H16	0.9500
C16-C17	1.385(8)
C17-H17	0.9500
C17-C18	1.399(8)
C18-H18	0.9500
C18-C19	1.388(7)
C19-C21	1.504(7)
C19-N3	1.346(6)
C20-O9	1.230(6)
C20-O10	1.296(6)
C21-O11	1.316(6)
C21-O12	1.211(6)
C22-C23	1.389(8)
C22-C27	1.501(7)
C22-N4	1.326(7)
C23-H23	0.9500
C23-C24	1.388(8)
C24-H24	0.9500
C24-C25	1.382(8)
C25-H25	0.9500
C25-C26	1.393(7)
C26-C28	1.509(7)
C26-N4	1.337(6)
C27-O15	1.221(6)
C27-O16	1.300(6)
C28-O13	1.240(6)
C28-O14	1.269(6)
Cu2-N3	1.985(4)
Cu2-N4	1.905(4)
Cu2-O9	2.320(4)

Cu2-O12	2.345(4)
Cu2-O14	2.059(4)
Cu2-O16	2.053(3)
O10-H10A	0.8200
O11-H11A	0.8400
O19-H19A	0.8500
O19-H19B	0.8498
O21-H21A	0.8500
O21-H21B	0.8499
O20-H20A	0.8496
O20-H20B	0.8497
O17-H17A	0.8499
O17-H17B	0.8499
O18-H18A	0.8501
O18-H18B	0.8501
O22-H22A	0.8495
O22-H22B	0.8498

C2-C1-C6	122.4(5)
N1-C1-C2	121.8(5)
N1-C1-C6	115.8(5)
C1-C2-H2	120.5
C3-C2-C1	118.9(5)
C3-C2-H2	120.5
C2-C3-H3	120.1
C2-C3-C4	119.9(5)
C4-C3-H3	120.1
C3-C4-H4	120.9
C3-C4-C5	118.2(5)
C5-C4-H4	120.9
C4-C5-C7	122.7(5)
N1-C5-C4	122.5(5)
N1-C5-C7	114.8(4)
O1-C6-C1	119.8(5)
O1-C6-O2	127.5(6)
O2-C6-C1	112.7(5)

O3-C7-C5	113.4(4)
O4-C7-C5	120.3(5)
O4-C7-O3	126.3(5)
C9-C8-C13	128.2(5)
N2-C8-C9	119.8(5)
N2-C8-C13	112.1(4)
C8-C9-H9	121.1
C10-C9-C8	117.9(5)
C10-C9-H9	121.1
C9-C10-H10	119.3
C9-C10-C11	121.4(5)
C11-C10-H10	119.3
C10-C11-H11	121.0
C10-C11-C12	117.9(5)
C12-C11-H11	121.0
C11-C12-C14	127.6(5)
N2-C12-C11	120.2(5)
N2-C12-C14	112.2(4)
O5-C13-C8	114.5(5)
O6-C13-C8	119.2(5)
O6-C13-O5	126.3(5)
O7-C14-C12	114.6(4)
O8-C14-C12	120.0(5)
O8-C14-O7	125.4(6)
N1-Cu1-O4	76.40(16)
N1-Cu1-O5	99.99(16)
N1-Cu1-O7	99.50(17)
N2-Cu1-N1	176.49(18)
N2-Cu1-O4	107.11(16)
N2-Cu1-O5	80.14(17)
N2-Cu1-O7	80.41(17)
O5-Cu1-O4	92.85(16)
O5-Cu1-O7	160.51(16)
O7-Cu1-O4	91.69(16)
C1-N1-Cu1	122.0(4)
C5-N1-C1	118.6(5)

C5-N1-Cu1	119.3(4)
C8-N2-Cu1	118.1(3)
C12-N2-C8	122.9(4)
C12-N2-Cu1	118.8(4)
C6-O2-H2A	99.9
C7-O3-H3A	111.5
C7-O4-Cu1	109.0(3)
C13-O5-Cu1	115.1(4)
C14-O7-Cu1	113.7(3)
C16-C15-C20	124.5(5)
N3-C15-C16	121.5(5)
N3-C15-C20	114.0(4)
C15-C16-H16	120.4
C17-C16-C15	119.3(5)
C17-C16-H16	120.4
C16-C17-H17	120.5
C16-C17-C18	119.0(5)
C18-C17-H17	120.5
C17-C18-H18	120.7
C19-C18-C17	118.5(5)
C19-C18-H18	120.7
C18-C19-C21	124.3(5)
N3-C19-C18	121.9(5)
N3-C19-C21	113.7(4)
O9-C20-C15	120.4(5)
O9-C20-O10	125.2(5)
O10-C20-C15	114.4(4)
O11-C21-C19	113.9(4)
O12-C21-C19	121.2(5)
O12-C21-O11	125.0(5)
C23-C22-C27	127.2(5)
N4-C22-C23	119.7(4)
N4-C22-C27	113.1(5)
C22-C23-H23	120.7
C24-C23-C22	118.6(5)
C24-C23-H23	120.7

C23-C24-H24	119.8
C25-C24-C23	120.4(5)
C25-C24-H24	119.8
C24-C25-H25	120.8
C24-C25-C26	118.5(5)
C26-C25-H25	120.8
C25-C26-C28	128.4(4)
N4-C26-C25	119.5(5)
N4-C26-C28	112.1(4)
O15-C27-C22	119.7(5)
O15-C27-O16	125.6(5)
O16-C27-C22	114.7(4)
O13-C28-C26	119.0(4)
O13-C28-O14	125.8(5)
O14-C28-C26	115.2(4)
N3-Cu2-O9	76.14(16)
N3-Cu2-O12	75.95(15)
N3-Cu2-O14	100.06(16)
N3-Cu2-O16	99.23(15)
N4-Cu2-N3	179.83(19)
N4-Cu2-O9	103.92(15)
N4-Cu2-O12	103.98(16)
N4-Cu2-O14	80.10(16)
N4-Cu2-O16	80.62(16)
O9-Cu2-O12	152.05(13)
O14-Cu2-O9	94.39(14)
O14-Cu2-O12	91.98(14)
O16-Cu2-O9	89.15(14)
O16-Cu2-O12	93.74(14)
O16-Cu2-O14	160.68(15)
C15-N3-C19	119.8(4)
C15-N3-Cu2	120.1(3)
C19-N3-Cu2	120.2(3)
C22-N4-C26	123.3(4)
C22-N4-Cu2	118.3(3)
C26-N4-Cu2	118.5(3)

C20-O9-Cu2	109.3(3)
C20-O10-H10A	102.8
C21-O11-H11A	109.5
C21-O12-Cu2	109.0(3)
C28-O14-Cu2	114.2(3)
C27-O16-Cu2	113.4(3)
H19A-O19-H19B	103.7
H21A-O21-H21B	91.2
H20A-O20-H20B	104.5
H17A-O17-H17B	97.9
H18A-O18-H18B	113.5
H22A-O22-H22B	125.6

Symmetry transformations used to generate equivalent atoms:

Table 4. Anisotropic displacement parameters ($Å^2$x 10^3) for complex-5. The anisotropic displacement factor exponent takes the form: $-2\pi^2$[h^2 $a^{*2}U^{11}$ + ... + 2 h k a* b* U^{12}]

	U^{11}	U^{22}	U^{33}	U^{23}	U^{13}	U^{12}
C1	21(3)	43(3)	22(2)	-3(2)	10(2)	3(2)
C2	19(2)	49(3)	23(2)	-1(2)	6(2)	5(2)
C3	17(2)	57(4)	31(3)	-6(3)	7(2)	-5(2)
C4	29(3)	43(3)	28(3)	-4(2)	12(2)	-8(2)
C5	24(3)	41(3)	23(2)	-2(2)	10(2)	-1(2)
C6	31(3)	43(3)	26(3)	-1(2)	14(2)	2(2)
C7	28(3)	38(3)	26(2)	0(2)	8(2)	-3(2)
C8	20(2)	29(2)	31(3)	3(2)	10(2)	1(2)
C9	28(3)	36(3)	39(3)	6(2)	10(2)	-2(2)
C10	28(3)	31(3)	40(3)	3(2)	6(2)	-10(2)
C11	32(3)	26(2)	30(3)	-2(2)	2(2)	-5(2)
C12	24(3)	28(2)	25(2)	-1(2)	4(2)	-3(2)
C13	23(3)	38(3)	28(3)	4(2)	12(2)	4(2)
C14	34(3)	33(3)	23(2)	-1(2)	8(2)	4(2)
Cu1	18(1)	40(1)	26(1)	-4(1)	6(1)	-4(1)
N1	21(2)	40(2)	20(2)	-2(2)	9(2)	-1(2)
N2	19(2)	30(2)	23(2)	-1(2)	3(2)	2(2)
O1	33(2)	39(2)	37(2)	-3(2)	11(2)	-3(2)
O2	41(2)	42(2)	47(2)	6(2)	14(2)	10(2)
O3	32(2)	45(2)	43(2)	11(2)	10(2)	0(2)
O4	20(2)	47(2)	39(2)	-2(2)	1(2)	1(2)
O5	21(2)	54(2)	27(2)	-6(2)	7(2)	-5(2)
O6	28(2)	62(3)	30(2)	0(2)	11(2)	1(2)
O7	29(2)	42(2)	29(2)	-3(2)	14(2)	-1(2)
O8	57(3)	53(3)	30(2)	-9(2)	18(2)	-3(2)
C15	22(2)	22(2)	23(2)	0(2)	5(2)	1(2)
C16	21(2)	30(3)	37(3)	0(2)	11(2)	-2(2)
C17	25(3)	27(2)	32(3)	-3(2)	4(2)	-4(2)
C18	22(3)	28(2)	27(2)	-5(2)	4(2)	0(2)
C19	22(2)	23(2)	22(2)	3(2)	6(2)	4(2)
C20	23(2)	22(2)	25(2)	3(2)	9(2)	4(2)

C21	27(3)	22(2)	30(3)	1(2)	11(2)	5(2)
C22	21(3)	29(3)	23(2)	6(2)	12(2)	6(2)
C23	21(2)	48(3)	25(2)	5(2)	8(2)	4(2)
C24	21(2)	48(3)	26(3)	-7(2)	6(2)	-10(2)
C25	30(3)	34(3)	25(2)	-1(2)	11(2)	-6(2)
C26	25(2)	22(2)	24(2)	-1(2)	12(2)	-1(2)
C27	30(3)	32(3)	26(3)	3(2)	13(2)	4(2)
C28	26(2)	28(2)	18(2)	-1(2)	7(2)	1(2)
Cu2	18(1)	23(1)	23(1)	-1(1)	5(1)	-1(1)
N3	24(2)	21(2)	25(2)	-3(2)	9(2)	-3(2)
N4	17(2)	27(2)	21(2)	1(2)	7(2)	1(2)
O9	21(2)	38(2)	24(2)	-7(1)	12(2)	-5(1)
O10	26(2)	45(2)	28(2)	-3(2)	13(2)	-4(2)
O11	36(2)	39(2)	24(2)	-6(2)	12(2)	-5(2)
O12	27(2)	37(2)	29(2)	-5(2)	10(2)	-2(2)
O13	33(2)	25(2)	36(2)	2(2)	11(2)	2(2)
O14	23(2)	27(2)	30(2)	0(1)	7(1)	1(1)
O15	37(2)	40(2)	55(3)	20(2)	16(2)	15(2)
O16	27(2)	26(2)	32(2)	2(1)	11(2)	0(1)
O19	40(3)	88(4)	34(2)	8(2)	9(2)	22(2)
O21	28(2)	54(2)	34(2)	11(2)	14(2)	8(2)
O20	62(3)	74(4)	62(3)	-16(3)	35(3)	-19(3)
O17	50(3)	43(2)	63(3)	3(2)	14(2)	-3(2)
O18	108(5)	60(3)	126(5)	-40(4)	87(5)	-41(3)
O22	54(3)	33(2)	41(2)	7(2)	32(2)	8(2)

Table 5. Hydrogen coordinates (x 10^4) and isotropic displacement parameters (Å^{2}x 10 ^{3}) for complex-5.

	x	y	z	U(eq)
H2	11449	2117	9138	38
H3	11784	4345	8990	43
H4	10320	5753	8030	40
H9	3994	846	7103	42
H10	3301	-12	5386	42
H11	4287	187	4340	39
H2A	10060	-605	8851	67
H3A	7831	6770	6586	63
H16	5886	4222	2556	36
H17	6468	4957	4314	37
H18	5309	4633	5200	34
H23	-1585	2382	-49	38
H24	-1784	104	109	39
H25	-252	-1189	1098	36
H10A	4128	3236	196	48
H11A	3065	3796	5578	49
H19A	6403	7818	5194	84
H19B	6551	8175	6174	84
H21A	3224	7810	3604	58
H21B	4148	7592	3689	58
H20A	7366	8655	7824	94
H20B	6351	8694	7793	94
H17A	9758	2934	4159	82
H17B	10679	2381	4714	82
H18A	8690	3933	4706	128
H18B	8107	2837	4857	128
H22A	1958	2770	6242	58
H22B	1741	4235	6091	58

Table 6. Torsion angles [°] for complex-5.

C1-C2-C3-C4	0.2(8)
C2-C1-C6-O1	178.7(5)
C2-C1-C6-O2	-1.3(8)
C2-C1-N1-C5	0.0(8)
C2-C1-N1-Cu1	-178.0(4)
C2-C3-C4-C5	0.5(8)
C3-C4-C5-C7	-179.6(5)
C3-C4-C5-N1	-1.0(8)
C4-C5-C7-O3	5.5(7)
C4-C5-C7-O4	-175.7(5)
C4-C5-N1-C1	0.8(7)
C4-C5-N1-Cu1	178.8(4)
C5-C7-O4-Cu1	-5.3(6)
C6-C1-C2-C3	179.3(5)
C6-C1-N1-C5	-179.8(4)
C6-C1-N1-Cu1	2.2(6)
C7-C5-N1-C1	179.4(5)
C7-C5-N1-Cu1	-2.5(6)
C8-C9-C10-C11	0.4(8)
C8-C13-O5-Cu1	2.2(6)
C9-C8-C13-O5	174.2(5)
C9-C8-C13-O6	-6.5(8)
C9-C8-N2-C12	0.0(8)
C9-C8-N2-Cu1	-174.1(4)
C9-C10-C11-C12	0.7(8)
C10-C11-C12-C14	180.0(5)
C10-C11-C12-N2	-1.5(8)
C11-C12-C14-O7	-176.4(5)
C11-C12-C14-O8	2.8(9)
C11-C12-N2-C8	1.2(8)
C11-C12-N2-Cu1	175.3(4)
C12-C14-O7-Cu1	-1.9(6)
C13-C8-C9-C10	-179.3(5)
C13-C8-N2-C12	178.7(5)

C13-C8-N2-Cu1	4.7(6)
C14-C12-N2-C8	179.9(4)
C14-C12-N2-Cu1	-6.0(6)
N1-C1-C2-C3	-0.5(8)
N1-C1-C6-O1	-1.5(8)
N1-C1-C6-O2	178.6(5)
N1-C5-C7-O3	-173.2(5)
N1-C5-C7-O4	5.6(7)
N2-C8-C9-C10	-0.8(8)
N2-C8-C13-O5	-4.4(7)
N2-C8-C13-O6	174.9(5)
N2-C12-C14-O7	5.0(7)
N2-C12-C14-O8	-175.7(5)
O3-C7-O4-Cu1	173.3(5)
O6-C13-O5-Cu1	-177.0(5)
O8-C14-O7-Cu1	178.9(5)
C15-C16-C17-C18	0.3(8)
C15-C20-O9-Cu2	-0.8(5)
C16-C15-C20-O9	-178.8(5)
C16-C15-C20-O10	0.1(7)
C16-C15-N3-C19	-0.2(7)
C16-C15-N3-Cu2	179.5(4)
C16-C17-C18-C19	-0.3(7)
C17-C18-C19-C21	-178.7(5)
C17-C18-C19-N3	0.1(7)
C18-C19-C21-O11	-1.9(7)
C18-C19-C21-O12	177.8(5)
C18-C19-N3-C15	0.2(7)
C18-C19-N3-Cu2	-179.5(4)
C19-C21-O12-Cu2	1.8(5)
C20-C15-C16-C17	179.5(5)
C20-C15-N3-C19	-179.8(4)
C20-C15-N3-Cu2	-0.1(5)
C21-C19-N3-C15	179.1(4)
C21-C19-N3-Cu2	-0.5(5)
C22-C23-C24-C25	-1.5(8)

C22-C27-O16-Cu2	1.1(6)
C23-C22-C27-O15	-1.4(9)
C23-C22-C27-O16	-180.0(5)
C23-C22-N4-C26	0.8(8)
C23-C22-N4-Cu2	179.6(4)
C23-C24-C25-C26	1.0(8)
C24-C25-C26-C28	-179.6(5)
C24-C25-C26-N4	0.4(7)
C25-C26-C28-O13	1.8(8)
C25-C26-C28-O14	-179.6(5)
C25-C26-N4-C22	-1.3(8)
C25-C26-N4-Cu2	179.9(3)
C26-C28-O14-Cu2	-0.6(5)
C27-C22-C23-C24	179.3(5)
C27-C22-N4-C26	-178.0(4)
C27-C22-N4-Cu2	0.7(6)
C28-C26-N4-C22	178.6(4)
C28-C26-N4-Cu2	-0.1(5)
N3-C15-C16-C17	0.0(7)
N3-C15-C20-O9	0.7(7)
N3-C15-C20-O10	179.7(4)
N3-C19-C21-O11	179.2(4)
N3-C19-C21-O12	-1.1(6)
N4-C22-C23-C24	0.6(8)
N4-C22-C27-O15	177.3(5)
N4-C22-C27-O16	-1.2(7)
N4-C26-C28-O13	-178.1(4)
N4-C26-C28-O14	0.5(6)
O10-C20-O9-Cu2	-179.7(4)
O11-C21-O12-Cu2	-178.5(4)
O13-C28-O14-Cu2	177.9(4)
O15-C27-O16-Cu2	-177.3(5)

Symmetry transformations used to generate equivalent atoms:

Table 7. Hydrogen bonds for complex-5 [Å and °].

D-H...A	d(D-H)	d(H...A)	d(D...A)	<(DHA)
O2-H2A...O17#1	0.82	1.71	2.461(7)	150.7
O3-H3A...O19	0.82	1.64	2.446(6)	165.1
O10-H10A...O21#2	0.82	1.76	2.508(5)	151.0
O11-H11A...O22	0.84	1.76	2.598(5)	176.7
O19-H19A...O6#2	0.85	1.83	2.671(6)	170.1
O19-H19B...O20	0.85	1.79	2.533(7)	145.4
O21-H21A...O13#3	0.85	1.97	2.722(5)	146.3
O21-H21B...O6#2	0.85	1.88	2.652(6)	149.8
O17-H17A...O18	0.85	2.03	2.669(8)	131.8
O17-H17B...O22#4	0.85	2.22	3.017(6)	157.2
O18-H18A...O15#5	0.85	1.91	2.734(6)	162.0
O18-H18B...O7	0.85	1.98	2.826(7)	174.6
O22-H22A...O13#1	0.85	2.02	2.822(5)	157.8
O22-H22B...O16#6	0.85	1.88	2.728(5)	172.1

Symmetry transformations used to generate equivalent atoms:

#1 x,-y,z+1/2 #2 x,-y+1,z-1/2 #3 x,y+1,z

#4 x+1,y,z #5 x+1,-y+1,z+1/2 #6 x,-y+1,z+1/2
